# Effect of Particulate Matter Pollution on Global Lung Cancer Burden: A Systematic Analysis for the Global Burden of Disease Study 1990–2021

**DOI:** 10.1111/1759-7714.70174

**Published:** 2025-11-12

**Authors:** Yuhao Chen, Xinyue Yang, Hongbin Zhang, Xiuwen Zhang, Zixuan Hu, Zhiqiang Zhang, Yongwen Li, Hongyu Liu, Yaguang Fan, Jun Chen

**Affiliations:** ^1^ Department of Lung Cancer Surgery Tianjin Medical University General Hospital Tianjin People's Republic of China; ^2^ Department of Cardiology Tianjin Medical University General Hospital Tianjin China; ^3^ Tianjin Key Laboratory of Lung Cancer Metastasis and Tumor Microenvironment, Tianjin Lung Cancer Institute, Tianjin Medical University General Hospital Tianjin People's Republic of China

**Keywords:** age‐period‐cohort analysis, global burden, lung cancer, particulate matter pollution, sociodemographic index

## Abstract

**Background:**

Lung cancer remains a leading cause of cancer mortality globally, with particulate matter pollution (PMP) identified as a critical environmental risk. This study analyzes long‐term trends in age‐standardized mortality (ASMR) and disability‐adjusted life‐year rates (ASDR) for PMP‐attributable lung cancer, with projections to 2030.

**Method:**

Using Global Burden of Disease data, we evaluated temporal trends across age, sex, and Sociodemographic Index (SDI) regions through age‐period‐cohort and Bayesian models.

**Result:**

Global Trends: PMP‐related ASMR/ASDR declined significantly over the study period, while ambient PMP (APMP)‐attributable rates increased, contrasting with household air pollution (HAP)‐related declines. Age and Sex Disparities: Mortality burden shifted toward older populations, with APMP‐related deaths rising sharply in the elderly. Males exhibited faster declines in PMP/HAP‐related mortality, whereas females faced steeper increases in APMP‐attributable risks. SDI variations: High‐middle SDI regions consistently had the highest PMP‐related mortality, with ASMR trends reflecting industrialization phases. Projections: PMP‐related burdens are expected to rise globally, driven by aging populations and persistent pollution in middle‐SDI regions.

**Conclusion:**

The escalating burden in vulnerable populations demands urgent interventions, including air quality improvement, tobacco control, and enhanced screening, Notably, China consistently exhibited the world's highest PMP‐attributable lung cancer ASMR (13.6 per 100 000 in 1990, declining to 10.1 per 100 000 in 2021). Future strategies must integrate gender‐specific risk mitigation and environmental‐genetic assessments to address disparities.

## Graphical abstract

(A) Main Sources of PMP, APMP, and HAP and the Background of Particulate Pollution Leading to Lung Cancer. (B) Analytical Methods and Results from the GBD Database. (C) Increase in Lung Cancer Burden from PMP and APMP Between 1990 and 2020, Decrease in Lung Cancer Burden from HAP Over the Same Period. (D) Lung Cancer Burden from Particulate Pollution Increases in the Elderly, Decreases in Males, and Increases in Females. (E) Lung Cancer Burden from Particulate Pollution in Different SDI Regions, Displayed by Bubble Size, with High and Low SDI Regions Having Heavier Burden. (F) Projection for 2030: The Lung Cancer Burden from PMP, APMP, and HAP Will All Increase. Abbreviations: APMP, ambient particulate matter pollution; HAP, household air pollution from solid fuels; GBD, global burden of disease; PMP, particulate matter pollution; SDI, sociodemographic index.

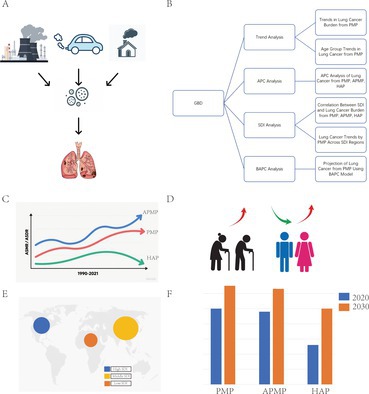

**Abbreviations**
APMP: ambient particulate matter pollution
GBD: global burden of disease
HAP: household air pollution from solid fuels
PMP: particulate matter pollution
SDI: sociodemographic index

## 1 Introduction

Lung cancer has one of the highest incidence and mortality rates among all malignant tumors worldwide, hence making it one of the most common cancers. Air pollution, as a major global public health issue, has been shown by numerous studies to be closely related to the occurrence and development of lung cancer. In recent years, air pollution has become one of the reasons for the high incidence of respiratory diseases. Fine particulate matter (PM2.5), with a diameter of ≤ 2.5 μm, is the main pollutant affecting air quality. The International Agency for Research on Cancer (IARC) has classified PM2.5 as a Group 1 carcinogen [1]. Studies have shown that PM2.5, with its complex composition and the ability to adsorb various harmful substances, increases the incidence and mortality of respiratory and cardiovascular diseases and cancer [2]. The soluble substances, low volatile or non‐volatile organic compounds, metal ions, and inorganic salts loaded on fine particles enable them to be easily deposited within tissues and cells, leading to changes in the lung tissue and cellular microenvironment, thus inducing genetic toxicity. The carcinogenic potential of PM2.5 arises through several interconnected mechanisms. Upon inhalation and deposition in the lungs, PM2.5 components, particularly polycyclic aromatic hydrocarbons (PAHs), heavy metals (e.g., arsenic, chromium, nickel), and reactive oxygen species (ROS), can directly damage DNA through the formation of DNA adducts and oxidative base modifications [3, 4]. This direct genotoxicity can lead to mutations in critical genes involved in cell cycle control, DNA repair, and apoptosis [3, 4]. Furthermore, PM2.5 exposure induces chronic inflammation in the lung tissue. Activated macrophages and epithelial cells release pro‐inflammatory cytokines (e.g., IL‐6, TNF‐α) and chemokines, creating a microenvironment rich in ROS and reactive nitrogen species (RNS) that further exacerbate oxidative stress and DNA damage [5, 6]. This sustained inflammatory response promotes cell proliferation and inhibits apoptosis, providing a selective advantage for pre‐malignant cells. PM2.5 exposure has also been linked to epigenetic alterations, such as changes in DNA methylation and microRNA expression profiles, which can silence tumor suppressor genes or activate oncogenes without altering the DNA sequence itself [7]. These combined mechanisms—direct DNA damage, chronic inflammation‐driven genomic instability, and epigenetic dysregulation—contribute significantly to the initiation and promotion of lung cancer development associated with PM2.5 exposure. These factors can further cause diseases such as bronchial asthma, chronic obstructive pulmonary disease, and lung cancer [8]. The primary contributor to PM2.5 is ambient particulate matter pollution (APMP). Numerous studies have demonstrated a strong relationship between particulate matter (PM2.5) pollution and lung cancer [9, 10]. Studies have found that outdoor air pollution is estimated to cause 8% of lung cancer deaths worldwide [11]. Apart from outdoor air pollution, household fine particulate matter pollution (HAP) can also lead to lung cancer [12]. Air pollution is a heterogeneous and complex mixture of gases, liquids, and PM. An increasing body of evidence has satisfactorily reported the association between short‐ and long‐term exposure to environmental PM2.5 and mortality or morbidity in cohort studies worldwide [13, 14, 15, 16, 17].

The focus on environmental PMP, APMP, and HAP in this study is driven by their distinct yet significant roles in exacerbating respiratory health issues. Environmental PMP (ambient particulate matter pollution) and APMP (ambient particulate matter pollution) represent the broader environmental sources of PM2.5 exposure, including industrial emissions, vehicle exhaust, and natural sources such as wildfires, while HAP (household air pollution) arises from indoor activities such as cooking and heating, particularly in low‐ and middle‐income countries. These categories were chosen for analysis because they collectively cover the spectrum of PM2.5 exposure sources, both outdoor and indoor, that have been implicated in the highest rates of lung cancer and respiratory diseases. Understanding the specific contributions of each category to the disease burden is essential for targeted public health interventions and pollution control strategies. In addition, assessing the impact of both environmental and household pollution will enable the development of comprehensive policies to reduce lung cancer risk across different populations and settings.

As with previous Global Burden of Disease (GBD) studies, cause‐specific death rates for most causes were estimated using the Cause of Death Ensemble model. This is a modeling tool developed for GBD to assess the out‐of‐sample predictive validity of different statistical models and covariate permutations and combine those results to produce cause‐specific mortality estimates, with alternative strategies adapted to model causes with insufficient data, substantial changes in reporting over the study period, or unusual epidemiology [18]. The traditional measures of disease status, such as mortality and incidence rates, are not adequate for comparing the overall impact of diseases on population health. This is because mortality fails to account for the non‐fatal consequences of diseases, while incidence does not consider the duration and severity of the disability caused by diseases. Therefore, the introduction of a new evaluation standard is essential. Disability‐Adjusted Life Years (DALYs) can measure the burden of disease on a community; the more DALYs lost due to a disease, the greater the burden that disease imposes on the community.

A detailed update on the global, regional, and national burdens related to environmental PMP, APMP, and HAP is essential for prevention and control, especially as global exposure levels to environmental PMP, APMP, and HAP continue to increase. The GBD is a systematic and up‐to‐date global epidemiological study that aims to quantify the mortality, incidence, and disability rates of major diseases, injuries, and risk factors by location, sex, age, and year [19]. This article uses the latest GBD 2021 dataset to summarize the burden attributed to environmental PMP, APMP, and HAP by region, sex, and age, and provides a useful reference for developing strategies to control environmental PMP, APMP, and HAP.

## 2 Methods

### 2.1 Data Source

We used data from the 2021 Global Burden of Disease (GBD) study, adhering to its methodological framework and analytical strategies. GBD 2021 provides epidemiological descriptions and estimates for 369 diseases and injuries across 204 countries and regions from 1990 to 2021. This approach leverages existing data to compensate for incomplete healthcare information, aiding in the estimation of the GBD for various regions [19]. Ethical considerations for the study adhered to the tenets of the Declaration of Helsinki, and the need for informed consent was waived by the institutional review board of the University of Washington owing to the use of de‐identified aggregated data. The study follows the guidelines of the GATHER checklist for accurate and transparent health estimates [20].

### 2.2 PMP Carcinogens Associated With Lung Cancer

The GBD study employs a comparative risk assessment method based on a causal framework and risk factor hierarchy, categorizing risk factors into four levels from broad (level 1, e.g., air pollution) to specific (level 4, e.g., APMP). For our study, PMP (level 3 risk) was defined using GBD criteria and included PM2.5 from APMP and HAP from the use of solid fuels like coal, charcoal, wood, agricultural residue, and animal dung. APMP refers to particulate matter from ambient sources such as vehicular emissions, industrial activities, and natural events like wildfires and dust storms, while HAP mainly arises from indoor combustion of solid fuels in households, particularly in low‐ and middle‐income countries. These sources are included in the GBD framework due to their established links to respiratory diseases and lung cancer. However, sources such as PM2.5 from tobacco smoke or other indoor activities unrelated to solid fuel use are excluded, and the model may not fully capture regional variations, such as localized industrial pollution or specific agricultural practices, that could affect lung cancer risk.

### 2.3 Disease Burden Metrics

This study analyzed 30‐year mortality trends (1990–2021) using data from the Global Burden of Disease (GBD) 2021, including Disability‐Adjusted Life Years (DALYs), combining Years of Life Lost (YLL) and Years Lived with Disability (YLD), deaths, all‐age mortality rates, age‐standardized mortality rates (ASMR), age‐standardized DALY rates (ASDR), and relative percentage changes. The population was stratified into six age groups (25–39, 40–49, 50–59, 60–69, 70–79, > 80 years), with mortality calculations starting at age 25 due to negligible PMP‐attributable lung cancer deaths in younger cohorts.

### 2.4 Statistical Analysis

Temporal trends were analyzed using Estimated Annual Percentage Change (EAPC) via log‐linear regression and Net Drift derived from an Age‐Period‐Cohort (APC) model. The APC framework decomposed the overall trends into three components: (1) Age effects, reflecting intrinsic risk variations across age groups, such as increasing lung cancer mortality with aging; (2) Period effects, capturing influences from calendar year–specific factors like advancements in diagnosis or environmental policies; (3) Cohort effects, representing generational differences due to varied cumulative exposures to particulate matter pollution (PMP), ambient PM pollution (APMP), and household air pollution (HAP). Net Drift, calculated as the sum of period and cohort coefficients (Net Drift = βperiod + γcohort), quantified the overall annual percentage change in mortality, independent of age distribution. This combined approach of EAPC and Net Drift allowed disentangling linear temporal trends from complex age‐cohort interactions, offering clearer insights for targeted interventions.

In this study, the APC model focused on individuals aged 50 and above due to a low number of deaths in younger populations. Age groups were categorized in 5‐year intervals from 50–54 to 85–89, and periods from 1990–1995 to 2015–2020. Birth cohorts ranged from 1895–1899 to 2000–2004. The intrinsic estimator (IE) method was employed within the APC model to estimate net effects across these three dimensions. While the IE method helps resolve the identifiability issue in APC models by separating age, period, and cohort effects, it relies on the assumption that these effects are linear and separable. However, this assumption may not always hold, particularly in complex datasets or when age, period, and cohort effects are confounded. Additionally, the IE method may introduce biases when dealing with sparse data. Therefore, while the IE method provides a practical solution, careful interpretation is required, particularly when estimating the effects of lung cancer mortality attributable to PMP, APMP, and HAP. see data, especially for smaller population subgroups. Relative risks (RR) and 95% confidence intervals (CI) were calculated based on estimated coefficients to quantify the impacts of age, period, and cohort on lung cancer mortality attributable to PMP, APMP, and HAP. The reference groups were defined as ages 50–54, period 1990–1994, and birth cohort 1890–1899.

The relationship between the age‐standardized mortality rate (ASMR) and the Socio‐demographic index (SDI) was analyzed using a regression model with a Gaussian process and a Locally Estimated Scatterplot Smoothing (LOESS) smoother. This relationship was evaluated using Spearman's rank correlation tests. A 95% uncertainty interval (UI) is reported for all metrics, with all ratios presented per 100 000 inhabitants.

Next, we used a Bayesian Age‐Period‐Cohort (BAPC) model to predict the global burden trends from 2020 to 2030 [21, 22]. Based on the assumption that the effects of age, period, and cohort were similar in temporal proximity, Bayesian inference in the BAPC model utilized a second‐order stochastic excursion to smooth the prior three aforementioned values and predict the posterior rates. The second‐order stochastic excursion approach provides greater flexibility in modeling temporal trends by smoothing abrupt fluctuations and capturing underlying patterns. This method improves model stability, particularly in the presence of noisy or sparse data, by reducing the risk of overfitting. However, it may also obscure more sudden, significant changes in trends, which could be relevant in certain contexts. Additionally, the effectiveness of Bayesian inference depends on the choice of prior distributions, which can influence the results if not carefully selected. Despite these potential limitations, the use of second‐order stochastic excursion in BAPC enables more robust and reliable predictions of disease burden trends [23]. The population data were collected from the World Health Organization (WHO). BAPC used integrated nested Laplace approximations (INLA) to approximate the marginal posterior distributions, thus avoiding the mixing and convergence problems associated with traditional Bayesian methods using Markov chain Monte Carlo methods [24], which have been widely used to analyze trends in chronic diseases and predict disease burden in the future [21, 25]. All statistical analyses were conducted using R version 4.3.1 software (R Foundation for Statistical Computing, Vienna, Austria). The packages used for this study included “BAPC” and “INLA”. Statistical significance was set at *p* < 0.05.

## 3 Results

### 3.1 Trends in the Global Burden of Lung Cancer Attributable to PMP

**FIGURE 1:**
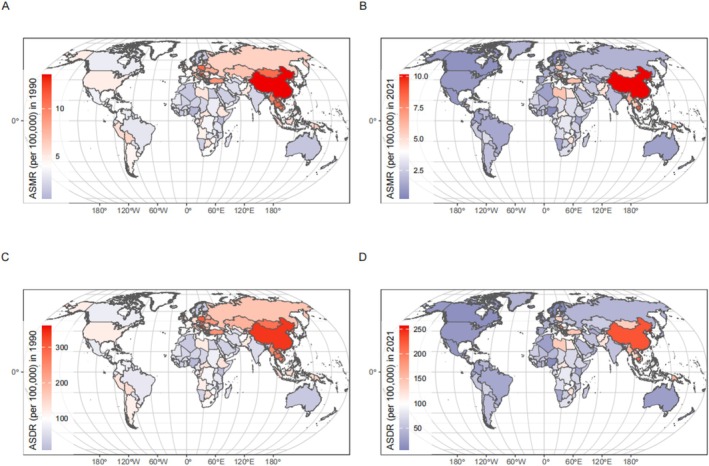
The global distribution of ASMR of lung cancer attributable to PMP in 1990 (A) and 2021 (B). The global distribution of ASDR of lung cancer attributable to PMP in 1990 (C) and 2021 (D). Abbreviations: ASDR, Age‐standardized DALY rate; ASMR, Age‐standardized mortality rate; DALY, Disability‐adjusted life years.

Between 1990 and 2021, the global percent change in the number of lung cancer cases caused by APMP increased significantly, while the number caused by HAP decreased, and those caused by PMP saw a rise. The percent change in the all‐age mortality rate because of lung cancer due to APMP and HAP followed a similar trend, whereas PMP showed a slight decline. The percent change in the ASMR of lung cancer due to APMP saw a small increase, while those due to PMP and HAP showed varying degrees of decline (Table 1). In 1990, at the national level, the ASMR was as follows: In China, the number of deaths from lung cancer caused by PMP was the highest in the world in 1990, at 13.6 (95% UI: 9.2–18) per 100 000. The second and third highest countries were Bosnia and Herzegovina and Bahrain, at 13.3 (95% UI: 8.7–17.8) and 12.6 (95% UI: 8.2–16.9) per 100 000, respectively. In 2021, China still had the highest mortality at 10.1 (95% UI: 6.3–14.4) per 100 000, followed by the Democratic People's Republic of Korea and Cambodia, at 9.8 (95% UI: 5.3–15.5) and 8.9 (95% UI: 5.4–13.3) per 100 000 respectively (Figure 1A,B and Table S1).

**TABLE 1:** The global trends in the lung cancer burden of death attributable to PMP, APMP, and HAP by sex from 1990 to 2021 globally.

Risk	Sex	Deaths		All‐age mortality		Age‐standardized mortality		Net drift of mortality^b^, % per year
		Number in 2021^a^, *n*	Percent change of numbers 1990–2021, %	Rate in 2021, per 100 000	Percent change of numbers 1990–2021, %	Rate in 2021, per 100 000	Percent change 1990–2021, %	
PMP	Male	252 418 (157 293 to 356 203)	36.78 (4.15 to 70.2)	6.4 (4 to 9)	−7.22 (−29.35 to 15.45)	6.4 (4 to 9)	−37.83 (−52.54 to −22.94)	−1.64 (−1.72 to −1.56)
PMP	Female	121 795 (76 047 to 168 791)	77.16 (45.02 to 119.46)	3.1 (1.9 to 4.3)	19.3 (−2.34 to 47.79)	2.6 (1.6 to 3.6)	−18.7 (−33.52 to 0.76)	−1.11 (−1.16 to −1.05)
PMP	Both	374 213 (236 358 to 520 255)	47.74 (18.95 to 76.44)	4.7 (3 to 6.6)	−0.14 (−19.61 to 19.25)	4.3 (2.7 to 6)	−32.12 (−45.21 to −18.82)	−1.44 (−1.51 to −1.36)
APMP	Male	203 793 (124 626 to 289 178)	107.76 (49.28 to 179.25)	5.1 (3.1 to 7.3)	40.93 (1.26 to 89.42)	5.2 (3.2 to 7.3)	−6.68 (−32.77 to 25.78)	0.02 (−0.07 to 0.12)
APMP	Female	93 805 (54 038 to 136 098)	219.98 (138.68 to 331.08)	2.4 (1.4 to 3.5)	115.48 (60.73 to 190.3)	2 (1.2 to 2.9)	45.21 (8.64 to 95.33)	1.28 (1.2 to 1.36)
APMP	Both	297 598 (183 711 to 414 740)	133.58 (73.26 to 208.75)	3.8 (2.3 to 5.3)	57.87 (17.1 to 108.68)	3.5 (2.1 to 4.8)	6.33 (−21.01 to 40.56)	0.41 (0.32 to 0.5)
HAP	Male	48 540 (17 739 to 118 517)	−43.83 (−71.8 to 11.55)	1.2 (0.4 to 3)	−61.9 (−80.87 to −24.33)	1.2 (0.4 to 2.9)	−74.56 (−87.31 to −49.12)	−4.62 (−4.73 to −4.51)
HAP	Female	27 942 (10 569 to 68 163)	−29.12 (−66.84 to 41.41)	0.7 (0.3 to 1.7)	−52.27 (−77.67 to −4.77)	0.6 (0.2 to 1.5)	−67.07 (−84.5 to −34.52)	−4.16 (−4.24 to −4.09)
APMP	Male	203 793 (124 626 to 289 178)	107.76 (49.28 to 179.25)	5.1 (3.1 to 7.3)	40.93 (1.26 to 89.42)	5.2 (3.2 to 7.3)	−6.68 (−32.77 to 25.78)	0.02 (−0.07 to 0.12)

*Note:* The all‐age mortality is equivalent to the crude DALY rate.

Abbreviations: APMP, ambient particulate matter pollution; HAP, household air pollution from solid fuels; PMP, particulate matter pollution.

^a^ The parentheses accompanying all Global Burden of Disease health estimates represent 95% uncertainty intervals, while the parentheses for net drift indicate 95% confidence intervals.

^b^ The net drifts are estimates derived from the APC model and signify the overall annual percentage change in mortality, encompassing the effects from calendar time and successive birth cohorts.

Between 1990 and 2021, the global percent change in the DALY number due to lung cancer caused by APMP increased significantly, while those caused by PMP and HAP experienced a moderate increase and a certain degree of decline, respectively. The percent change in the all‐age DALY rate owing to lung cancer caused by APMP showed an increase, whereas those caused by PMP and HAP showed a moderate decline. The percent change in the age‐standardized DALY rate (ASDR) of lung cancer due to PMP, APMP, and HAP all showed varying degrees of decline (Table 2). In 1990, the highest DALYs owing to lung cancer caused by PMP were observed in Bosnia, followed by China and Poland. By 2021, the highest DALYs owing to lung cancer caused by PMP were in the Democratic People's Republic of Korea, China, and the Solomon Islands (Figure 1C,D and Table S1).

**TABLE 2:** The global trends in the lung cancer burden of DALYs attributable to PMP, APMP, and HAP by sex from 1990 to 2021 in global.

Risk	Sex	DALYs		All‐age DALY rate		Age‐standardized DALY rate		Net drift of DALY rate^b^, % per year
		Number in 2021^a^, *n*	Change of numbers 1990–2021, %	Rate in 2021, per 100 000	Percent change 1990–2021, %	Rate in 2021, per 100 000	Percent change 1990–2021, %	
PMP	Male	6094329.6 (3813183.1 to 8640721.9)	20.84 (−7.93 to 50.55)	153.9 (96.3 to 218.2)	−18.03 (−37.55 to 2.12)	146.9 (91.8 to 207.6)	−43.04 (−56.55 to −29)	−1.64 (−1.75 to −1.53)
PMP	Female	2839790.7 (1788757.9 to 3943288.5)	55.98 (26.54 to 94.72)	72.2 (45.5 to 100.3)	5.04 (−14.79 to 31.13)	62 (39.1 to 86.1)	−26 (−39.95 to −7.65)	−1.1 (−1.16 to −1.04)
PMP	Both	8934120.3 (5681090.3 to 12409780.5)	30.16 (4.66 to 56.33)	113.2 (72 to 157.3)	−12.02 (−29.26 to 5.66)	102.1 (64.9 to 141.6)	−38.33 (−50.43 to −25.91)	−1.43 (−1.53 to −1.34)
HAP	Male	1260319.3 (475009.5 to 2979785.3)	−48.1 (−72.9 to 0.6)	31.8 (12 to 75.3)	−64.8 (−81.62 to −31.76)	30 (11.2 to 71.2)	−75.53 (−87.28 to −52.46)	−4.61 (−4.75 to −4.47)
HAP	Female	706106.5 (279197.1 to 1645019.9)	−35.19 (−68.17 to 25.49)	18 (7.1 to 41.8)	−56.36 (−78.57 to −15.49)	15.5 (6.2 to 36)	−68.91 (−84.64 to −40.08)	−4.15 (−4.24 to −4.07)
HAP	Both	1966425.7 (758404.9 to 4632387.1)	−44.1 (−70.66 to 3.17)	24.9 (9.6 to 58.7)	−62.22 (−80.17 to −30.27)	22.4 (8.6 to 52.9)	−73.36 (−86.02 to −50.85)	−4.42 (−4.52 to −4.31)
APMP	Male	4831943.9 (2949781.2 to 6838503.2)	84.86 (32.19 to 149.11)	122 (74.5 to 172.7)	25.39 (−10.34 to 68.97)	116.9 (71.4 to 165.5)	−13.66 (−38.09 to 16.19)	0.03 (−0.1 to 0.16)
APMP	Female	2 132 587 (1234993.9 to 3080469.9)	191.8 (113.72 to 294.07)	54.2 (31.4 to 78.3)	96.51 (43.92 to 165.37)	46.5 (26.9 to 67.1)	37.22 (0.61 to 85.27)	1.29 (1.21 to 1.38)
APMP	Both	6964530.9 (4284313.5 to 9 719 777)	108.22 (53.34 to 175.73)	88.3 (54.3 to 123.2)	40.73 (3.64 to 86.36)	79.6 (49 to 111.2)	−2.14 (−27.71 to 29.55)	0.42 (0.3 to 0.54)

*Note:* The all‐age mortality is equivalent to the crude mortality rate.

Abbreviations: APMP, ambient particulate matter pollution; DALYs, Disability‐adjusted life years; HAP, household air pollution from solid fuels; PMP, particulate matter pollution.

^a^ The parentheses accompanying all Global Burden of Disease health estimates represent 95% uncertainty intervals, while the parentheses for net drift indicate 95% confidence intervals.

^b^ The net drifts are estimates derived from the APC model and signify the overall annual percentage change in DALYs, encompassing the effects from calendar time and successive birth cohorts.

### 3.2 Temporal Trends of the Burden of Lung Cancer Caused by PMP Across Age Groups in Global

**FIGURE 2:**
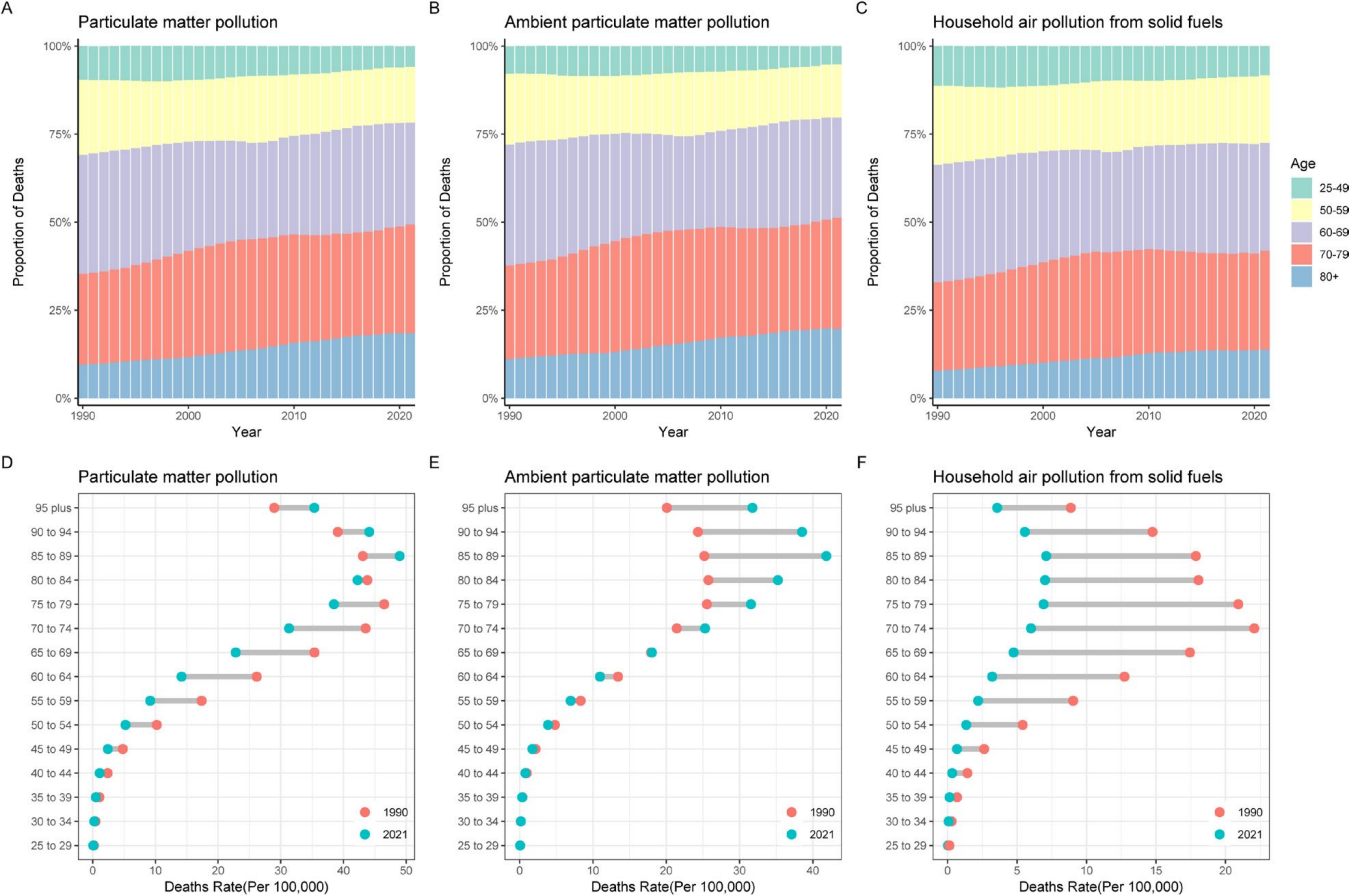
The temporal change of the mortality rate attributed to lung cancer‐related mortality attributable to PMP, APMP, and HAP across age groups globally from 1990 to 2021. (A–C) The relative proportion of lung cancer‐related mortality attributable to PMP, APMP, and HAP; (D–F) The temporal changes in the mortality rate of lung cancer attributable to PMP, APMP, and HAP. Abbreviations: APMP, ambient particulate matter pollution; HAP, household air pollution from solid fuels; PMP, particulate matter pollution.

In the past 30 years, among the global population affected by particulate pollutants leading to lung cancer, the mortality rates of all subtypes of lung cancer have gradually shifted toward the older age groups (≥ 50 years), which is a marker of particulate matter exposure‐associated lung cancer burden demonstrating significant age‐specific clustering, with individuals aged ≥ 50 years constituting the primary risk‐bearing population for this etiological factor (Figure 2A–C). From 1990 to 2021, worldwide, the mortality rate of lung cancer owing to PMP has decreased among individuals < 80 years old. Specifically, for APMP and HAP, lung cancer mortality rates have risen across all age groups owing to APMP, with a notable increase in those > 70 years. For HAP, mortality rates have also decreased across all age groups, with a significant decrease starting from those > 40 years old (Figure 2D–F).

**FIGURE 3:**
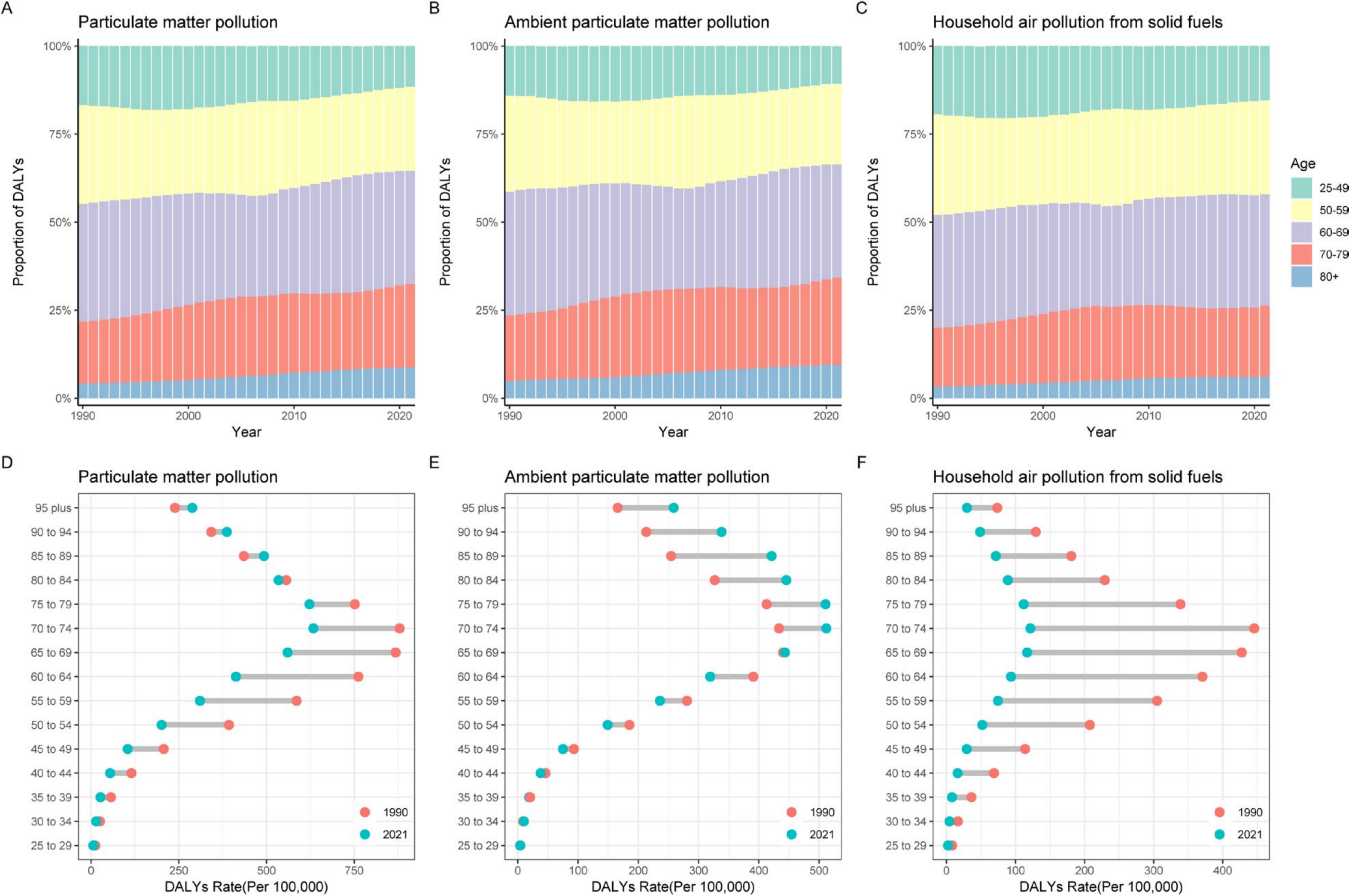
The global trends in DALY rates for lung cancer‐related mortality due to PMP, APMP, and HAP across different age groups from 1990 to 2021. (A–C) The relative proportion of lung cancer‐related mortality attributable to PMP, APMP, and HAP; (D–F) the temporal changes in the mortality rate of lung cancer attributable to PMP, APMP, and HAP. Abbreviations: APMP, ambient particulate matter pollution; DALY, Disability‐adjusted life years; HAP, household air pollution from solid fuels; PMP, particulate matter pollution.

Over the past 30 years, globally, the proportion of DALYs owing to lung cancer caused by PMP has significantly increased in those aged > 50 years (Figure 3A). The DALYs rate (per 100 000) has significantly decreased in populations < 80 years old, while it has increased in those > 80 years old (Figure 3D). Specifically, the proportion of DALYs due to APMP has significantly increased in those > 70 years old, remained relatively stable in the 50–59 age group, and decreased significantly in the 25–49 age group (Figure 3B). The DALYs rate (per 100 000) decreased in individuals > 65 years old, showed no significant change in the 65–69 age group, and increased in those < 65 years old (Figure 3E). For HAP, the proportion of DALYs decreased in the 25–49 age group, increased in those > 80 years old, with no significant changes in the other age groups (Figure 3C). The DALYs rate (per 100 000) decreased across all age groups, with a notable decline in the 55–79 age group (Figure 3F).

### 3.3 APC Analysis of the Lung Cancer Burden Attributable to PMP, APMP, and HAP

**FIGURE 4:**
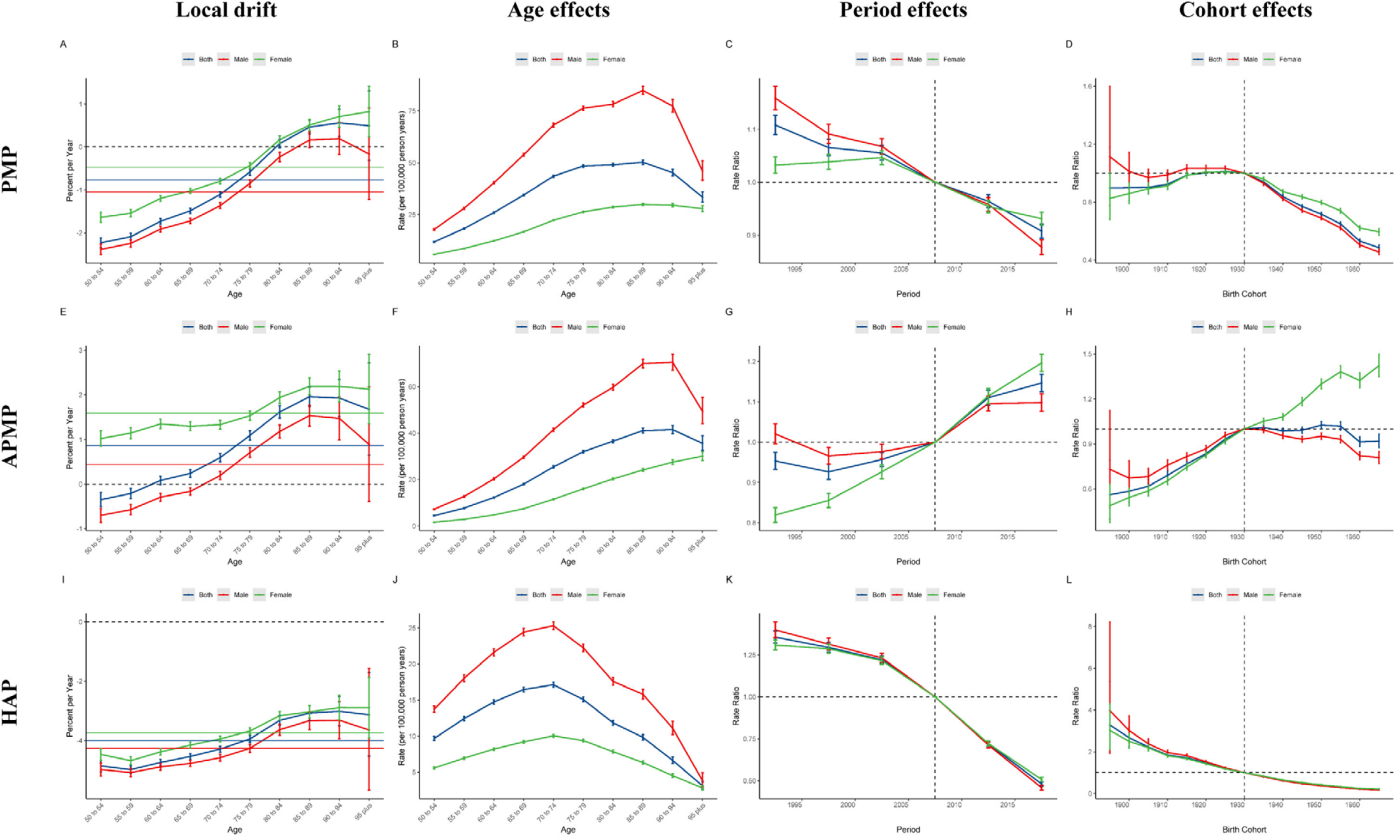
The local drifts, age effects, period effects, and cohort effects of lung cancer‐related mortality attributable to PMP, APMP, and HAP globally from 1990 to 2021. Abbreviations: APMP, ambient particulate matter pollution; HAP, household air pollution from solid fuels; PMP, particulate matter pollution.

Figure 4 provides valuable insights into measurements derived from the APC models for both male and female cohorts, as well as the three causes of lung cancer deaths associated with PMP, APMP, and HAP.

Overall, based on the APC model, the net drift in global lung cancer mortality rates due to PMP is −0.77% per year (95% CI: −0.85% to −0.69%); for APMP, the net drift is 0.87% per year (95% CI: 0.76% to 0.98%); and for HAP, the net drift is −4.00% per year (95% CI: −4.14% to −3.86%) (Table S2). However, there were differences between males and females in net drift. For lung cancer related to PMP, the decrease in mortality was slightly higher in males (−1.05% [95% CI: −1.15% to −0.95%]) than in females (−0.47% [95% CI: −0.53% to −0.40%]). For lung cancer mortality due to APMP, the net drift was slightly lower in males (0.44% [95% CI: 0.31%–0.57%]) than in females (1.59% [95% CI: 1.50%–1.69%]). For HAP, lung cancer mortality rates showed a net drift of −4.26% for males (95% CI: −4.45% to −4.08%) and − 3.74% for females (95% CI: −3.85% to −3.62%) (Table S2).

The local drift of lung cancer mortality caused by PMP was inversely correlated with people < 80 years old. The local drift of lung cancer mortality caused by APMP was positively associated with people > 60 years old and increased with age until it slightly declined in those > 95 years old. The local drift of lung cancer mortality caused by HAP was negative throughout (Figure 4A,E,I).

The longitudinal age distribution of lung cancer mortality rates caused by PMP by gender showed an upward trend with age, first peaking and then declining (Figure 4B). The mortality rate growth patterns were similar for males and females across PMP, APMP, and HAP, though the age at which peaks occur differed (Figure 4B,F,J and Table S3). Regarding period effects, the decrease in lung cancer mortality rates for both PMP and HAP followed similar patterns for both males and females (Figure 4C,K), while the trend for APMP was increasing (Figure 4G). Similarly, cohort effects indicated a positive decline in mortality rates for PMP and HAP, with similar declining trends for both sexes; whereas, APMP showed an increasing trend for females and an increase followed by a decline for males (Figure 4D,H,J).

**FIGURE 5:**
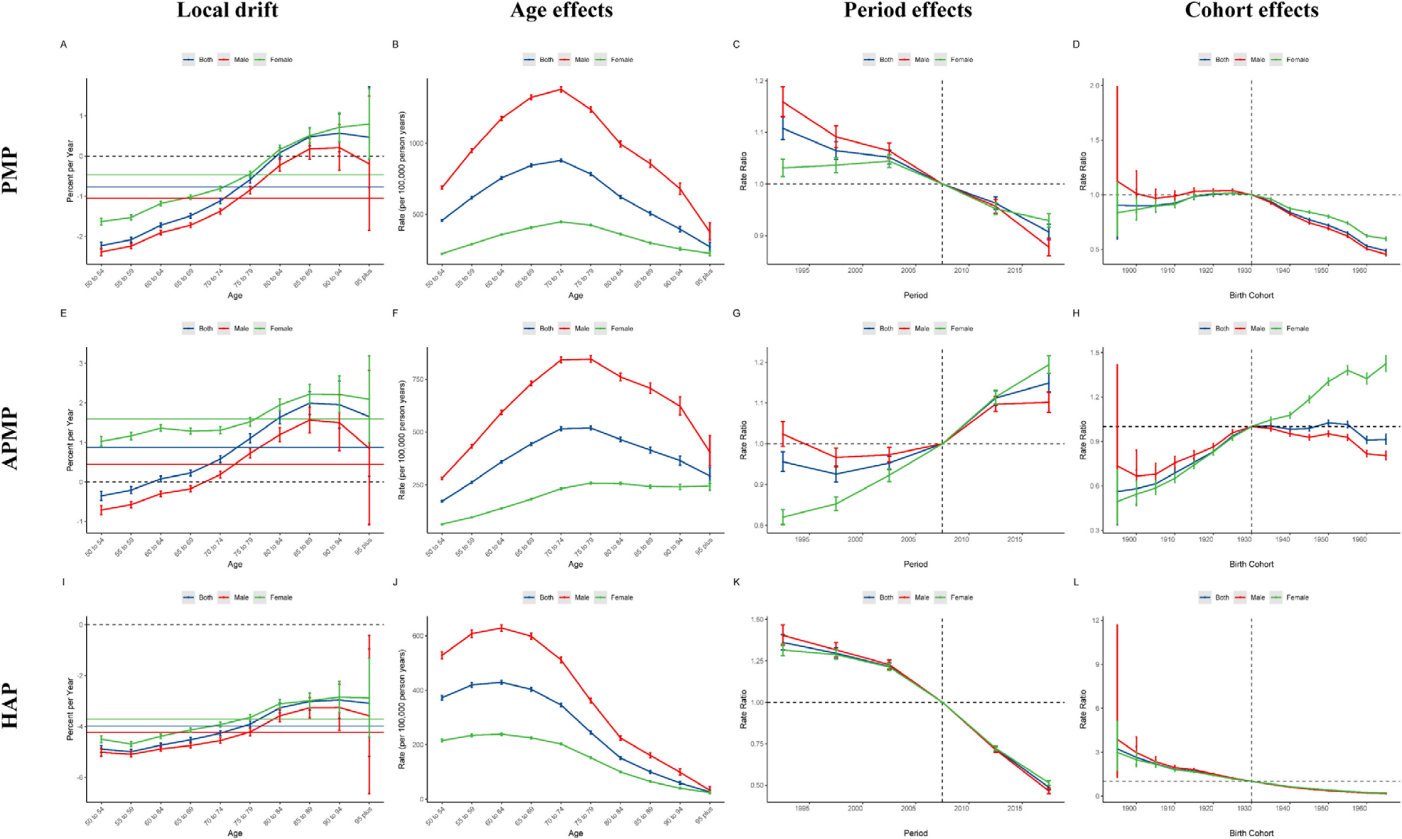
The local drifts, age effects, period effects, and cohort effects of lung cancer‐related DALY attributable to PMP, APMP, and HAP globally from 1990 to 2021. Abbreviations: APMP, ambient particulate matter pollution; DALY, Disability‐adjusted life years; HAP, household air pollution from solid fuels; PMP, particulate matter pollution.

Figure 5 shows the APC model used to assess the relationship between PMP, APMP, and HAP‐induced lung cancer and their DALYs burden. The results were largely consistent with the analysis of mortality, except for differences in the inflection points (Tables S4 and S5).

### 3.4 Trends in the Burden of Lung Cancer Attributable to PMP Across Different SDI Regions

In the past 30 years, the global number of lung cancer deaths due to PMP has increased by 47.74% (95% UI: 18.95%–76.44%), reaching 374212.7 (95% UI: 236358.3–520255.4) in 2021. PMP includes APMP and HAP (described in the Methods). The number of lung cancer deaths caused by APMP globally has increased by 133.58% (95% UI: 73.26%–208.75%); whereas, the number of deaths due to HAP has decreased by 39.22% (95% UI: −69.26% to −15.06%).

Among different SDI regions, the high‐middle SDI region had the highest number of lung cancer deaths due to PMP in 2021, reaching 124595.8 (95% UI: 79135.8–176646.6). This represents a 35.61% increase (95% UI: 8.05%–72.69%) from 1990 to 2021. Additionally, from 1990 to 2021, the global age‐specific mortality rate for lung cancer due to PMP decreased by 0.14% (95% UI: −19.61% to 19.25%) (Table 1). Among different SDI regions, the slowest decrease in lung cancer net drift caused by PMP was in the low SDI regions, at −0.24% (95% CI: −0.43% to −0.05%), while the fastest decrease was in the high SDI regions, at −2.76% (95% CI: −2.89% to −2.64%).

The global net drift for lung cancer burden caused by APMP was 0.41% (95% CI: 0.32%–0.5%). Among different SDI regions, the net drift of lung cancer caused by APMP increased the fastest in the middle SDI regions, at 2.61% (95% CI: 2.5%–2.71%), while it decreased the most in the High SDI regions, at −2.51% (95% CI: −2.64% to −2.37%). The global net drift for lung cancer caused by HAP is −4.43% (95% CI: −4.52% to −4.34%). Among different SDI regions, the net drift decreased the least in the low SDI regions, at −0.5% (95% CI: −0.72% to −0.28%), while the most significant decrease was in the high SDI regions, at −12.75% (95% CI: −13.74% to −11.75%) (Table S6). In 2021, the age‐standardized mortality rate for lung cancer due to PMP globally was 4.73 per 100 000 people (95% UI: 3–6.6). The percentage decrease in the global age‐standardized mortality rate from 1990 to 2021 was 32.12% (95% UI: −45.21% to −18.82%) (Table 1).

The DALY burden of lung cancer caused by PMP, APMP, and HAP is similar to the mortality burden caused by these factors, except for the fact that the highest DALY burden due to PMP‐caused lung cancer occurred in the middle SDI region (Table 2 and Table S7).

### 3.5 The Correlation Between SDI and the Burden of Lung Cancer Attributed to PMP, APMP, and HAP

**FIGURE 6:**
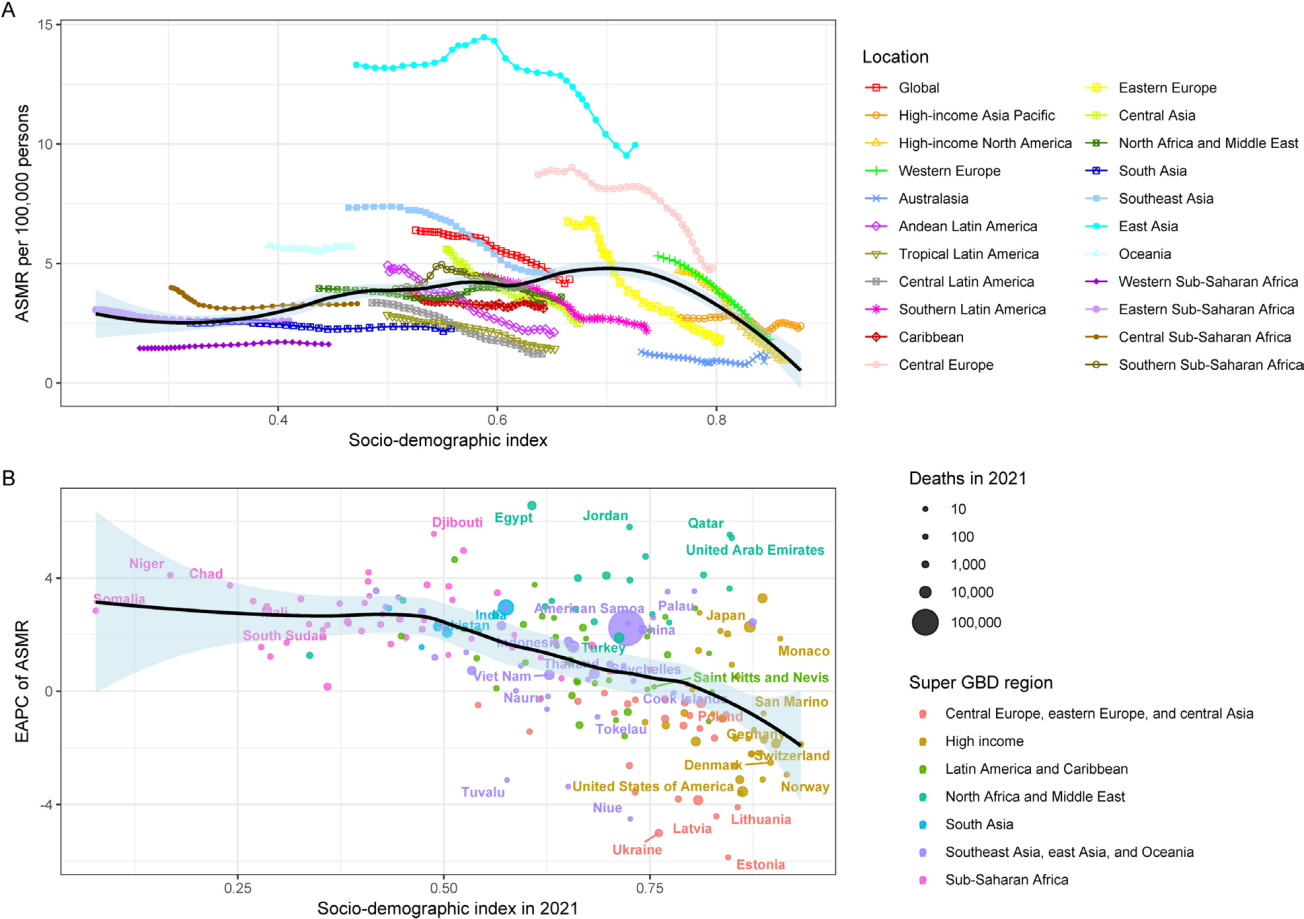
The correlation between PMP attributable lung cancer in ASMR and SDI. (A) The relationship between SDI levels and the ASMR for lung cancer attributable to PMP globally from 1990 to 2021. (B) The relationship between SDI levels and the EAPC in ASMR for lung cancer attributable to PMP globally from 1990 to 2021. Abbreviations: ASMR, age‐standardized mortality rate; EAPC, estimated annual percentage change; PMP, particulate matter pollution; SDI, sociodemographic index.

Overall, the association between ASMR from 1990 to 2021 for lung cancer caused by PMP and SDI shows a trend of inverted U curve, with a peak around an SDI of 0.7 (Figure 6A). From 1990 to2021, the Estimated Annual Percentage Change (EAPC) of ASMR was negatively correlated with SDI. With the increase of SDI, the EAPC of ASMR for lung cancer caused by PMP showed a continuous downward trend (Figure 6B). For lung cancer caused by APMP, the ASMR increased and then decreased with rising SDI, peaking at an SDI of around 0.7. The EAPC of ASMR showed a slow decline followed by a slow increase when SDI was < 0.5, and a decreasing trend when SDI exceeded 0.5 (Figure S1). For lung cancer caused by HAP, ASMR showed a slow decline with increasing SDI. The EAPC of ASMR was stable when the SDI was < 0.5, decreased sharply when the SDI was between 0.5 and 0.75, and then re‐stabilized (Figure S6).

**FIGURE 7:**
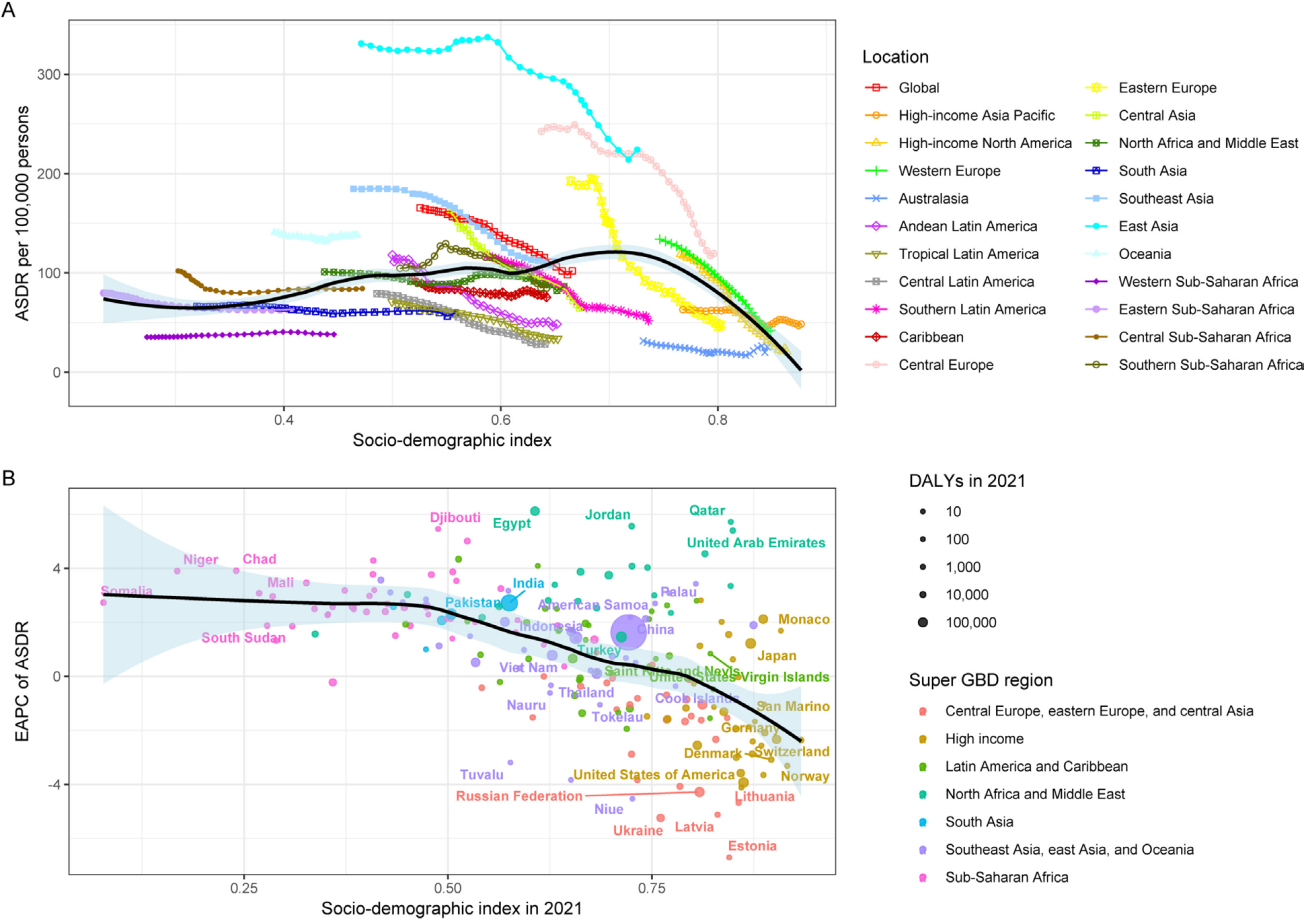
The correlation between PMP attributable lung cancer in ASDR and SDI (A) The relationship between SDI levels and the ASDR for lung cancer attributable to PMP globally from 1990 to 2021. (B) The relationship between SDI levels and the EAPC in ASDR for lung cancer attributable to PMP globally from 1990 to 2021. Abbreviations: ASDR, Age‐standardized DALY rate; DALY, disability‐adjusted life years; EAPC, estimated annual percentage change; PMP, particulate matter pollution; SDI, sociodemographic index.

The ASDR from 1990 to 2021 for lung cancer caused by PMP shows a trend of increasing and then decreasing with increasing SDI, peaking at an SDI of 0.7 (Figure 7A), with a slow decline in EAPC (Figure 7B). For lung cancer caused by APMP, ASDR increased and then decreased with rising SDI, and peaked around an SDI of 0.7. The EAPC of ASDR showed a slow decline followed by a slow increase when the SDI was < 0.5, and a rapid decrease when the SDI exceeded 0.5 (Figure S7). For lung cancer caused by HAP, ASDR decreased slowly with increasing SDI. The EAPC remained stable when the SDI was < 0.5, decreased rapidly when the SDI was between 0.5 and 0.7, and re‐stabilized when the SDI exceeded 0.7 (Figure S7).

### 3.6 Projection of Lung Cancer Burden From PMP by the BAPC Model

**FIGURE 8:**
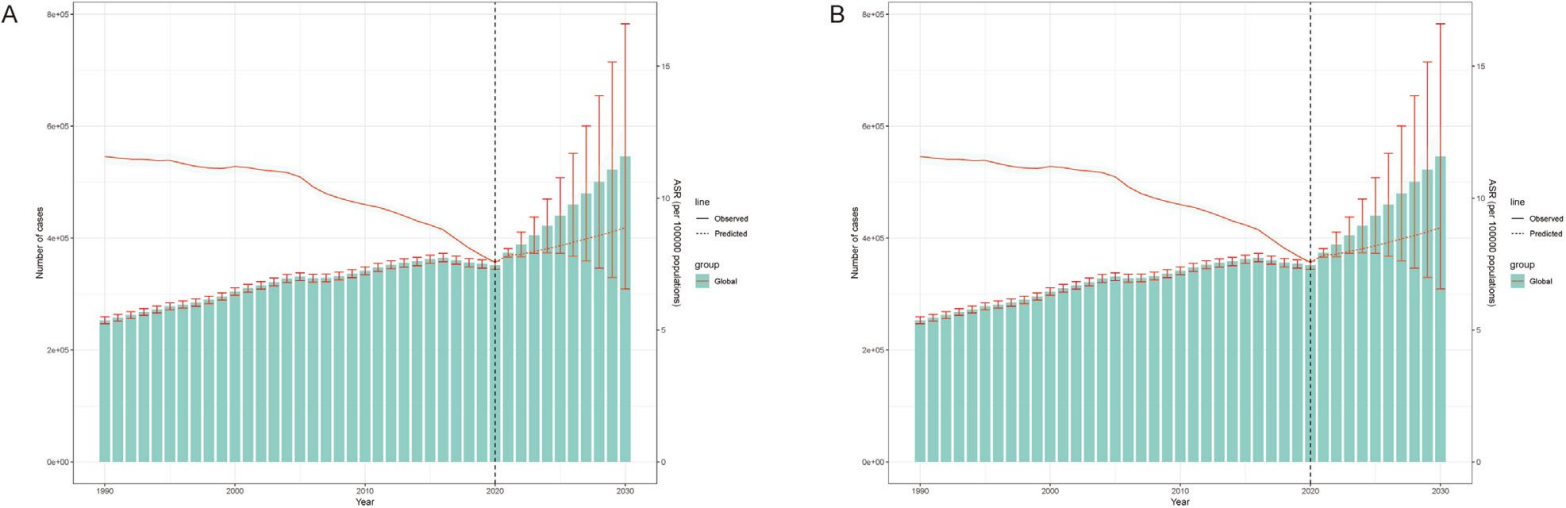
Global trends of lung cancer attributable to PMP in age‐standardized death (A) and DALYs (B) rates (per 100 000 population) from 2019 to 2030 by BAPC models: observed (solid lines) and predicted rates (dashed lines). Abbreviations: BAPC: Bayesian age‐period‐cohort; DALYs: Disability‐adjusted life years; GBD: Global Burden of Disease.

In general, the age‐standardized death and DALY rates of lung cancer attributable to PMP, APMP, and HAP were predicted to increase from 2020 to 2030 (Figures 8 and S5). Specifically, by 2030, the ASMR caused by PMP is projected to increase from 7.56 per 100 000 (95% UI: 7.40–7.72) in 2020 to 8.88 per 100 000 (95% UI: 5.02–12.73) in 2030 (Figure 8A and Table S8), and the ASDR is expected to rise from 177.72 per 100 000 (95% UI: 176.91–178.54) in 2020 to 197.48 per 100 000 (95% UI: 113.07–281.89) in 2030 (Figure 8B and Table S8). The ASMR caused by APMP is projected to increase from 6.07 per 100 000 (95% UI: 5.93–6.21) in 2020 to 6.59 per 100 000 (95% UI: 3.92–9.26) in 2030 (Figure S5A and Table S8), and the ASDR is expected to rise from 139.59 per 100 000 (95% UI: 138.87–140.31) in 2020 to 141.15 per 100 000 (95% UI: 84.54–188.99) in 2030 (Figure S5B and Table S8). The ASMR caused by HAP is projected to increase from 1.50 per 100 000 (95% UI: 1.43–1.57) in 2020 to 2.15 per 100 000 (95% UI: 0.59–3.71) in 2030 (Figure S5C and Table S8), and the ASDR is expected to rise from 38.078 per 100 000 (95% UI: 37.70–38.46) in 2020 to 53.75 per 100 000 (95% UI: 14.76–92.75) in 2030 (Figure S5D and Table S8). Overall, the number of deaths and DALYs caused by lung cancer due to PMP, APMP, and HAP is expected to increase over the next decade.

## 4 Discussion

This study examines the global burden of lung cancer caused by PMP, APMP, and HAP. In summary, the lung cancer burden caused by PMP and HAP is decreasing, while the burden caused by APMP is increasing. Lung cancer resulting from these three types of particulate pollutants is becoming more concentrated in people > 50 years old. After evaluating with the APC model, the results show that the lung cancer burden from PMP, APMP, and HAP is still increasing annually, but the proportion of the burden caused by APMP is rising, while the proportions caused by PMP and HAP are declining. Our study also explores the relationship between the SDI ASMR and ASDR for lung cancer caused by PMP, APMP, and HAP. The results showed that the ASMR and ASDR of lung cancer caused by PMP and APMP first increase and then decrease with the increase in SDI, whereas the ASMR and ASDR of lung cancer caused by HAP consistently decrease as the SDI increases. This study also predicted the trends in the lung cancer burden caused by PMP, APMP, and HAP over the next 10 years, showing that the burden of lung cancer caused by PMP, APMP and HAP will increase by 2030.

Lung cancer shows a high incidence and mortality rate, making it one of the most common cancers globally [26]. Despite improvements in lifestyle and advancements in medical technology, including early screening, targeted therapy, and immunotherapy, lung cancer rates have decreased in developed countries in recent years [26]. In China, however, lung cancer remains the leading cancer in terms of both incidence and mortality, with rates continuing to rise each year [27]. It is well known that smoking is a major risk factor for lung cancer, accounting for 63.17% of total lung cancer deaths (75.46% in men and 36.65% in women) [28]. In addition to smoking, behavioral risks, environmental/occupational risks, air pollution, and PMP are also significant risk factors, with deaths caused by PMP ranking sixth [18]. Therefore, this risk factor should not be ignored in relation to lung cancer.

To date, many studies have observed a year‐on‐year increase in lung cancer mortality rates attributable to PM2.5 [29, 30, 31]. PMP can penetrate lung cells and even enter the bloodstream, posing a direct threat to health. PM2.5 is particularly dangerous because its diameter is smaller than what the human body's natural defense mechanisms can filter, allowing it to reach the alveoli, trigger inflammatory responses, and potentially lead to chronic airway and lung diseases, thereby increasing the risk of lung cancer.

This study examines the changes in lung cancer mortality and DALYs due to PMP from 1990 to 2021, highlighting that China has remained at the forefront of the global burden from PMP. The impact of PP (Particulate pollution) on lung cancer in China is a severe public health issue. Despite the Chinese government's efforts to control air pollution, such as implementing strict emission standards and shutting down heavily polluting factories [31], China's rapid economic growth and development over the past 30 years have led to a surge in energy demand and industrialization, resulting in severe air pollution issues [32]. In addition, it is important to note that the underreporting in low‐SDI regions may underestimate the true burden of lung cancer due to PMP, APMP, and HAP. Many countries in these regions face challenges related to limited cancer registries and incomplete data reporting, which could result in lower estimates of lung cancer burden. Future studies should aim to improve data collection in these areas, possibly through collaboration with local health organizations, to gain a more accurate understanding of the global burden.

Previous studies have reported on the relationship between household fine PMP and the burden of lung cancer through research on the GBD database. For example, one study investigated the burden of lung cancer from PM2.5 from 1990 to 2019 and concluded that due to the simultaneous decline in household PM2.5 exposure, the prevalence of lung cancer caused by household PM2.5 showed a downward trend from 1990 to 2019 [12]. Another study examined the burden of lung cancer caused by APMP from 1990 to 2019, concluding that due to increased exposure to PM2.5, population growth, and aging, the number of lung cancer deaths and disabilities related to ambient PM2.5 has substantially increased [29]. The difference between these studies and our research is that our study examines the impact of lung cancer burden caused by PMP, APMP, and HAP, which has broader applicability. Additionally, we used the APC model to describe the study. The advantage of the APC model is that it can analyze cohort effects and social change trends. Furthermore, this study also focuses on discussing the burden of lung cancer caused by PMP in China. Although the BAPC model provides statistically significant short‐term forecasts, long‐term projections remain sensitive to uncertainties in future environmental regulations, technological advancements, and demographic changes. These variables are challenging to predict and can significantly affect the trajectory of particulate pollution and related lung cancer burden. Further, the effectiveness of environmental policies, especially in emerging economies, could mitigate or exacerbate these projections. We recommend updating the BAPC model with more refined data as these variables evolve.

The APC model in this study shows a declining trend in lung cancer deaths due to PMP globally over time, similar to the trend observed for HAP. However, deaths from lung cancer caused by APMP are on the rise, indicating that as society progresses, APMP‐related lung cancer rates are also bound to increase. In recent years, there has been a significant improvement in indoor air quality, while outdoor pollution, such as PM2.5, has led to an increasing number of lung cancer cases. This could be because the improvement in indoor air quality has been more substantial with social development. Additionally, another study indicates that a reduction in the use of solid fuels in households, especially in rural areas, has led to decreased exposure to both environmental and indoor PM2.5 [33], thereby reducing the incidence of lung cancer. Simultaneously, the age model in this study also shows that the lung cancer mortality rates caused by PMP, APMP, and HAP are higher in men than in women. The possible reasons for this include the higher lung cancer burden among men, which may be due to occupational exposure and behavioral differences, with men being more likely to be exposed to PM2.5 [34]. It is crucial to consider sex‐specific behaviors and lifestyle factors when assessing the lung cancer burden attributable to ambient particulate exposure (APE). However, it should be noted that the relationship between PM2.5 exposure and lung cancer is complex and multifactorial. Other factors such as smoking and genetic susceptibility may also contribute to the differences in lung cancer DALYs between men and women [35, 36, 37]. The divergent trends in lung cancer mortality rates between men and women, especially the higher burden among men due to occupational exposure, suggest that targeted gender‐specific prevention strategies are needed. For example, interventions aimed at reducing exposure to industrial pollutants may benefit male populations more, while women, particularly in lower SDI countries, may benefit from policies targeting household air pollution and solid fuel use. This approach could help address the unique risks faced by each sex.

The primary sources of environmental PM2.5 are industry, power generation, and residential energy use [38]. Our research also shows that the global lung cancer burden caused by PM2.5 follows an inverted U‐shape with the increase in SDI. This phenomenon may be because of the initial rise in PM2.5 levels with societal development, followed by a decline as socio‐economic levels further improve and attention is given to environmental management [33, 39]. This finding is consistent with our study. The study also analyses global DALYs from lung cancer caused by PP, with Bosnia and Herzegovina, China, and Poland ranking the highest. Lung cancer due to PP is not only a personal health issue but also a socioeconomic burden. The cost of treating lung cancer is high, leading to workforce losses and increased pressure on medical resources, which has a negative impact on society as a whole. This study indicates that regardless of whether it is PMP, APMP, or HAP, the age group affected by lung cancer is shifting toward those > 50 years old. The exclusion of individuals under 50 years in the APC analysis may introduce limitations, including incomplete cohort representation (e.g., insufficient capture of environmental exposures in younger generations), reduced statistical power (obscuring early‐life risk trajectories), and potential identifiability biases (partial masking of age‐period‐cohort interactions). While this approach addresses data sparsity in younger age groups, it may compromise the precise assessment of long‐term risk accumulation and policy impacts. To address these limitations, future studies will integrate cross‐age data and develop hybrid models to enhance the resolution of cohort effects and temporal trends. With the intensification of aging in our country, PMP, APMP, and HAP are bound to cause an even greater lung cancer burden in the future.

The strength of this study lies in its comprehensive analysis of the lung cancer burden caused by PMP, APMP, and HAP; its use of the APC model to describe this burden; and its exploration of the relationship between SDI and the lung cancer burden caused by PMP, APMP, and HAP. The study also made predictions using the BAPC model. The limitations of the study are that the BAPC model's prediction is statistically significant only for the next decade, and this study, while global in scope, did not conduct specific studies on individual regions or countries. This study examined data on lung cancer caused by PMP, APMP, and HAP and analyzed their global prevalence trends. It explored the changes in mortality and DALYs caused by PMP, APMP, and HAP from 1990 to 2021 and used the APC model to analyze lung cancer caused by these factors. The study also investigated the relationship between the SDI ASMR, and ASDR of lung cancer caused by PMP, APMP, and HAP and applied the BAPC model to predict the mortality and DALYs owing to lung cancer caused by PMP, APMP, and HAP over the next decade. Compared to previous research [8], this study systematically examined the lung cancer burden caused by PMP and its subtypes—APMP and HAP—and used the BAPC model to predict the potential lung cancer burden caused by PMP, APMP, and HAP over the next decade.

While this study leverages the strengths of the GBD dataset in providing globally comparable estimates, it is essential to acknowledge its limitations. Differences in data quality, completeness, and comparability across countries may influence the accuracy of estimates. In some low‐SDI countries, limited cancer registries and underreporting may lead to underestimated burdens. These variations should be considered when interpreting regional disparities and long‐term trends.

Although the BAPC model provides statistically robust short‐term forecasts, long‐term projections inherently involve uncertainties due to assumptions regarding future environmental regulations, technological innovations, and demographic shifts, which may not always hold true. Socio‐economic factors, including poverty, limited healthcare accessibility, and poor nutritional status, can further mediate the relationship between particulate exposure and lung cancer risk, with disadvantaged populations facing higher exposures and fewer resources for mitigation. Incorporating these contextual variables in future analyses would enhance the policy relevance of burden estimates, though it may also obscure sudden and significant trend shifts in certain settings. Moreover, the effectiveness of Bayesian inference depends on the careful selection of prior distributions. Despite these limitations, the integration of second‐order stochastic excursion within BAPC improves the robustness and reliability of long‐term disease burden predictions [23].

To reduce the burden of PP on lung cancer, a comprehensive approach is needed. The government should strengthen environmental regulation and enforcement and intensify efforts against high‐pollution industries, and the public should actively participate in pollution reduction actions to boost environmental awareness, such as reducing vehicle use, supporting clean energy, and promoting green technologies. Given the significant impact of particulate pollution on lung cancer, the results of this study can serve as valuable evidence to support WHO air quality guidelines, particularly for high‐middle SDI countries undergoing rapid industrialization. These nations face a unique challenge where industrialization often outpaces the adoption of stringent environmental regulations. We suggest that policy makers in these regions prioritize the enforcement of air quality standards to reduce particulate pollution exposure and, consequently, lung cancer risk. Only through collective effort can the health impacts of PP be effectively reduced and the burden of lung cancer alleviated.

## 5 Conclusion

In the context of increased exposure to APMP, population growth, and aging, the global burden of particulate matter‐related lung cancer continues to grow, particularly among middle‐aged and older populations. The exposure to APMP changes with SDI, with middle‐ and high‐SDI countries bearing the heaviest lung cancer burden owing to high APMP exposure. By 2030, the lung cancer burden caused by PMP, APMP and HAP is projected to increase.

## Author Contributions

Concept: Yuhao Chen and Jun Chen. Design: Yuhao Chen, Hongbin Zhang and Xiuwen Zhang. Definition of Intellectual Content: Hongyu Liu. Literature Search: Xinyue Yang and Zixuan Hu. Data Acquisition: Xiuwen Zhang and Zhiqiang Zhang. Data Analysis: Yuhao Chen, Yongwen Li and Yaguang Fan. Statistical Analysis: Zixuan Hu. Manuscript Preparation: Yuhao Chen, Hongyu Liu and Yongwen Li. Manuscript Editing: Zixuan Hu. Manuscript Review: Xinyue Yang, Zhiqiang Zhang, Hongyu Liu and Yaguang Fan.

## Conflicts of Interest

The authors declare no conflicts of interest.

## Supporting information


**Data S1:** Supporting Information.

## Data Availability

The data that support the findings of this study are publicly available in the Global Burden of Disease (GBD) database (Institute for Health Metrics and Evaluation, IHME). The datasets analyzed in the current study can be accessed at the Global Health Data Exchange (GHDx) portal: https://ghdx.healthdata.org/. No additional datasets were generated during this study.
