# Supplementary material for: Effect of Particulate Matter Pollution on Global Lung Cancer Burden: A Systematic Analysis for the Global Burden of Disease Study 1990–2021
Source: Thorac Cancer. 2025 Nov 12;16(21):e70174. doi: 10.1111/1759-7714.70174 (PMC12612555; doi:10.1111/1759-7714.70174)
Supplement: Supplementary file 1 — Data S1: Supporting Information. [file TCA-16-e70174-s001.pdf]

## Supplementary Tables

Supplement table: 1 Global burden of lung cancer attributable to PMP in 1990 and 2021, and the temporal trends from 1990 to 2021

| Location | ASMR in 1990, per 100,000 | ASMR in 2021, per 100,00 | ASDR in 1990, per 100,000 | ASDR in 2021, per 100,000 |
|---|---|---|---|---|
| China | 13.6 (9.2 to 18) | 10.1 (6.3 to 14.4) | 336.3 (227.5 to 446.2) | 226.5 (141.6 to 320.6) |
| Democratic People's Republic of Korea | 10.4 (6.3 to 15.2) | 9.8 (5.3 to 15.5) | 269.7 (159.7 to 392.9) | 255.7 (140 to 404) |
| Indonesia | 5.7 (3.5 to 7.9) | 4.2 (2.2 to 6.7) | 144.8 (89.9 to 200.7) | 100.4 (52.8 to 160) |
| Taiwan (Province of China) | 4.9 (2 to 8.9) | 3.4 (2.1 to 4.9) | 119.5 (49.3 to 216.3) | 75.6 (46.9 to 107.9) |
| Cambodia | 10.3 (6.5 to 15.1) | 8.9 (5.4 to 13.3) | 260.6 (164.3 to 381.3) | 213.2 (129.1 to 315.5) |
| Myanmar | 9.8 (5.9 to 15) | 6.5 (3.8 to 9.7) | 250 (152.1 to 384.9) | 155.8 (90.6 to 234.4) |
| Malaysia | 3.4 (1.5 to 5.9) | 2.4 (1.4 to 3.6) | 84.6 (36.4 to 145.7) | 56.4 (32.3 to 86.5) |
| Sri Lanka | 3.4 (2.3 to 4.7) | 1.7 (0.8 to 3.2) | 85.1 (55.9 to 116.3) | 42.3 (19.3 to 82.4) |
| Lao People's Democratic Republic | 10.2 (5.9 to 17) | 7.4 (4.1 to 11.3) | 263.2 (151.5 to 439.8) | 177.7 (97.8 to 275) |
| Maldives | 5.1 (3 to 7.8) | 0.8 (0.4 to 1.3) | 119.2 (68.5 to 182.1) | 16.1 (7.9 to 27.3) |
| Philippines | 6.3 (4 to 8.6) | 4.2 (2.6 to 6.1) | 152.8 (98 to 208.1) | 103.4 (63.5 to 152.1) |
| Thailand | 11.3 (7.2 to 16) | 5.3 (3.2 to 8.1) | 273.6 (173.9 to 390.5) | 125.8 (74.5 to 192.1) |
| Timor-Leste | 5.3 (3.1 to 7.9) | 4.6 (2.5 to 6.8) | 130.2 (76.4 to 191.7) | 109.8 (60.6 to 164.6) |
| Fiji | 2.8 (1.5 to 4.4) | 1.4 (0.4 to 2.7) | 69.4 (37.9 to 109.4) | 33.8 (10 to 64.8) |
| Viet Nam | 8.9 (5.8 to 12.3) | 5.1 (3 to 7.7) | 232.5 (150.5 to 327.6) | 131.5 (76.2 to 201.1) |
| Marshall Islands | 5.4 (2.8 to 9) | 5.3 (2.7 to 9.1) | 133.1 (67.8 to 224.5) | 130.2 (65.1 to 225) |
| Kiribati | 5.8 (3.7 to 8) | 4.5 (2.5 to 7.1) | 146.1 (93.9 to 202.4) | 112.9 (60.8 to 179.4) |
| Papua New Guinea | 6.4 (3.5 to 11.8) | 6.8 (3.6 to 11) | 155.5 (84.6 to 287.2) | 159.8 (85.1 to 259.2) |
| Micronesia (Federated States of) | 8.7 (5 to 13) | 5.9 (3 to 10.1) | 217.4 (126.5 to 332.6) | 148 (73.5 to 255.1) |
| Vanuatu | 7.6 (4.2 to 13.6) | 6.8 (4 to 11.4) | 183.1 (99.3 to 327.7) | 164.8 (95.6 to 276.7) |
| Solomon Islands | 8.2 (4.8 to 12.9) | 8 (5 to 11.8) | 213.5 (116.3 to 346.6) | 213.2 (129.2 to 320.5) |
| Samoa | 3 (1.8 to 4.2) | 2.3 (1.2 to 3.5) | 73.2 (44.8 to 105.1) | 56.7 (30.8 to 88.6) |
| Tonga | 9.1 (5.7 to 13.3) | 5.9 (3.2 to 9.3) | 212.8 (132.8 to 311.9) | 137.9 (73.1 to 220.6) |

| | | | | |
|---|---|---|---|---|
| Azerbaijan | 4.7 (2 to 7.4) | 2.4 (1.1 to 4.2) | 136.1 (56.7 to 216.9) | 63.4 (29 to 112.9) |
| Kazakhstan | 6.9 (2.7 to 11.7) | 2.3 (1.4 to 3.5) | 198.5 (77.5 to 337.2) | 61.6 (36.2 to 92.3) |
| Armenia | 7.9 (4 to 12.4) | 6 (3.6 to 8.7) | 226.5 (114.3 to 353.6) | 147.6 (88.7 to 213.5) |
| Mongolia | 10.9 (6.4 to 16.1) | 5.6 (3 to 8.9) | 277 (164.1 to 409) | 138.9 (75.7 to 221.5) |
| Georgia | 4.6 (1.9 to 7.8) | 3.9 (2.2 to 6.2) | 133.7 (54.7 to 226.6) | 103.3 (56.8 to 164.6) |
| Kyrgyzstan | 6.9 (4.1 to 10) | 2.7 (1.6 to 3.9) | 200.4 (117.9 to 289.3) | 70.8 (42.1 to 101.3) |
| Tajikistan | 5.8 (3.5 to 8.2) | 2.4 (1.4 to 3.9) | 162.6 (98.5 to 229.9) | 64.6 (36.4 to 104.5) |
| Uzbekistan | 4.3 (2.5 to 6.4) | 1.7 (1 to 2.4) | 123.5 (70.9 to 183.8) | 44.6 (26.4 to 64.2) |
| Turkmenistan | 2.2 (0.6 to 4.2) | 1.2 (0.6 to 2) | 62.6 (17.5 to 121) | 34.8 (17.5 to 58) |
| Albania | 9.3 (5.8 to 13.5) | 3.5 (1.8 to 6) | 230.6 (144.1 to 331.4) | 81.9 (41.2 to 142.9) |
| Bulgaria | 6.1 (3.3 to 9.5) | 4.6 (2.7 to 7.2) | 175.9 (95 to 275.1) | 123.7 (72.8 to 194.1) |
| Czechia | 8.4 (4.2 to 13.9) | 2.8 (1.7 to 4.1) | 228.2 (113.4 to 377) | 64.6 (39.1 to 94.7) |
| Croatia | 7.8 (3.8 to 12.8) | 3.9 (2.4 to 5.7) | 206.4 (101.6 to 339.9) | 96.1 (58.2 to 141.2) |
| Bosnia and Herzegovina | 13.3 (8.7 to 17.8) | 8 (4.7 to 12.2) | 358.4 (237.3 to 480.8) | 199.7 (115 to 304) |
| North Macedonia | 9 (5.7 to 12.9) | 6.3 (3.6 to 9.8) | 242 (154.4 to 347.2) | 158.1 (91.3 to 247.6) |
| Hungary | 9.6 (4.8 to 15.3) | 4.8 (2.8 to 7.5) | 266.9 (133.3 to 427.4) | 123 (71.8 to 191.5) |
| Poland | 11 (6.4 to 16.3) | 5.7 (3.5 to 8.2) | 302.2 (175.2 to 451.3) | 134.7 (82.8 to 192.2) |
| Serbia | 11.2 (6.1 to 17.2) | 7.4 (4.3 to 11.6) | 300 (165.6 to 460.8) | 193.5 (110.9 to 302.1) |
| Montenegro | 10.6 (5.5 to 16.9) | 7.5 (4.1 to 13.8) | 282.8 (146.4 to 455) | 185.3 (100.9 to 336.4) |
| Romania | 5.3 (2.8 to 8.4) | 3.5 (2 to 5.2) | 160.6 (85.2 to 256.1) | 93.4 (53.3 to 138.9) |
| Slovakia | 7.6 (3.7 to 12.2) | 3 (1.8 to 4.6) | 208.1 (99.7 to 335.3) | 74.2 (44.5 to 115.2) |
| Estonia | 5 (1.9 to 8.9) | 0.8 (0.2 to 1.6) | 137.3 (53.1 to 245.8) | 18.4 (5.3 to 38.3) |
| Slovenia | 6.5 (3.3 to 10.7) | 2.8 (1.6 to 4.1) | 177.5 (88 to 291.7) | 64.2 (37 to 95.7) |
| Lithuania | 5.7 (2.7 to 9.4) | 1.5 (0.8 to 2.4) | 156.4 (74 to 258.8) | 38.6 (19.9 to 60.1) |
| Russian Federation | 6.2 (2.7 to 10.2) | 1.7 (0.9 to 2.8) | 176.1 (75.9 to 291.7) | 44.1 (22.4 to 73) |
| Belarus | 6.9 (3.6 to 10.8) | 2.4 (1.3 to 3.6) | 198 (102.3 to 310.8) | 64.5 (35.4 to 97.6) |
| Latvia | 7.4 (3.7 to 11.8) | 2.1 (1.2 to 3.2) | 206 (103.8 to 329.9) | 51.6 (28.9 to 79.9) |
| Republic of Moldova | 9.5 (5.8 to 13.3) | 2.4 (1.3 to 3.8) | 266.4 (162.2 to 373.5) | 66.3 (36.5 to 103.9) |
| Brunei Darussalam | 1.9 (0.1 to 4.7) | 1.1 (0.2 to 2.4) | 41.4 (2.7 to 104.8) | 23.8 (4.7 to 50.3) |

| | | | | |
|---|---|---|---|---|
| Ukraine | 8.1 (4.1 to 13.4) | 2 (1 to 3.7) | 232.5 (118.1 to 384.9) | 55.9 (27 to 103.2) |
| Republic of Korea | 4.9 (2 to 8.7) | 4.4 (2.5 to 6.7) | 121.7 (50.8 to 216.9) | 85.7 (48.3 to 130.8) |
| Japan | 2.3 (0.4 to 4.9) | 1.9 (0.9 to 3.1) | 49.8 (9.1 to 107.5) | 37.2 (17.8 to 61.3) |
| Singapore | 7.5 (2.6 to 13) | 2 (1 to 3.3) | 171.9 (60.8 to 297.8) | 40 (19.9 to 66.5) |
| Australia | 1.3 (0 to 3.7) | 1.2 (0.6 to 2) | 31.8 (1 to 89.6) | 26.7 (13.3 to 42.9) |
| Austria | 4.9 (2.3 to 7.9) | 1.8 (1 to 2.6) | 122.5 (56.9 to 199.4) | 41.1 (23.6 to 59.4) |
| Cyprus | 3.4 (1.4 to 6) | 2.1 (1.2 to 3.2) | 73.9 (30.7 to 130.8) | 47.6 (27 to 70.8) |
| New Zealand | 1.2 (0 to 3.8) | 0.8 (0.3 to 1.5) | 29.9 (0.7 to 91.4) | 19 (6.8 to 34) |
| Belgium | 8.2 (3.7 to 13.4) | 2.3 (1.3 to 3.4) | 203 (91.1 to 332.4) | 52.4 (29.8 to 78.7) |
| Andorra | 5.2 (1.7 to 9.9) | 1.2 (0.5 to 2.2) | 127 (42.3 to 241.4) | 29 (11.6 to 51.7) |
| Finland | 1.6 (0.2 to 3.5) | 0.3 (0 to 0.7) | 38.3 (5.7 to 85) | 6.9 (0.9 to 16.1) |
| Germany | 5.3 (2.4 to 8.8) | 1.8 (1 to 2.8) | 137.7 (61.8 to 227.7) | 43.4 (24.5 to 66.3) |
| Iceland | 1.3 (0.1 to 3.1) | 0.4 (0.1 to 1) | 31 (2.4 to 75.2) | 9.7 (1.7 to 22.8) |
| Denmark | 6.7 (2.6 to 11.8) | 2 (1 to 3.2) | 169.8 (66.7 to 297.4) | 42.8 (22.1 to 68.5) |
| France | 4.2 (1.7 to 7.4) | 1.7 (1 to 2.7) | 112.4 (46.5 to 195.4) | 44.1 (24.7 to 67.5) |
| Greece | 7.1 (3.2 to 11.5) | 3.9 (2.4 to 5.5) | 170.5 (77.3 to 275.1) | 91.2 (56.3 to 130.1) |
| Ireland | 4 (1.3 to 7.4) | 1 (0.4 to 1.7) | 94.4 (31.9 to 175.9) | 22.1 (9.7 to 36.7) |
| Israel | 4.2 (2 to 6.8) | 2.7 (1.7 to 3.7) | 102.2 (49.9 to 163.1) | 59.4 (36.8 to 84) |
| Italy | 7.2 (3.6 to 11) | 2.5 (1.5 to 3.6) | 181.6 (92.3 to 279.9) | 54.8 (32.9 to 78.3) |
| Malta | 3.6 (1.4 to 6.4) | 1.6 (1 to 2.4) | 87.1 (35.1 to 152.2) | 38.9 (22.7 to 56.6) |
| Norway | 2 (0.6 to 3.9) | 0.6 (0.2 to 1.2) | 49.2 (13.8 to 95.7) | 12.9 (4.5 to 24.9) |
| Luxembourg | 5.3 (2.1 to 9.4) | 1.2 (0.6 to 2) | 132.2 (52.4 to 234.8) | 28.3 (13.6 to 45.6) |
| Netherlands | 7.5 (3.4 to 12.4) | 2.4 (1.4 to 3.6) | 182.2 (82.9 to 300.1) | 54 (31.4 to 80.7) |
| Spain | 3.6 (1.4 to 6.4) | 1.4 (0.7 to 2.2) | 94.2 (36.5 to 164.9) | 33.5 (17.6 to 52.8) |
| Portugal | 2 (0.7 to 3.8) | 0.8 (0.4 to 1.4) | 51.9 (17 to 96.1) | 20.6 (9.6 to 34.6) |
| Switzerland | 3.9 (1.6 to 6.7) | 1.1 (0.6 to 1.8) | 98.7 (40.7 to 169.7) | 25.5 (13.6 to 40) |
| Sweden | 1.5 (0.4 to 2.9) | 0.4 (0.1 to 0.8) | 35.6 (8.8 to 71.8) | 8 (2.2 to 16.1) |
| United Kingdom | 6.2 (2.5 to 10.7) | 1.7 (0.9 to 2.6) | 144.7 (59.4 to 250.7) | 34.9 (19.1 to 53.2) |
| Chile | 4.5 (2.6 to 6.7) | 2.4 (1.3 to 3.6) | 110.9 (66.1 to 166.5) | 53.8 (29.5 to 79.9) |

| | | | | |
|---|---|---|---|---|
| Argentina | 4.5 (2 to 7.5) | 2.1 (1 to 3.5) | 119.2 (53 to 200.8) | 51 (22.9 to 83.7) |
| Uruguay | 4.5 (1.6 to 8) | 2.2 (0.8 to 4.2) | 118.6 (41.2 to 212.3) | 53.9 (20.1 to 101.7) |
| United States of America | 4.9 (1.9 to 8.8) | 1 (0.4 to 1.7) | 123.1 (47.8 to 222.2) | 21.5 (9.2 to 37.1) |
| Canada | 3.1 (0.8 to 6.2) | 0.7 (0.2 to 1.3) | 78 (20.5 to 152.5) | 14.7 (4.9 to 28.2) |
| Antigua and Barbuda | 1.4 (0.3 to 3.1) | 1.2 (0.5 to 2.3) | 34.8 (7.6 to 75.8) | 28.3 (11.2 to 52.7) |
| Bahamas | 2.2 (0.4 to 5) | 1.8 (0.6 to 3.6) | 57.9 (10 to 133) | 45.5 (15.9 to 91.1) |
| Barbados | 1.6 (0.4 to 3.3) | 1.5 (0.6 to 2.6) | 38.6 (9 to 79.9) | 34.2 (13.8 to 60) |
| Belize | 2.5 (1.3 to 4) | 2.3 (1.1 to 3.8) | 60.4 (30.7 to 98.2) | 55.9 (27 to 93.4) |
| Cuba | 5.2 (1.7 to 10) | 4.7 (2.1 to 7.7) | 121.6 (40 to 234.5) | 107.6 (47.7 to 178.1) |
| Dominica | 4.3 (2.3 to 6.8) | 2.8 (1.2 to 5.1) | 101.6 (54.6 to 159.3) | 67.6 (28.4 to 121.8) |
| Dominican Republic | 2.9 (1.7 to 4.4) | 2 (0.6 to 3.9) | 70.5 (41.6 to 106.3) | 49.6 (16.1 to 96.3) |
| Grenada | 2.9 (1.4 to 4.8) | 1.9 (0.8 to 3.6) | 74.4 (34.5 to 123.1) | 46.9 (19 to 86.1) |
| Guyana | 1.7 (0.8 to 2.9) | 1.4 (0.6 to 2.5) | 43.6 (20.8 to 73.9) | 36.4 (16.3 to 64.6) |
| Haiti | 5.7 (3.3 to 9) | 4.5 (2.5 to 7.5) | 145.2 (84.7 to 229.9) | 111.7 (59.8 to 184) |
| Jamaica | 4.1 (2.3 to 6.1) | 2.4 (1.2 to 3.9) | 105.7 (59.5 to 157.1) | 57.5 (28.8 to 95.7) |
| Saint Lucia | 3.2 (1.5 to 5.3) | 1.8 (0.8 to 3.3) | 76.2 (36.5 to 127) | 43.6 (18.1 to 78.2) |
| Saint Vincent and the Grenadines | 2.3 (1.1 to 3.9) | 1.6 (0.6 to 2.9) | 55.4 (27.9 to 94.8) | 38.3 (15.4 to 71.9) |
| Suriname | 3.3 (1.5 to 5.6) | 2.7 (1.2 to 4.7) | 82.5 (37.6 to 140.6) | 69.5 (30.3 to 119.1) |
| Trinidad and Tobago | 1.9 (0.4 to 4) | 1.7 (0.6 to 3.3) | 47.1 (8.8 to 99.9) | 43.5 (15.6 to 84.4) |
| Bolivia (Plurinational State of) | 6 (3.6 to 8.7) | 3 (1.6 to 4.9) | 144.4 (85.7 to 210.1) | 66.5 (35.8 to 110.9) |
| Ecuador | 2.8 (1.7 to 4) | 1.2 (0.6 to 1.9) | 67.1 (39.8 to 95) | 25.2 (13.2 to 42.3) |
| Peru | 5.6 (3.6 to 7.6) | 2.4 (1.4 to 3.7) | 133.6 (85.5 to 183) | 55 (31.1 to 85.7) |
| Colombia | 3.9 (2.2 to 5.6) | 1.2 (0.7 to 1.9) | 93.7 (54.1 to 135.2) | 27.6 (14.8 to 43) |
| Costa Rica | 2.4 (1.3 to 3.7) | 0.8 (0.4 to 1.2) | 55.6 (29.1 to 85.2) | 17.9 (9.9 to 27.3) |
| El Salvador | 3 (1.9 to 4.1) | 1.5 (0.8 to 2.4) | 73.7 (46.9 to 100.5) | 36.8 (18.7 to 58.6) |
| Guatemala | 3.3 (2.2 to 4.4) | 1.6 (0.9 to 2.3) | 77.7 (51.5 to 103.3) | 36.4 (21.4 to 54.8) |
| Mexico | 3.3 (1.9 to 5.1) | 0.8 (0.5 to 1.3) | 74.5 (42.7 to 112.8) | 19.1 (10.7 to 29.8) |
| Honduras | 4.7 (3 to 6.8) | 5.5 (3 to 8.9) | 118 (74.8 to 166.7) | 129.8 (68.6 to 207.9) |
| Panama | 2.8 (1.5 to 4.3) | 0.7 (0.3 to 1.2) | 66.4 (36.1 to 102.5) | 15.9 (7.4 to 27.6) |

| | | | | |
|---|---|---|---|---|
| Nicaragua | 2.1 (1.4 to 2.9) | 1.3 (0.7 to 1.9) | 50.1 (32.5 to 70) | 29.8 (16.8 to 45.5) |
| Venezuela (Bolivarian Republic of) | 3.1 (1.4 to 5.1) | 1.9 (0.9 to 3.3) | 78.7 (36 to 129.8) | 45.4 (22.8 to 78.9) |
| Brazil | 2.9 (1.4 to 4.8) | 1.4 (0.7 to 2.2) | 71.1 (35.3 to 120.2) | 33.5 (16.8 to 52.5) |
| Paraguay | 2.7 (1.5 to 4.1) | 2 (0.6 to 4) | 66.5 (36.9 to 103.5) | 45.9 (12.9 to 93.8) |
| Algeria | 1.4 (0.8 to 2.2) | 1.2 (0.7 to 1.9) | 33 (19.2 to 51.1) | 27.6 (15.8 to 42.6) |
| Egypt | 1.8 (1.2 to 2.5) | 4.6 (2.8 to 6.7) | 49.5 (31.7 to 67.9) | 112.5 (68.9 to 163.5) |
| Bahrain | 12.6 (8.2 to 16.9) | 6.5 (4 to 9.5) | 279.9 (181.3 to 375.5) | 135.2 (81.9 to 197.7) |
| Iran (Islamic Republic of) | 2.4 (1.5 to 3.5) | 2.3 (1.5 to 3.2) | 58.8 (36.3 to 84.1) | 53.8 (33.6 to 73.4) |
| Iraq | 4.1 (2.4 to 6.1) | 4.6 (2.7 to 6.8) | 104.4 (60 to 154.8) | 104.5 (61.8 to 158.3) |
| Jordan | 2.3 (1.4 to 3.3) | 2.3 (1.3 to 3.5) | 58.3 (35.1 to 86.2) | 54.9 (30.8 to 84.6) |
| Kuwait | 4.3 (2.9 to 5.9) | 2.4 (1.4 to 3.4) | 105 (69.1 to 141.3) | 52.1 (31.7 to 74.7) |
| Libya | 5.2 (2.9 to 7.9) | 5.8 (3 to 9.4) | 126.1 (70.6 to 191.3) | 139.8 (71.8 to 226.3) |
| Lebanon | 3.3 (1.7 to 5) | 3.5 (1.9 to 5.8) | 79 (41.1 to 122.8) | 80.4 (43.9 to 132.2) |
| Palestine | 4.1 (2.3 to 6.5) | 3.9 (2.2 to 5.9) | 97.6 (52.8 to 155.7) | 90.1 (51.8 to 138.9) |
| Morocco | 2.6 (1.6 to 3.7) | 2.2 (1.3 to 3.4) | 64.7 (39.3 to 92.4) | 53.3 (30.6 to 81.8) |
| Oman | 1.7 (1 to 2.7) | 1.2 (0.7 to 1.9) | 42.9 (24.2 to 67.3) | 28.1 (16 to 44) |
| Qatar | 9.2 (5.8 to 12.6) | 4.5 (2.6 to 6.9) | 207.3 (130.2 to 283.5) | 96.9 (55.1 to 150.5) |
| Saudi Arabia | 1.8 (1 to 2.6) | 2 (1.2 to 2.9) | 43.6 (25 to 64.9) | 47.6 (28.2 to 69.4) |
| Syrian Arab Republic | 2.5 (1.4 to 3.8) | 2.4 (1.2 to 4) | 63.4 (35.2 to 98.8) | 57 (29 to 97.2) |
| Tunisia | 3.6 (2.1 to 5.4) | 3.1 (1.6 to 4.9) | 87.9 (51.3 to 129.9) | 76.2 (38.9 to 124.7) |
| Turkey | 9.1 (5.3 to 13.5) | 5.9 (3.5 to 9) | 240 (140.1 to 353.9) | 144.8 (86.8 to 220.9) |
| United Arab Emirates | 5.2 (3 to 7.8) | 3.6 (2.1 to 5.5) | 129.3 (73.9 to 195.7) | 73.7 (41.5 to 114.3) |
| Yemen | 3.3 (1.7 to 5.9) | 2.9 (1.5 to 5.2) | 86 (43.7 to 155.5) | 71.8 (36.7 to 127.4) |
| Afghanistan | 4 (2 to 7.8) | 4.5 (2.6 to 6.8) | 104 (50.3 to 197.5) | 113.1 (66.3 to 172) |
| Bangladesh | 3.6 (2.3 to 5.5) | 2.3 (1.3 to 3.5) | 96.3 (61.5 to 146.2) | 58.2 (32.6 to 89.3) |
| Bhutan | 1.9 (1 to 3) | 1.3 (0.7 to 2.1) | 49.4 (26.3 to 80.4) | 31 (16.1 to 49.8) |
| Nepal | 2.4 (1.4 to 3.8) | 2.2 (1.3 to 3.5) | 64.1 (37.1 to 99.7) | 55.3 (30.9 to 85.1) |
| India | 2.1 (1.3 to 2.8) | 2 (1.3 to 2.9) | 56 (36.6 to 74.3) | 53.8 (34.8 to 74.9) |
| Pakistan | 5 (3.1 to 6.7) | 4.8 (2.8 to 7.2) | 125.3 (77.6 to 169.2) | 119.8 (71.5 to 180.9) |

| | | | | |
|---|---|---|---|---|
| Central African Republic | 5.1 (2.9 to 9.7) | 4.4 (2.4 to 8.4) | 137.2 (78.1 to 261.5) | 118.6 (63.3 to 228.1) |
| Angola | 4.7 (2.8 to 7) | 3 (1.6 to 4.6) | 121.8 (72.6 to 184.5) | 75.1 (40.4 to 117.3) |
| Congo | 5.6 (3.2 to 8.1) | 4 (2.3 to 6) | 147 (83.5 to 213.3) | 99.7 (57.3 to 153.4) |
| Democratic Republic of the Congo | 3.6 (2.1 to 6.4) | 3.3 (1.7 to 6.1) | 91.8 (52.8 to 161) | 84.3 (43.1 to 156) |
| Equatorial Guinea | 4.7 (2.6 to 8.2) | 3.1 (1.4 to 5.5) | 125.9 (69.7 to 221.1) | 75.6 (33.9 to 134) |
| Gabon | 3.4 (1.6 to 5.8) | 3.1 (1.5 to 5) | 86.1 (40.2 to 151) | 77.4 (36.4 to 125.4) |
| Burundi | 3.3 (2.1 to 4.9) | 2.6 (1.5 to 4) | 86.3 (54.4 to 128.4) | 64.2 (36 to 99.9) |
| Djibouti | 2.6 (1.5 to 4) | 2.9 (1.5 to 4.9) | 66.7 (37.5 to 101.1) | 71.4 (36.3 to 120.9) |
| Comoros | 2.8 (1.7 to 4.1) | 2.6 (1.5 to 4) | 72.1 (43.6 to 104.2) | 64.4 (36.1 to 97.4) |
| Ethiopia | 5.1 (3.2 to 7.7) | 3 (1.9 to 4.2) | 135.1 (81.3 to 206.8) | 70.8 (45.8 to 98.8) |
| Eritrea | 3.1 (2 to 4.5) | 3.1 (2 to 4.6) | 84.4 (53.6 to 124.8) | 82.2 (49 to 123.4) |
| Madagascar | 2.6 (1.8 to 3.5) | 2.3 (1.4 to 3.2) | 66.8 (45.5 to 90.3) | 57 (35.3 to 82.6) |
| Kenya | 0.8 (0.4 to 1.3) | 1.2 (0.8 to 1.8) | 20.1 (10.9 to 31.6) | 29.6 (18.7 to 43.2) |
| Malawi | 0.9 (0.6 to 1.3) | 1.1 (0.7 to 1.6) | 23.6 (15.1 to 32.1) | 27.7 (17 to 42.5) |
| Mozambique | 2.4 (1.6 to 3.2) | 2.9 (1.8 to 4.1) | 53.8 (36.9 to 73.1) | 67.9 (40.4 to 95.7) |
| Mauritius | 1.1 (0.5 to 1.9) | 0.6 (0.2 to 1.1) | 25.9 (11.1 to 44.8) | 13.8 (4.3 to 26.1) |
| Seychelles | 1.1 (0.3 to 2.1) | 0.8 (0.2 to 1.7) | 28.1 (7.5 to 52.4) | 19.4 (5.9 to 39.7) |
| Rwanda | 3.8 (2.5 to 5.3) | 3.4 (2 to 5.2) | 100.7 (64.1 to 138.2) | 83.7 (47.6 to 129.1) |
| United Republic of Tanzania | 2.8 (1.7 to 3.9) | 2.8 (1.7 to 4.1) | 70.1 (43.1 to 99.9) | 68.3 (40.7 to 102.7) |
| Somalia | 2.5 (1.4 to 4.4) | 2.2 (1.1 to 4.2) | 67.2 (36.1 to 118.2) | 58.3 (29.4 to 115.5) |
| Zambia | 3 (2 to 4.2) | 3.8 (2.2 to 6.3) | 77.4 (50.5 to 106) | 97.4 (55.5 to 168.9) |
| Uganda | 2.6 (1.7 to 3.6) | 2.8 (1.7 to 4.2) | 64 (41.3 to 89.7) | 69.1 (40 to 102.5) |
| Botswana | 5.2 (3 to 7.9) | 2.6 (1.3 to 4.7) | 134.2 (76.1 to 207.8) | 63.2 (31.3 to 115.3) |
| Namibia | 1.7 (1 to 2.5) | 1.2 (0.6 to 2.2) | 44.2 (25.1 to 64.6) | 32 (15.4 to 58.1) |
| Lesotho | 4.1 (2.4 to 6.3) | 7 (3.8 to 10.9) | 104.8 (61.9 to 164.8) | 190.1 (102.8 to 304.7) |
| Eswatini | 5.8 (2.9 to 9.8) | 5 (2.3 to 8.6) | 151.2 (74.8 to 259.1) | 133.2 (61.7 to 229.4) |
| South Africa | 3.8 (2.3 to 5.7) | 3.1 (1.8 to 4.5) | 104.7 (63.9 to 154.4) | 78.1 (46.1 to 116.7) |
| Zimbabwe | 4.4 (2.8 to 6.1) | 4.8 (2.9 to 7.1) | 106.7 (67.9 to 148.8) | 122.5 (74.7 to 182.1) |
| Benin | 2.7 (1.8 to 3.7) | 2.6 (1.6 to 3.8) | 66 (44.5 to 89.6) | 61.6 (35.9 to 91) |

| | | | | |
|---|---|---|---|---|
| Cameroon | 2.9 (2 to 4) | 3.1 (1.9 to 4.7) | 70.4 (47.3 to 98.1) | 74.3 (44.3 to 114.9) |
| Burkina Faso | 2.3 (1.5 to 3.6) | 2.5 (1.5 to 3.9) | 57.1 (36.1 to 87) | 59.2 (34.8 to 92.2) |
| Chad | 2.1 (1.3 to 3.2) | 3 (1.9 to 4.9) | 49.7 (30 to 76.6) | 71.7 (43.9 to 116) |
| Cabo Verde | 4.3 (2.7 to 5.9) | 5 (2.9 to 7.3) | 101.4 (63.6 to 139.7) | 109.3 (63.2 to 161.3) |
| Gambia | 1.2 (0.8 to 1.8) | 1.3 (0.8 to 2) | 31.4 (20.2 to 45.8) | 32.8 (19.3 to 49.7) |
| Coted'Ivoire | 1.7 (1.1 to 2.3) | 1.5 (0.9 to 2.4) | 40.7 (26.1 to 56.1) | 37.1 (22.2 to 59.4) |
| Guinea | 2.7 (1.8 to 3.8) | 3.4 (2 to 5.3) | 67.1 (42.8 to 93.7) | 83.1 (48.9 to 130.5) |
| Ghana | 2.2 (1.4 to 3.1) | 2.3 (1.3 to 3.3) | 51.6 (33.4 to 73.2) | 50.6 (29.6 to 74.7) |
| Liberia | 2.5 (1.6 to 3.6) | 2.4 (1.3 to 3.9) | 59.1 (37.5 to 85.4) | 55.8 (30.5 to 92.3) |
| Guinea-Bissau | 3.7 (2.3 to 5.5) | 3.7 (2.1 to 5.2) | 94.7 (58 to 141.5) | 90.5 (52.1 to 132.4) |
| Mauritania | 3 (1.9 to 4.2) | 2.8 (1.7 to 4.3) | 73.1 (46.1 to 102.7) | 64 (38.9 to 97.7) |
| Mali | 2 (1.3 to 2.7) | 2.1 (1.3 to 3) | 49 (33.4 to 66.5) | 49.7 (29.8 to 72.8) |
| Nigeria | 0.5 (0.3 to 0.8) | 0.5 (0.3 to 0.7) | 13.1 (8 to 19.3) | 12.1 (7.6 to 17.4) |
| Niger | 2 (1.2 to 3.1) | 2 (1.2 to 3.5) | 47.6 (29 to 76.7) | 46.4 (26.7 to 82.7) |
| Sao Tome and Principe | 5.3 (3.3 to 7.2) | 4.9 (2.8 to 7.5) | 123.5 (76.4 to 170.2) | 109.9 (62.8 to 169) |
| Sierra Leone | 2.7 (1.6 to 4) | 2.7 (1.5 to 4.1) | 64.3 (38.2 to 95.5) | 64.2 (35.9 to 98.2) |
| Senegal | 2.9 (1.8 to 4.2) | 3.6 (2.2 to 5.3) | 71.7 (45 to 100.6) | 83.8 (51.4 to 124.9) |
| Bermuda | 2.1 (0 to 5.6) | 0.8 (0.1 to 1.5) | 49.6 (0 to 132.5) | 17.1 (3.3 to 34.7) |
| Togo | 2.9 (1.8 to 4.2) | 3.3 (1.9 to 5) | 71.5 (43 to 102) | 79 (44.6 to 122.9) |
| American Samoa | 1.1 (0 to 4) | 1.2 (0.1 to 2.8) | 27 (0 to 95.1) | 27.2 (2.6 to 65.2) |
| Cook Islands | 1.8 (0.1 to 5.2) | 0.8 (0 to 2.2) | 41.1 (1.3 to 120.6) | 18.7 (0 to 48.7) |
| Guam | 2 (0 to 5.5) | 1.6 (0.7 to 2.6) | 45.4 (0 to 126) | 41.4 (18.9 to 69.5) |
| Greenland | 4 (0 to 11.5) | 1.8 (0.1 to 5) | 101.2 (0.4 to 291.7) | 42.2 (1.4 to 116.3) |
| Nauru | 1.4 (0 to 5.4) | 1.3 (0.1 to 3.1) | 33.6 (0 to 132.5) | 32.9 (3.4 to 79.4) |
| Monaco | 3.4 (0.6 to 7.3) | 3.8 (1.7 to 6.6) | 84.2 (13.7 to 179.7) | 89.7 (40 to 155.8) |
| Northern Mariana Islands | 2.8 (0 to 7.9) | 2.4 (1.1 to 4.1) | 67 (0 to 188.8) | 55.5 (24.6 to 94.6) |
| Niue | 3 (1.1 to 5.7) | 1.1 (0.1 to 2.5) | 72.5 (25.2 to 135.3) | 25.2 (2.4 to 59.7) |
| Puerto Rico | 0.5 (0 to 1.8) | 0.3 (0.1 to 0.5) | 12.7 (0 to 42) | 6.3 (1.5 to 12.3) |
| Palau | 2 (0 to 5.7) | 1.9 (0.2 to 4) | 46.5 (0.2 to 136.3) | 42.8 (4.2 to 91.8) |

| | | | | |
|---|---|---|---|---|
| San Marino | 3.9 (1.3 to 7.6) | 1.2 (0.5 to 2.2) | 92.1 (30.9 to 178.7) | 27.8 (10.8 to 53.2) |
| Saint Kitts and Nevis | 0.8 (0.2 to 1.8) | 0.4 (0.1 to 0.7) | 20 (4.8 to 43.3) | 9.7 (3.2 to 17.2) |
| Tuvalu | 6.5 (3.8 to 10.9) | 1.9 (0.8 to 3.5) | 162.9 (93.2 to 274.6) | 46.8 (20.5 to 86.5) |
| Tokelau | 0.8 (0 to 2.5) | 0.6 (0 to 1.7) | 17.7 (0.6 to 59) | 14.9 (0.1 to 39.8) |
| United States Virgin Islands | 0.8 (0 to 2.2) | 0.5 (0.2 to 1) | 19.9 (0.4 to 53.6) | 13 (4.9 to 24.3) |
| South Sudan | 2.9 (1.7 to 4.7) | 3.2 (1.9 to 5) | 74.3 (42.9 to 120.3) | 81 (47.4 to 122.9) |
| Sudan | 3.2 (1.8 to 5.4) | 3 (1.6 to 4.5) | 79.9 (45.6 to 137.8) | 71.6 (39.2 to 110.8) |

Abbreviation: PMP, particulate matter pollution. ASMR, Age-Standardized mortality Rate. DALYs, Disability-adjusted life years. ASDR, Age-Standardized DALYs Rate.

Supplement table 2: The net drift of lung cancer burden attributable to PMP, APMP, and HAP in glocal.

| Risk | Sex | Measure | Net Drift (%/year) | 95% Confidence Interval |
|---|---|---|---|---|
| APMP | Both | Deaths | 0.9 | 0.8to1 |
| APMP | Male | Deaths | 0.4 | 0.3to0.6 |
| APMP | Female | Deaths | 1.6 | 1.5to1.7 |
| HAP | Both | Deaths | -4 | -4.1to-3.9 |
| HAP | Male | Deaths | -4.3 | -4.5to-4.1 |
| HAP | Female | Deaths | -3.7 | -3.8to-3.6 |
| PMP | Both | Deaths | -0.8 | -0.9to-0.7 |
| PMP | Male | Deaths | -1.1 | -1.2to-1 |
| PMP | Female | Deaths | -0.5 | -0.5to-0.4 |
| APMP | Both | DALYs | 0.9 | 0.7to1 |
| APMP | Male | DALYs | 0.4 | 0.3to0.6 |
| APMP | Female | DALYs | 1.6 | 1.5to1.7 |
| HAP | Both | DALYs | -4 | -4.2to-3.8 |
| HAP | Male | DALYs | -4.2 | -4.5to-4 |
| HAP | Female | DALYs | -3.7 | -3.9to-3.6 |
| PMP | Both | DALYs | -0.8 | -0.9to-0.7 |
| PMP | Male | DALYs | -1.1 | -1.2to-0.9 |
| PMP | Female | DALYs | -0.5 | -0.6to-0.4 |

Abbreviation: PMP, particulate matter pollution; APMP, ambient particulate matter pollution; HAP, household air pollution from solid fuels; DALY, Disability-adjusted life years.

Supplement table 3: The longitudinal age distribution in global lung cancer mortality rates and DALY rates due to PMP, APMP and HAP.

| Age | Risk | Sex | Measure | Rate ratio | 95% Confidence Interval |
|---|---|---|---|---|---|
| 50 to 54 | APMP | Both | Deaths | 4.468119 | 4.343110587 to 4.596725501 |
| 55 to 59 | APMP | Both | Deaths | 7.732109 | 7.55298619 to 7.915480396 |
| 60 to 64 | APMP | Both | Deaths | 12.3057 | 12.07025425 to 12.5457459 |
| 65 to 69 | APMP | Both | Deaths | 18.05232 | 17.73479429 to 18.37552475 |
| 70 to 74 | APMP | Both | Deaths | 25.44917 | 25.01757146 to 25.88821292 |
| 75 to 79 | APMP | Both | Deaths | 32.0077 | 31.45155421 to 32.57367373 |
| 80 to 84 | APMP | Both | Deaths | 36.55991 | 35.8503719 to 37.28348754 |
| 85 to 89 | APMP | Both | Deaths | 41.07622 | 40.05301813 to 42.12555703 |
| 90 to 94 | APMP | Both | Deaths | 41.4765 | 39.72678461 to 43.30327887 |
| 95 plus | APMP | Both | Deaths | 35.62231 | 32.63866254 to 38.87871245 |
| 50 to 54 | APMP | Male | Deaths | 7.279792 | 7.081267292 to 7.483882288 |
| 55 to 59 | APMP | Male | Deaths | 12.74464 | 12.45688267 to 13.03903981 |
| 60 to 64 | APMP | Male | Deaths | 20.27645 | 19.89905477 to 20.66099692 |
| 65 to 69 | APMP | Male | Deaths | 29.63591 | 29.12454975 to 30.15624735 |
| 70 to 74 | APMP | Male | Deaths | 41.47589 | 40.77877763 to 42.18492945 |
| 75 to 79 | APMP | Male | Deaths | 52.055 | 51.14609886 to 52.98005632 |
| 80 to 84 | APMP | Male | Deaths | 59.76642 | 58.57296205 to 60.98419214 |
| 85 to 89 | APMP | Male | Deaths | 69.9124 | 68.06591778 to 71.80897831 |
| 90 to 94 | APMP | Male | Deaths | 70.53382 | 67.19528085 to 74.03822622 |
| 95 plus | APMP | Male | Deaths | 49.40523 | 44.0235782 to 55.44476917 |
| 50 to 54 | APMP | Female | Deaths | 1.668079 | 1.615170954 to 1.722720584 |
| 55 to 59 | APMP | Female | Deaths | 2.87773 | 2.80194221 to 2.95556757 |
| 60 to 64 | APMP | Female | Deaths | 4.7765 | 4.672122911 to 4.883208323 |
| 65 to 69 | APMP | Female | Deaths | 7.47285 | 7.326855962 to 7.621752385 |
| 70 to 74 | APMP | Female | Deaths | 11.55438 | 11.34373407 to 11.7689476 |
| 75 to 79 | APMP | Female | Deaths | 16.02287 | 15.73462697 to 16.31639017 |
| 80 to 84 | APMP | Female | Deaths | 20.29295 | 19.90550382 to 20.68793466 |

| | | | | | |
|---|---|---|---|---|---|
| 85 to 89 | APMP | Female | Deaths | 24.12097 | 23.5632481 to 24.6919008 |
| 90 to 94 | APMP | Female | Deaths | 27.50534 | 26.51042855 to 28.53758049 |
| 95 plus | APMP | Female | Deaths | 30.06566 | 28.20570848 to 32.04825196 |
| 50 to 54 | HAP | Both | Deaths | 9.683239 | 9.410181954 to 9.964219501 |
| 55 to 59 | HAP | Both | Deaths | 12.42474 | 12.12174199 to 12.73531896 |
| 60 to 64 | HAP | Both | Deaths | 14.74662 | 14.45077371 to 15.0485202 |
| 65 to 69 | HAP | Both | Deaths | 16.47525 | 16.16747486 to 16.7888831 |
| 70 to 74 | HAP | Both | Deaths | 17.12716 | 16.80899928 to 17.45134257 |
| 75 to 79 | HAP | Both | Deaths | 15.08966 | 14.77547971 to 15.41052642 |
| 80 to 84 | HAP | Both | Deaths | 11.84738 | 11.53574823 to 12.16742189 |
| 85 to 89 | HAP | Both | Deaths | 9.837893 | 9.474976605 to 10.2147107 |
| 90 to 94 | HAP | Both | Deaths | 6.644022 | 6.210826898 to 7.1074317 |
| 95 plus | HAP | Both | Deaths | 3.109511 | 2.637517491 to 3.665969659 |
| 50 to 54 | HAP | Male | Deaths | 13.73418 | 13.31046679 to 14.17139059 |
| 55 to 59 | HAP | Male | Deaths | 18.01731 | 17.53705572 to 18.51072527 |
| 60 to 64 | HAP | Male | Deaths | 21.62781 | 21.15503885 to 22.11114783 |
| 65 to 69 | HAP | Male | Deaths | 24.42406 | 23.92466366 to 24.93388745 |
| 70 to 74 | HAP | Male | Deaths | 25.30576 | 24.78656176 to 25.83582712 |
| 75 to 79 | HAP | Male | Deaths | 22.26299 | 21.74278495 to 22.79564577 |
| 80 to 84 | HAP | Male | Deaths | 17.58791 | 17.05481206 to 18.13767187 |
| 85 to 89 | HAP | Male | Deaths | 15.8178 | 15.1349349 to 16.53148393 |
| 90 to 94 | HAP | Male | Deaths | 11.10049 | 10.21460599 to 12.06319952 |
| 95 plus | HAP | Male | Deaths | 3.835019 | 2.994227394 to 4.911907494 |
| 50 to 54 | HAP | Female | Deaths | 5.589836 | 5.429454451 to 5.754954116 |
| 55 to 59 | HAP | Female | Deaths | 6.945249 | 6.772379075 to 7.122530989 |
| 60 to 64 | HAP | Female | Deaths | 8.209231 | 8.039847589 to 8.382183665 |
| 65 to 69 | HAP | Female | Deaths | 9.209286 | 9.034206477 to 9.387759423 |
| 70 to 74 | HAP | Female | Deaths | 10.04911 | 9.862887005 to 10.23885088 |
| 75 to 79 | HAP | Female | Deaths | 9.390368 | 9.201329448 to 9.583290317 |

| | | | | | |
|---|---|---|---|---|---|
| 80 to 84 | HAP | Female | Deaths | 7.846715 | 7.655825679 to 8.042363526 |
| 85 to 89 | HAP | Female | Deaths | 6.350503 | 6.139557351 to 6.568695668 |
| 90 to 94 | HAP | Female | Deaths | 4.513769 | 4.264500922 to 4.777608161 |
| 95 plus | HAP | Female | Deaths | 2.81812 | 2.504603636 to 3.170879982 |
| 50 to 54 | PMP | Both | Deaths | 11.89015 | 11.65831024 to 12.12660718 |
| 55 to 59 | PMP | Both | Deaths | 18.24438 | 17.9440178 to 18.54976059 |
| 60 to 64 | PMP | Both | Deaths | 25.94024 | 25.58714506 to 26.29821124 |
| 65 to 69 | PMP | Both | Deaths | 34.42592 | 33.99127067 to 34.86612656 |
| 70 to 74 | PMP | Both | Deaths | 43.5182 | 42.9817269 to 44.06136393 |
| 75 to 79 | PMP | Both | Deaths | 48.44195 | 47.81140888 to 49.08079879 |
| 80 to 84 | PMP | Both | Deaths | 49.04051 | 48.29847381 to 49.7939419 |
| 85 to 89 | PMP | Both | Deaths | 50.28294 | 49.27747343 to 51.30892 |
| 90 to 94 | PMP | Both | Deaths | 45.1871 | 43.63671755 to 46.79257379 |
| 95 plus | PMP | Both | Deaths | 33.39487 | 31.01148504 to 35.96142423 |
| 50 to 54 | PMP | Male | Deaths | 17.85398 | 17.49740544 to 18.21781826 |
| 55 to 59 | PMP | Male | Deaths | 28.00975 | 27.538116 to 28.48945268 |
| 60 to 64 | PMP | Male | Deaths | 40.25828 | 39.69928538 to 40.82513725 |
| 65 to 69 | PMP | Male | Deaths | 53.85591 | 53.15779345 to 54.56320291 |
| 70 to 74 | PMP | Male | Deaths | 68.13057 | 67.26057613 to 69.01182226 |
| 75 to 79 | PMP | Male | Deaths | 76.2747 | 75.23315211 to 77.33067563 |
| 80 to 84 | PMP | Male | Deaths | 78.23092 | 76.9607587 to 79.52204327 |
| 85 to 89 | PMP | Male | Deaths | 84.86159 | 82.99412817 to 86.7710711 |
| 90 to 94 | PMP | Male | Deaths | 77.32868 | 74.24417202 to 80.54134377 |
| 95 plus | PMP | Male | Deaths | 45.99459 | 41.55004202 to 50.91455795 |
| 50 to 54 | PMP | Female | Deaths | 5.84739 | 5.729396347 to 5.967814522 |
| 55 to 59 | PMP | Female | Deaths | 8.664113 | 8.515753684 to 8.815056574 |
| 60 to 64 | PMP | Female | Deaths | 12.33398 | 12.1580755 to 12.51243006 |
| 65 to 69 | PMP | Female | Deaths | 16.67259 | 16.4559618 to 16.89207904 |
| 70 to 74 | PMP | Female | Deaths | 22.24514 | 21.96963027 to 22.52410964 |

| | | | | | |
|---|---|---|---|---|---|
| 75 to 79 | PMP | Female | Deaths | 26.33622 | 26.00202461 to 26.67471858 |
| 80 to 84 | PMP | Female | Deaths | 28.61049 | 28.20680219 to 29.01996437 |
| 85 to 89 | PMP | Female | Deaths | 29.89966 | 29.36736496 to 30.44160366 |
| 90 to 94 | PMP | Female | Deaths | 29.50671 | 28.67190372 to 30.36582464 |
| 95 plus | PMP | Female | Deaths | 27.83283 | 26.41767927 to 29.32378971 |
| 50 to 54 | PMP | Both | DALYs | 172.6715 | 168.9355329 to 176.4901017 |
| 55 to 59 | PMP | Both | DALYs | 262.2046 | 257.2195056 to 267.2862326 |
| 60 to 64 | PMP | Both | DALYs | 359.5051 | 353.6411612 to 365.4662174 |
| 65 to 69 | PMP | Both | DALYs | 444.0505 | 437.0261402 to 451.1877884 |
| 70 to 74 | PMP | Both | DALYs | 515.5498 | 507.2540856 to 523.9811462 |
| 75 to 79 | PMP | Both | DALYs | 518.9769 | 509.8115859 to 528.3068993 |
| 80 to 84 | PMP | Both | DALYs | 464.6801 | 454.5293366 to 475.0576015 |
| 85 to 89 | PMP | Both | DALYs | 414.6801 | 401.5339098 to 428.256617 |
| 90 to 94 | PMP | Both | DALYs | 365.084 | 344.0806823 to 387.3692967 |
| 95 plus | PMP | Both | DALYs | 292.2928 | 257.1356353 to 332.2568307 |
| 50 to 54 | PMP | Male | DALYs | 281.392 | 275.27129 to 287.648904 |
| 55 to 59 | PMP | Male | DALYs | 432.1948 | 423.9271948 to 440.6235614 |
| 60 to 64 | PMP | Male | DALYs | 592.3402 | 582.6473282 to 602.1944175 |
| 65 to 69 | PMP | Male | DALYs | 729.2353 | 717.5804817 to 741.0793784 |
| 70 to 74 | PMP | Male | DALYs | 840.7413 | 826.9480809 to 854.7646466 |
| 75 to 79 | PMP | Male | DALYs | 845.1479 | 829.70354 to 860.8797485 |
| 80 to 84 | PMP | Male | DALYs | 760.8261 | 743.1413781 to 778.9317444 |
| 85 to 89 | PMP | Male | DALYs | 706.7302 | 682.1081673 to 732.2409638 |
| 90 to 94 | PMP | Male | DALYs | 621.8941 | 580.2090688 to 666.5739882 |
| 95 plus | PMP | Male | DALYs | 405.4226 | 340.1379134 to 483.237811 |
| 50 to 54 | PMP | Female | DALYs | 64.18449 | 62.66503963 to 65.74077841 |
| 55 to 59 | PMP | Female | DALYs | 97.2442 | 95.22469579 to 99.30653556 |
| 60 to 64 | PMP | Female | DALYs | 139.1496 | 136.6517679 to 141.6931434 |
| 65 to 69 | PMP | Female | DALYs | 183.1341 | 180.0231773 to 186.2987091 |

| | | | | | |
|---|---|---|---|---|---|
| 70 to 74 | PMP | Female | DALYs | 233.0444 | 229.1323758 to 237.0232668 |
| 75 to 79 | PMP | Female | DALYs | 258.3653 | 253.7885947 to 263.0246053 |
| 80 to 84 | PMP | Female | DALYs | 256.5434 | 251.2592027 to 261.9387096 |
| 85 to 89 | PMP | Female | DALYs | 242.3214 | 235.535529 to 249.3027462 |
| 90 to 94 | PMP | Female | DALYs | 240.5686 | 229.3416705 to 252.3450692 |
| 95 plus | PMP | Female | DALYs | 245.4452 | 224.6210524 to 268.1999743 |
| 50 to 54 | HAP | Both | DALYs | 372.546 | 364.2000024 to 381.0832509 |
| 55 to 59 | HAP | Both | DALYs | 419.1341 | 410.5740542 to 427.8725276 |
| 60 to 64 | HAP | Both | DALYs | 428.7566 | 421.3593898 to 436.283712 |
| 65 to 69 | HAP | Both | DALYs | 403.4377 | 396.6504982 to 410.3410338 |
| 70 to 74 | HAP | Both | DALYs | 345.9297 | 339.7995165 to 352.1704482 |
| 75 to 79 | HAP | Both | DALYs | 244.7687 | 239.5006958 to 250.152513 |
| 80 to 84 | HAP | Both | DALYs | 150.9698 | 146.4095012 to 155.6721423 |
| 85 to 89 | HAP | Both | DALYs | 99.75603 | 95.02818459 to 104.7191012 |
| 90 to 94 | HAP | Both | DALYs | 58.76873 | 53.4173448 to 64.65622843 |
| 95 plus | HAP | Both | DALYs | 25.9601 | 20.29764767 to 33.20222348 |
| 50 to 54 | HAP | Male | DALYs | 528.3272 | 515.317118 to 541.6657392 |
| 55 to 59 | HAP | Male | DALYs | 607.6108 | 593.9845953 to 621.5496571 |
| 60 to 64 | HAP | Male | DALYs | 628.7147 | 616.8488488 to 640.8088113 |
| 65 to 69 | HAP | Male | DALYs | 598.2844 | 587.2351286 to 609.5414715 |
| 70 to 74 | HAP | Male | DALYs | 511.5409 | 501.5020234 to 521.7807339 |
| 75 to 79 | HAP | Male | DALYs | 361.8255 | 353.0524977 to 370.8164473 |
| 80 to 84 | HAP | Male | DALYs | 224.531 | 216.6608994 to 232.6869414 |
| 85 to 89 | HAP | Male | DALYs | 160.514 | 151.5479998 to 170.0104151 |
| 90 to 94 | HAP | Male | DALYs | 98.47536 | 87.44604341 to 110.8957827 |
| 95 plus | HAP | Male | DALYs | 32.02236 | 22.04942852 to 46.50604602 |
| 50 to 54 | HAP | Female | DALYs | 215.0222 | 210.0479122 to 220.1142759 |
| 55 to 59 | HAP | Female | DALYs | 234.197 | 229.2442341 to 239.2568384 |
| 60 to 64 | HAP | Female | DALYs | 238.5341 | 234.2340188 to 242.9130396 |

| | | | | | |
|---|---|---|---|---|---|
| 65 to 69 | HAP | Female | DALYs | 225.207 | 221.2821129 to 229.2014952 |
| 70 to 74 | HAP | Female | DALYs | 202.619 | 198.9707366 to 206.3341907 |
| 75 to 79 | HAP | Female | DALYs | 151.8433 | 148.6333198 to 155.1226773 |
| 80 to 84 | HAP | Female | DALYs | 99.74496 | 96.93216489 to 102.6393684 |
| 85 to 89 | HAP | Female | DALYs | 64.35227 | 61.58368104 to 67.24532475 |
| 90 to 94 | HAP | Female | DALYs | 39.79225 | 36.69653102 to 43.14911687 |
| 95 plus | HAP | Female | DALYs | 23.51059 | 19.68339175 to 28.08193723 |
| 50 to 54 | PMP | Both | DALYs | 457.6025 | 450.3953909 to 464.9248729 |
| 55 to 59 | PMP | Both | DALYs | 616.496 | 607.8069624 to 625.3092876 |
| 60 to 64 | PMP | Both | DALYs | 755.6539 | 746.5423867 to 764.8765378 |
| 65 to 69 | PMP | Both | DALYs | 844.3255 | 834.3876869 to 854.3816195 |
| 70 to 74 | PMP | Both | DALYs | 878.9435 | 868.279107 to 889.7388219 |
| 75 to 79 | PMP | Both | DALYs | 783.6826 | 772.8819426 to 794.6341638 |
| 80 to 84 | PMP | Both | DALYs | 621.961 | 610.8873461 to 633.2354 |
| 85 to 89 | PMP | Both | DALYs | 506.4315 | 492.9719546 to 520.2585926 |
| 90 to 94 | PMP | Both | DALYs | 396.2217 | 376.7475607 to 416.7024811 |
| 95 plus | PMP | Both | DALYs | 273.1706 | 243.8668491 to 305.9956364 |
| 50 to 54 | PMP | Male | DALYs | 687.5936 | 676.2618387 to 699.1152927 |
| 55 to 59 | PMP | Male | DALYs | 946.9342 | 932.9810323 to 961.0960009 |
| 60 to 64 | PMP | Male | DALYs | 1173.184 | 1158.443136 to 1188.112107 |
| 65 to 69 | PMP | Male | DALYs | 1321.769 | 1305.470933 to 1338.271077 |
| 70 to 74 | PMP | Male | DALYs | 1377.328 | 1359.67826 to 1395.206203 |
| 75 to 79 | PMP | Male | DALYs | 1236.067 | 1217.827784 to 1254.578989 |
| 80 to 84 | PMP | Male | DALYs | 993.9144 | 974.4606568 to 1013.756557 |
| 85 to 89 | PMP | Male | DALYs | 855.6947 | 830.0168412 to 882.1669133 |
| 90 to 94 | PMP | Male | DALYs | 679.4233 | 639.5668132 to 721.763533 |
| 95 plus | PMP | Male | DALYs | 376.2314 | 320.5790831 to 441.544886 |
| 50 to 54 | PMP | Female | DALYs | 224.3708 | 220.8122797 to 227.9866471 |
| 55 to 59 | PMP | Female | DALYs | 291.9774 | 287.8215683 to 296.1932923 |

| | | | | | |
|---|---|---|---|---|---|
| 60 to 64 | PMP | Female | DALYs | 358.4527 | 354.0485274 to 362.911726 |
| 65 to 69 | PMP | Female | DALYs | 407.6752 | 402.8602396 to 412.5477178 |
| 70 to 74 | PMP | Female | DALYs | 447.7303 | 442.3963754 to 453.1285316 |
| 75 to 79 | PMP | Female | DALYs | 423.9898 | 418.4338662 to 429.6194902 |
| 80 to 84 | PMP | Female | DALYs | 361.2285 | 355.4307346 to 367.1207899 |
| 85 to 89 | PMP | Female | DALYs | 300.1703 | 293.3316268 to 307.1683371 |
| 90 to 94 | PMP | Female | DALYs | 257.542 | 247.5220208 to 267.9675977 |
| 95 plus | PMP | Female | DALYs | 226.989 | 210.2003716 to 245.1184328 |

Abbreviation: PMP, particulate matter pollution; APMP, ambient particulate matter pollution; HAP, household air pollution from solid fuels; DALY, Disability-adjusted life years.

Supplement table 4: The period distribution in global lung cancer mortality rates and DALY rates due to PMP, APMP and HAP.

| Period | Risk | Sex | Measure | Rate Ratio | 95% Confidence Interval |
|---|---|---|---|---|---|
| 1990 to 1994 | APMP | Both | Deaths | 0.953283 | 0.932091389 to 0.974957197 |
| 1995 to 1999 | APMP | Both | Deaths | 0.926162 | 0.907453426 to 0.945257266 |
| 2000 to 2004 | APMP | Both | Deaths | 0.956729 | 0.939584888 to 0.97418557 |
| 2005 to 2009 | APMP | Both | Deaths | 1 | 1 to 1 |
| 2010 to 2014 | APMP | Both | Deaths | 1.110187 | 1.091836897 to 1.128845697 |
| 2015 to 2019 | APMP | Both | Deaths | 1.146606 | 1.125418327 to 1.168193202 |
| 1990 to 1994 | APMP | Male | Deaths | 1.02033 | 0.995897515 to 1.045362544 |
| 1995 to 1999 | APMP | Male | Deaths | 0.965663 | 0.945301465 to 0.986462508 |
| 2000 to 2004 | APMP | Male | Deaths | 0.976392 | 0.958664462 to 0.994446396 |
| 2005 to 2009 | APMP | Male | Deaths | 1 | 1 to 1 |
| 2010 to 2014 | APMP | Male | Deaths | 1.09564 | 1.077149039 to 1.114447404 |
| 2015 to 2019 | APMP | Male | Deaths | 1.097816 | 1.076209432 to 1.119857257 |
| 1990 to 1994 | APMP | Female | Deaths | 0.819298 | 0.801256121 to 0.837745914 |
| 1995 to 1999 | APMP | Female | Deaths | 0.855466 | 0.837931101 to 0.873367007 |
| 2000 to 2004 | APMP | Female | Deaths | 0.926299 | 0.909062397 to 0.943863278 |
| 2005 to 2009 | APMP | Female | Deaths | 1 | 1 to 1 |
| 2010 to 2014 | APMP | Female | Deaths | 1.114303 | 1.095509819 to 1.133419493 |
| 2015 to 2019 | APMP | Female | Deaths | 1.196562 | 1.175208367 to 1.218304604 |
| 1990 to 1994 | HAP | Both | Deaths | 1.35594 | 1.320728779 to 1.392089933 |
| 1995 to 1999 | HAP | Both | Deaths | 1.29508 | 1.26597696 to 1.324852465 |
| 2000 to 2004 | HAP | Both | Deaths | 1.220992 | 1.197299922 to 1.245153415 |
| 2005 to 2009 | HAP | Both | Deaths | 1 | 1 to 1 |
| 2010 to 2014 | HAP | Both | Deaths | 0.720797 | 0.705715502 to 0.736201287 |
| 2015 to 2019 | HAP | Both | Deaths | 0.480582 | 0.468083517 to 0.493414836 |
| 1990 to 1994 | HAP | Male | Deaths | 1.398845 | 1.352768887 to 1.446489832 |
| 1995 to 1999 | HAP | Male | Deaths | 1.314543 | 1.278817181 to 1.351266498 |
| 2000 to 2004 | HAP | Male | Deaths | 1.23238 | 1.205041865 to 1.260337799 |

| | | | | | |
|---|---|---|---|---|---|
| 2005 to 2009 | HAP | Male | Deaths | 1 | 1 to 1 |
| 2010 to 2014 | HAP | Male | Deaths | 0.713193 | 0.696188216 to 0.730613028 |
| 2015 to 2019 | HAP | Male | Deaths | 0.45811 | 0.443891385 to 0.472785 |
| 1990 to 1994 | HAP | Female | Deaths | 1.309074 | 1.279225088 to 1.339619206 |
| 1995 to 1999 | HAP | Female | Deaths | 1.286918 | 1.260300896 to 1.314096405 |
| 2000 to 2004 | HAP | Female | Deaths | 1.215841 | 1.193023696 to 1.239094454 |
| 2005 to 2009 | HAP | Female | Deaths | 1 | 1 to 1 |
| 2010 to 2014 | HAP | Female | Deaths | 0.722492 | 0.707864815 to 0.737420646 |
| 2015 to 2019 | HAP | Female | Deaths | 0.507606 | 0.495461771 to 0.520047637 |
| 1990 to 1994 | PMP | Both | Deaths | 1.10814 | 1.089986716 to 1.126596374 |
| 1995 to 1999 | PMP | Both | Deaths | 1.065547 | 1.049938825 to 1.08138649 |
| 2000 to 2004 | PMP | Both | Deaths | 1.055554 | 1.041890193 to 1.069397294 |
| 2005 to 2009 | PMP | Both | Deaths | 1 | 1 to 1 |
| 2010 to 2014 | PMP | Both | Deaths | 0.963964 | 0.951774666 to 0.97630993 |
| 2015 to 2019 | PMP | Both | Deaths | 0.90732 | 0.894240348 to 0.920591178 |
| 1990 to 1994 | PMP | Male | Deaths | 1.15905 | 1.137327423 to 1.181188458 |
| 1995 to 1999 | PMP | Male | Deaths | 1.091284 | 1.073652985 to 1.109204899 |
| 2000 to 2004 | PMP | Male | Deaths | 1.067749 | 1.053069369 to 1.082632712 |
| 2005 to 2009 | PMP | Male | Deaths | 1 | 1 to 1 |
| 2010 to 2014 | PMP | Male | Deaths | 0.958129 | 0.945237723 to 0.97119613 |
| 2015 to 2019 | PMP | Male | Deaths | 0.877215 | 0.863157914 to 0.891500806 |
| 1990 to 1994 | PMP | Female | Deaths | 1.032541 | 1.017225196 to 1.048087462 |
| 1995 to 1999 | PMP | Female | Deaths | 1.038481 | 1.024208361 to 1.052953137 |
| 2000 to 2004 | PMP | Female | Deaths | 1.046772 | 1.033591414 to 1.060121456 |
| 2005 to 2009 | PMP | Female | Deaths | 1 | 1 to 1 |
| 2010 to 2014 | PMP | Female | Deaths | 0.9539 | 0.942208852 to 0.965735218 |
| 2015 to 2019 | PMP | Female | Deaths | 0.930899 | 0.91856833 to 0.943396077 |
| 1990 to 1994 | PMP | Both | DALYs | 0.955902 | 0.931699732 to 0.980733975 |
| 1995 to 1999 | PMP | Both | DALYs | 0.925379 | 0.90600039 to 0.945171723 |

| | | | | | |
|---|---|---|---|---|---|
| 2000 to 2004 | PMP | Both | DALYs | 0.952806 | 0.936770246 to 0.969115353 |
| 2005 to 2009 | PMP | Both | DALYs | 1 | 1 to 1 |
| 2010 to 2014 | PMP | Both | DALYs | 1.112063 | 1.094496957 to 1.12991134 |
| 2015 to 2019 | PMP | Both | DALYs | 1.1489 | 1.125765447 to 1.172510896 |
| 1990 to 1994 | PMP | Male | DALYs | 1.023528 | 0.993236168 to 1.054744677 |
| 1995 to 1999 | PMP | Male | DALYs | 0.96631 | 0.943609206 to 0.989556164 |
| 2000 to 2004 | PMP | Male | DALYs | 0.972994 | 0.955594061 to 0.990711765 |
| 2005 to 2009 | PMP | Male | DALYs | 1 | 1 to 1 |
| 2010 to 2014 | PMP | Male | DALYs | 1.097151 | 1.078559825 to 1.116062824 |
| 2015 to 2019 | PMP | Male | DALYs | 1.101457 | 1.076227767 to 1.127276743 |
| 1990 to 1994 | PMP | Female | DALYs | 0.819871 | 0.801650663 to 0.838505396 |
| 1995 to 1999 | PMP | Female | DALYs | 0.85251 | 0.835861931 to 0.869489146 |
| 2000 to 2004 | PMP | Female | DALYs | 0.922627 | 0.907203865 to 0.938313084 |
| 2005 to 2009 | PMP | Female | DALYs | 1 | 1 to 1 |
| 2010 to 2014 | PMP | Female | DALYs | 1.114715 | 1.097594409 to 1.13210281 |
| 2015 to 2019 | PMP | Female | DALYs | 1.19412 | 1.172616688 to 1.216016917 |
| 1990 to 1994 | HAP | Both | DALYs | 1.361096 | 1.316992583 to 1.406675855 |
| 1995 to 1999 | HAP | Both | DALYs | 1.295935 | 1.26322682 to 1.32949036 |
| 2000 to 2004 | HAP | Both | DALYs | 1.216339 | 1.193301803 to 1.239821684 |
| 2005 to 2009 | HAP | Both | DALYs | 1 | 1 to 1 |
| 2010 to 2014 | HAP | Both | DALYs | 0.721007 | 0.706363446 to 0.735953221 |
| 2015 to 2019 | HAP | Both | DALYs | 0.486478 | 0.472693382 to 0.500664363 |
| 1990 to 1994 | HAP | Male | DALYs | 1.404198 | 1.343216989 to 1.467946608 |
| 1995 to 1999 | HAP | Male | DALYs | 1.316592 | 1.273860448 to 1.360756941 |
| 2000 to 2004 | HAP | Male | DALYs | 1.227373 | 1.199695725 to 1.25568781 |
| 2005 to 2009 | HAP | Male | DALYs | 1 | 1 to 1 |
| 2010 to 2014 | HAP | Male | DALYs | 0.713539 | 0.696430753 to 0.731066504 |
| 2015 to 2019 | HAP | Male | DALYs | 0.464716 | 0.448179255 to 0.481863661 |
| 1990 to 1994 | HAP | Female | DALYs | 1.315814 | 1.280286498 to 1.352327666 |

| | | | | | |
|---|---|---|---|---|---|
| 1995 to 1999 | HAP | Female | DALYs | 1.287611 | 1.258937684 to 1.316937178 |
| 2000 to 2004 | HAP | Female | DALYs | 1.212753 | 1.190719263 to 1.235194575 |
| 2005 to 2009 | HAP | Female | DALYs | 1 | 1 to 1 |
| 2010 to 2014 | HAP | Female | DALYs | 0.722847 | 0.70868069 to 0.737296386 |
| 2015 to 2019 | HAP | Female | DALYs | 0.513844 | 0.50076319 to 0.527267318 |
| 1990 to 1994 | PMP | Both | DALYs | 1.107726 | 1.085563325 to 1.130341895 |
| 1995 to 1999 | PMP | Both | DALYs | 1.064526 | 1.047319006 to 1.082015311 |
| 2000 to 2004 | PMP | Both | DALYs | 1.052018 | 1.038623809 to 1.065585432 |
| 2005 to 2009 | PMP | Both | DALYs | 1 | 1 to 1 |
| 2010 to 2014 | PMP | Both | DALYs | 0.962665 | 0.950552402 to 0.97493227 |
| 2015 to 2019 | PMP | Both | DALYs | 0.906764 | 0.89192949 to 0.921846076 |
| 1990 to 1994 | PMP | Male | DALYs | 1.159171 | 1.130687098 to 1.188373045 |
| 1995 to 1999 | PMP | Male | DALYs | 1.091529 | 1.070725114 to 1.112736687 |
| 2000 to 2004 | PMP | Male | DALYs | 1.064303 | 1.049276564 to 1.079544842 |
| 2005 to 2009 | PMP | Male | DALYs | 1 | 1 to 1 |
| 2010 to 2014 | PMP | Male | DALYs | 0.956752 | 0.943378898 to 0.970315181 |
| 2015 to 2019 | PMP | Male | DALYs | 0.877825 | 0.860874967 to 0.895109129 |
| 1990 to 1994 | PMP | Female | DALYs | 1.031245 | 1.014400321 to 1.048370032 |
| 1995 to 1999 | PMP | Female | DALYs | 1.036423 | 1.021896959 to 1.051154995 |
| 2000 to 2004 | PMP | Female | DALYs | 1.04435 | 1.031941556 to 1.056908582 |
| 2005 to 2009 | PMP | Female | DALYs | 1 | 1 to 1 |
| 2010 to 2014 | PMP | Female | DALYs | 0.951981 | 0.940844585 to 0.963249302 |
| 2015 to 2019 | PMP | Female | DALYs | 0.928994 | 0.91595584 to 0.942218233 |

Abbreviation: PMP, particulate matter pollution; APMP, ambient particulate matter pollution; HAP, household air pollution from solid fuels; DALY, Disability-adjusted life years.

Supplement table 5: The cohort distribution in global lung cancer mortality rates and DALY rates due to PMP, APMP and HAP.

| Cohort | Risk | Sex | Measure | Rate Ratio | 95% Confidence Interval |
|---|---|---|---|---|---|
| 1890 to 1899 | APMP | Both | Deaths | 0.561252 | 0.398166228 to 0.791137438 |
| 1895 to 1904 | APMP | Both | Deaths | 0.585021 | 0.510765664 to 0.670070724 |
| 1900 to 1909 | APMP | Both | Deaths | 0.618031 | 0.577506256 to 0.66140003 |
| 1905 to 1914 | APMP | Both | Deaths | 0.690689 | 0.662506145 to 0.720070046 |
| 1910 to 1919 | APMP | Both | Deaths | 0.766551 | 0.743655476 to 0.790151036 |
| 1915 to 1924 | APMP | Both | Deaths | 0.834947 | 0.8150228 to 0.855357881 |
| 1920 to 1929 | APMP | Both | Deaths | 0.933081 | 0.91436907 to 0.952176721 |
| 1925 to 1934 | APMP | Both | Deaths | 1 | 1 to 1 |
| 1930 to 1939 | APMP | Both | Deaths | 1.00914 | 0.990800067 to 1.027820309 |
| 1935 to 1944 | APMP | Both | Deaths | 0.98623 | 0.967540178 to 1.005281652 |
| 1940 to 1949 | APMP | Both | Deaths | 0.991171 | 0.970995452 to 1.011765976 |
| 1945 to 1954 | APMP | Both | Deaths | 1.025029 | 1.002332107 to 1.048240318 |
| 1950 to 1959 | APMP | Both | Deaths | 1.018529 | 0.992110236 to 1.045650238 |
| 1955 to 1964 | APMP | Both | Deaths | 0.91368 | 0.882679644 to 0.945769382 |
| 1960 to 1969 | APMP | Both | Deaths | 0.918102 | 0.873963427 to 0.964468813 |
| 1890 to 1899 | APMP | Male | Deaths | 0.729829 | 0.474143046 to 1.12339573 |
| 1895 to 1904 | APMP | Male | Deaths | 0.672402 | 0.574914751 to 0.786421042 |
| 1900 to 1909 | APMP | Male | Deaths | 0.682868 | 0.634020315 to 0.735479068 |
| 1905 to 1914 | APMP | Male | Deaths | 0.758865 | 0.726106122 to 0.793100795 |
| 1910 to 1919 | APMP | Male | Deaths | 0.815725 | 0.790485752 to 0.841769867 |
| 1915 to 1924 | APMP | Male | Deaths | 0.867711 | 0.846605097 to 0.889343685 |
| 1920 to 1929 | APMP | Male | Deaths | 0.957076 | 0.93779081 to 0.976757328 |
| 1925 to 1934 | APMP | Male | Deaths | 1 | 1 to 1 |
| 1930 to 1939 | APMP | Male | Deaths | 0.992487 | 0.974571735 to 1.010731651 |
| 1935 to 1944 | APMP | Male | Deaths | 0.954307 | 0.936435615 to 0.972518762 |
| 1940 to 1949 | APMP | Male | Deaths | 0.930863 | 0.912196262 to 0.949912548 |
| 1945 to 1954 | APMP | Male | Deaths | 0.951491 | 0.930803175 to 0.972639581 |

| | | | | | |
|---|---|---|---|---|---|
| 1950 to 1959 | APMP | Male | Deaths | 0.930866 | 0.907168334 to 0.955182195 |
| 1955 to 1964 | APMP | Male | Deaths | 0.820586 | 0.793134835 to 0.848986866 |
| 1960 to 1969 | APMP | Male | Deaths | 0.808761 | 0.77003599 to 0.849433558 |
| 1890 to 1899 | APMP | Female | Deaths | 0.488348 | 0.37765994 to 0.631476264 |
| 1895 to 1904 | APMP | Female | Deaths | 0.543478 | 0.486646328 to 0.606945837 |
| 1900 to 1909 | APMP | Female | Deaths | 0.586915 | 0.553087814 to 0.622810954 |
| 1905 to 1914 | APMP | Female | Deaths | 0.653996 | 0.629261708 to 0.679702636 |
| 1910 to 1919 | APMP | Female | Deaths | 0.744898 | 0.723398131 to 0.767036224 |
| 1915 to 1924 | APMP | Female | Deaths | 0.82644 | 0.806712213 to 0.846649252 |
| 1920 to 1929 | APMP | Female | Deaths | 0.92012 | 0.901043273 to 0.93960087 |
| 1925 to 1934 | APMP | Female | Deaths | 1 | 1 to 1 |
| 1930 to 1939 | APMP | Female | Deaths | 1.050578 | 1.030219977 to 1.071338962 |
| 1935 to 1944 | APMP | Female | Deaths | 1.07954 | 1.057373405 to 1.102171751 |
| 1940 to 1949 | APMP | Female | Deaths | 1.180953 | 1.154622228 to 1.207884158 |
| 1945 to 1954 | APMP | Female | Deaths | 1.299925 | 1.268021275 to 1.332630833 |
| 1950 to 1959 | APMP | Female | Deaths | 1.380866 | 1.341059374 to 1.421853577 |
| 1955 to 1964 | APMP | Female | Deaths | 1.323315 | 1.274090297 to 1.374440966 |
| 1960 to 1969 | APMP | Female | Deaths | 1.421873 | 1.348987406 to 1.498695648 |
| 1890 to 1899 | HAP | Both | Deaths | 3.283171 | 2.007298599 to 5.370010668 |
| 1895 to 1904 | HAP | Both | Deaths | 2.675773 | 2.259569038 to 3.168640831 |
| 1900 to 1909 | HAP | Both | Deaths | 2.23164 | 2.060350053 to 2.417169313 |
| 1905 to 1914 | HAP | Both | Deaths | 1.842665 | 1.753698424 to 1.93614389 |
| 1910 to 1919 | HAP | Both | Deaths | 1.716515 | 1.658879941 to 1.776153221 |
| 1915 to 1924 | HAP | Both | Deaths | 1.464417 | 1.426163057 to 1.503697106 |
| 1920 to 1929 | HAP | Both | Deaths | 1.199651 | 1.173093064 to 1.226810111 |
| 1925 to 1934 | HAP | Both | Deaths | 1 | 1 to 1 |
| 1930 to 1939 | HAP | Both | Deaths | 0.814106 | 0.797507323 to 0.831050446 |
| 1935 to 1944 | HAP | Both | Deaths | 0.6192 | 0.605841266 to 0.632852666 |
| 1940 to 1949 | HAP | Both | Deaths | 0.48971 | 0.478116202 to 0.501585869 |

| | | | | | |
|---|---|---|---|---|---|
| 1945 to 1954 | HAP | Both | Deaths | 0.383297 | 0.373162188 to 0.393706435 |
| 1950 to 1959 | HAP | Both | Deaths | 0.301448 | 0.291951289 to 0.311254136 |
| 1955 to 1964 | HAP | Both | Deaths | 0.220446 | 0.211082676 to 0.230224805 |
| 1960 to 1969 | HAP | Both | Deaths | 0.183848 | 0.172214016 to 0.196267893 |
| 1890 to 1899 | HAP | Male | Deaths | 3.972222 | 1.919039377 to 8.222107189 |
| 1895 to 1904 | HAP | Male | Deaths | 3.015844 | 2.439191212 to 3.728823949 |
| 1900 to 1909 | HAP | Male | Deaths | 2.384523 | 2.164615597 to 2.626770542 |
| 1905 to 1914 | HAP | Male | Deaths | 1.964371 | 1.852511246 to 2.082986102 |
| 1910 to 1919 | HAP | Male | Deaths | 1.816159 | 1.746126344 to 1.888999645 |
| 1915 to 1924 | HAP | Male | Deaths | 1.527654 | 1.482709296 to 1.573961414 |
| 1920 to 1929 | HAP | Male | Deaths | 1.230906 | 1.200628484 to 1.261947555 |
| 1925 to 1934 | HAP | Male | Deaths | 1 | 1 to 1 |
| 1930 to 1939 | HAP | Male | Deaths | 0.80338 | 0.785336725 to 0.821837929 |
| 1935 to 1944 | HAP | Male | Deaths | 0.606534 | 0.592151875 to 0.621264909 |
| 1940 to 1949 | HAP | Male | Deaths | 0.473398 | 0.46109896 to 0.48602455 |
| 1945 to 1954 | HAP | Male | Deaths | 0.369042 | 0.358341716 to 0.380061607 |
| 1950 to 1959 | HAP | Male | Deaths | 0.290214 | 0.280187489 to 0.300598542 |
| 1955 to 1964 | HAP | Male | Deaths | 0.212083 | 0.202197182 to 0.222452385 |
| 1960 to 1969 | HAP | Male | Deaths | 0.172695 | 0.160511335 to 0.185804498 |
| 1890 to 1899 | HAP | Female | Deaths | 3.021595 | 2.123006614 to 4.300521303 |
| 1895 to 1904 | HAP | Female | Deaths | 2.501409 | 2.176622902 to 2.874659144 |
| 1900 to 1909 | HAP | Female | Deaths | 2.19749 | 2.051791818 to 2.353534602 |
| 1905 to 1914 | HAP | Female | Deaths | 1.811689 | 1.733959407 to 1.892902819 |
| 1910 to 1919 | HAP | Female | Deaths | 1.67326 | 1.621179524 to 1.727012851 |
| 1915 to 1924 | HAP | Female | Deaths | 1.429722 | 1.393891305 to 1.466474113 |
| 1920 to 1929 | HAP | Female | Deaths | 1.178565 | 1.152880156 to 1.204822714 |
| 1925 to 1934 | HAP | Female | Deaths | 1 | 1 to 1 |
| 1930 to 1939 | HAP | Female | Deaths | 0.833654 | 0.816613109 to 0.85105032 |
| 1935 to 1944 | HAP | Female | Deaths | 0.643953 | 0.629951206 to 0.658265253 |

| | | | | | |
|---|---|---|---|---|---|
| 1940 to 1949 | HAP | Female | Deaths | 0.52104 | 0.508559983 to 0.53382687 |
| 1945 to 1954 | HAP | Female | Deaths | 0.416033 | 0.404893436 to 0.427479159 |
| 1950 to 1959 | HAP | Female | Deaths | 0.330012 | 0.319488931 to 0.340880687 |
| 1955 to 1964 | HAP | Female | Deaths | 0.24379 | 0.233349877 to 0.254696952 |
| 1960 to 1969 | HAP | Female | Deaths | 0.214903 | 0.201647999 to 0.229029485 |
| 1890 to 1899 | PMP | Both | Deaths | 0.897163 | 0.682739853 to 1.178927263 |
| 1895 to 1904 | PMP | Both | Deaths | 0.899559 | 0.811404138 to 0.997290873 |
| 1900 to 1909 | PMP | Both | Deaths | 0.902355 | 0.858070831 to 0.948924342 |
| 1905 to 1914 | PMP | Both | Deaths | 0.923222 | 0.895055601 to 0.952275605 |
| 1910 to 1919 | PMP | Both | Deaths | 0.986749 | 0.9652133 to 1.008765774 |
| 1915 to 1924 | PMP | Both | Deaths | 1.003223 | 0.985874215 to 1.020876324 |
| 1920 to 1929 | PMP | Both | Deaths | 1.012169 | 0.997339015 to 1.027219606 |
| 1925 to 1934 | PMP | Both | Deaths | 1 | 1 to 1 |
| 1930 to 1939 | PMP | Both | Deaths | 0.939767 | 0.927212704 to 0.952490423 |
| 1935 to 1944 | PMP | Both | Deaths | 0.838339 | 0.826646359 to 0.850197754 |
| 1940 to 1949 | PMP | Both | Deaths | 0.770248 | 0.758667959 to 0.782005488 |
| 1945 to 1954 | PMP | Both | Deaths | 0.717021 | 0.705230239 to 0.729008908 |
| 1950 to 1959 | PMP | Both | Deaths | 0.649074 | 0.636517664 to 0.661878202 |
| 1955 to 1964 | PMP | Both | Deaths | 0.530866 | 0.517264365 to 0.544825206 |
| 1960 to 1969 | PMP | Both | Deaths | 0.485683 | 0.467744436 to 0.504310325 |
| 1890 to 1899 | PMP | Male | Deaths | 1.113477 | 0.775275638 to 1.599214606 |
| 1895 to 1904 | PMP | Male | Deaths | 1.011761 | 0.894576415 to 1.144295825 |
| 1900 to 1909 | PMP | Male | Deaths | 0.970144 | 0.916045688 to 1.027437676 |
| 1905 to 1914 | PMP | Male | Deaths | 0.988521 | 0.955163861 to 1.023043175 |
| 1910 to 1919 | PMP | Male | Deaths | 1.033919 | 1.009530624 to 1.058897199 |
| 1915 to 1924 | PMP | Male | Deaths | 1.033993 | 1.01500528 to 1.053335146 |
| 1920 to 1929 | PMP | Male | Deaths | 1.0334 | 1.017551113 to 1.049495502 |
| 1925 to 1934 | PMP | Male | Deaths | 1 | 1 to 1 |
| 1930 to 1939 | PMP | Male | Deaths | 0.929835 | 0.916971871 to 0.942877614 |

| | | | | | |
|---|---|---|---|---|---|
| 1935 to 1944 | PMP | Male | Deaths | 0.823227 | 0.81138619 to 0.835241138 |
| 1940 to 1949 | PMP | Male | Deaths | 0.742393 | 0.730912181 to 0.754054133 |
| 1945 to 1954 | PMP | Male | Deaths | 0.689243 | 0.677611018 to 0.701074084 |
| 1950 to 1959 | PMP | Male | Deaths | 0.621262 | 0.608930041 to 0.633842988 |
| 1955 to 1964 | PMP | Male | Deaths | 0.504862 | 0.4915464 to 0.518538626 |
| 1960 to 1969 | PMP | Male | Deaths | 0.455325 | 0.437826192 to 0.473522555 |
| 1890 to 1899 | PMP | Female | Deaths | 0.825756 | 0.67934028 to 1.003728628 |
| 1895 to 1904 | PMP | Female | Deaths | 0.859315 | 0.792047048 to 0.932296089 |
| 1900 to 1909 | PMP | Female | Deaths | 0.893157 | 0.856216635 to 0.931690644 |
| 1905 to 1914 | PMP | Female | Deaths | 0.911961 | 0.887471369 to 0.93712544 |
| 1910 to 1919 | PMP | Female | Deaths | 0.985702 | 0.965968105 to 1.005838926 |
| 1915 to 1924 | PMP | Female | Deaths | 1.007738 | 0.991189403 to 1.024562213 |
| 1920 to 1929 | PMP | Female | Deaths | 1.007358 | 0.992909042 to 1.022016521 |
| 1925 to 1934 | PMP | Female | Deaths | 1 | 1 to 1 |
| 1930 to 1939 | PMP | Female | Deaths | 0.959529 | 0.946652207 to 0.972581357 |
| 1935 to 1944 | PMP | Female | Deaths | 0.872727 | 0.860360781 to 0.885271783 |
| 1940 to 1949 | PMP | Female | Deaths | 0.836074 | 0.823211256 to 0.849138223 |
| 1945 to 1954 | PMP | Female | Deaths | 0.797508 | 0.784004016 to 0.811243624 |
| 1950 to 1959 | PMP | Female | Deaths | 0.739307 | 0.724572472 to 0.754340233 |
| 1955 to 1964 | PMP | Female | Deaths | 0.621086 | 0.60484309 to 0.63776482 |
| 1960 to 1969 | PMP | Female | Deaths | 0.593857 | 0.57205177 to 0.616494272 |
| 1890 to 1899 | APMP | Both | DALYs | 0.562213 | 0.338888186 to 0.932707774 |
| 1895 to 1904 | APMP | Both | DALYs | 0.581232 | 0.478441983 to 0.706106017 |
| 1900 to 1909 | APMP | Both | DALYs | 0.613806 | 0.559973929 to 0.672813822 |
| 1905 to 1914 | APMP | Both | DALYs | 0.686719 | 0.652124163 to 0.723148262 |
| 1910 to 1919 | APMP | Both | DALYs | 0.759148 | 0.733400952 to 0.785798832 |
| 1915 to 1924 | APMP | Both | DALYs | 0.831264 | 0.810319194 to 0.852750375 |
| 1920 to 1929 | APMP | Both | DALYs | 0.935629 | 0.916826892 to 0.954816538 |
| 1925 to 1934 | APMP | Both | DALYs | 1 | 1 to 1 |

| | | | | | |
|---|---|---|---|---|---|
| 1930 to 1939 | APMP | Both | DALYs | 1.003415 | 0.986160364 to 1.020971884 |
| 1935 to 1944 | APMP | Both | DALYs | 0.982533 | 0.965514294 to 0.999852489 |
| 1940 to 1949 | APMP | Both | DALYs | 0.987768 | 0.970055757 to 1.005804649 |
| 1945 to 1954 | APMP | Both | DALYs | 1.024423 | 1.005249564 to 1.04396163 |
| 1950 to 1959 | APMP | Both | DALYs | 1.014142 | 0.992919737 to 1.035818505 |
| 1955 to 1964 | APMP | Both | DALYs | 0.909499 | 0.885844246 to 0.933785916 |
| 1960 to 1969 | APMP | Both | DALYs | 0.914124 | 0.882128453 to 0.947279279 |
| 1890 to 1899 | APMP | Male | DALYs | 0.734998 | 0.381313126 to 1.41674157 |
| 1895 to 1904 | APMP | Male | DALYs | 0.666599 | 0.528523681 to 0.84074699 |
| 1900 to 1909 | APMP | Male | DALYs | 0.678307 | 0.611560391 to 0.752338265 |
| 1905 to 1914 | APMP | Male | DALYs | 0.755849 | 0.714466262 to 0.799628974 |
| 1910 to 1919 | APMP | Male | DALYs | 0.807776 | 0.778666513 to 0.837972961 |
| 1915 to 1924 | APMP | Male | DALYs | 0.862595 | 0.839915548 to 0.885887598 |
| 1920 to 1929 | APMP | Male | DALYs | 0.958025 | 0.938253718 to 0.978213892 |
| 1925 to 1934 | APMP | Male | DALYs | 1 | 1 to 1 |
| 1930 to 1939 | APMP | Male | DALYs | 0.987361 | 0.970102088 to 1.004926697 |
| 1935 to 1944 | APMP | Male | DALYs | 0.951426 | 0.934710569 to 0.968439827 |
| 1940 to 1949 | APMP | Male | DALYs | 0.927449 | 0.910584999 to 0.944625147 |
| 1945 to 1954 | APMP | Male | DALYs | 0.950749 | 0.932741618 to 0.96910373 |
| 1950 to 1959 | APMP | Male | DALYs | 0.926606 | 0.906991864 to 0.946645228 |
| 1955 to 1964 | APMP | Male | DALYs | 0.816232 | 0.794672778 to 0.838377058 |
| 1960 to 1969 | APMP | Male | DALYs | 0.804335 | 0.7754745 to 0.834268605 |
| 1890 to 1899 | APMP | Female | DALYs | 0.49386 | 0.343893608 to 0.709225343 |
| 1895 to 1904 | APMP | Female | DALYs | 0.544379 | 0.467837163 to 0.633444404 |
| 1900 to 1909 | APMP | Female | DALYs | 0.586024 | 0.542517484 to 0.63302031 |
| 1905 to 1914 | APMP | Female | DALYs | 0.650913 | 0.621520127 to 0.681696348 |
| 1910 to 1919 | APMP | Female | DALYs | 0.741389 | 0.717763436 to 0.7657924 |
| 1915 to 1924 | APMP | Female | DALYs | 0.828852 | 0.808390081 to 0.849832561 |
| 1920 to 1929 | APMP | Female | DALYs | 0.927523 | 0.908508553 to 0.94693518 |

| | | | | | |
|---|---|---|---|---|---|
| 1925 to 1934 | APMP | Female | DALYs | 1 | 1 to 1 |
| 1930 to 1939 | APMP | Female | DALYs | 1.045429 | 1.026535816 to 1.064669703 |
| 1935 to 1944 | APMP | Female | DALYs | 1.077377 | 1.057605326 to 1.0975173 |
| 1940 to 1949 | APMP | Female | DALYs | 1.18335 | 1.160806717 to 1.206331172 |
| 1945 to 1954 | APMP | Female | DALYs | 1.304064 | 1.277919727 to 1.330743838 |
| 1950 to 1959 | APMP | Female | DALYs | 1.380039 | 1.349009182 to 1.411783102 |
| 1955 to 1964 | APMP | Female | DALYs | 1.324626 | 1.288035326 to 1.36225611 |
| 1960 to 1969 | APMP | Female | DALYs | 1.424962 | 1.373319514 to 1.478546172 |
| 1890 to 1899 | HAP | Both | DALYs | 3.231878 | 1.541730456 to 6.774875937 |
| 1895 to 1904 | HAP | Both | DALYs | 2.639869 | 2.063345538 to 3.37747935 |
| 1900 to 1909 | HAP | Both | DALYs | 2.204816 | 1.977406921 to 2.458377881 |
| 1905 to 1914 | HAP | Both | DALYs | 1.822846 | 1.714127666 to 1.938459202 |
| 1910 to 1919 | HAP | Both | DALYs | 1.700152 | 1.635966177 to 1.766855633 |
| 1915 to 1924 | HAP | Both | DALYs | 1.459341 | 1.41981472 to 1.499966658 |
| 1920 to 1929 | HAP | Both | DALYs | 1.200546 | 1.174630944 to 1.227033214 |
| 1925 to 1934 | HAP | Both | DALYs | 1 | 1 to 1 |
| 1930 to 1939 | HAP | Both | DALYs | 0.814946 | 0.799761753 to 0.830417845 |
| 1935 to 1944 | HAP | Both | DALYs | 0.61994 | 0.608014951 to 0.632099804 |
| 1940 to 1949 | HAP | Both | DALYs | 0.491329 | 0.481185797 to 0.501686798 |
| 1945 to 1954 | HAP | Both | DALYs | 0.38479 | 0.376200439 to 0.393576346 |
| 1950 to 1959 | HAP | Both | DALYs | 0.301261 | 0.293605154 to 0.309117372 |
| 1955 to 1964 | HAP | Both | DALYs | 0.219832 | 0.212717456 to 0.227184844 |
| 1960 to 1969 | HAP | Both | DALYs | 0.182762 | 0.174365311 to 0.191563014 |
| 1890 to 1899 | HAP | Male | DALYs | 3.876511 | 1.28415558 to 11.70211538 |
| 1895 to 1904 | HAP | Male | DALYs | 2.970667 | 2.177450703 to 4.052841114 |
| 1900 to 1909 | HAP | Male | DALYs | 2.351016 | 2.059029083 to 2.684408565 |
| 1905 to 1914 | HAP | Male | DALYs | 1.943113 | 1.80599688 to 2.090640199 |
| 1910 to 1919 | HAP | Male | DALYs | 1.796554 | 1.718555341 to 1.878093655 |
| 1915 to 1924 | HAP | Male | DALYs | 1.52086 | 1.474485912 to 1.568692224 |

| | | | | | |
|---|---|---|---|---|---|
| 1920 to 1929 | HAP | Male | DALYs | 1.23074 | 1.201254753 to 1.260948969 |
| 1925 to 1934 | HAP | Male | DALYs | 1 | 1 to 1 |
| 1930 to 1939 | HAP | Male | DALYs | 0.804339 | 0.787833285 to 0.821190191 |
| 1935 to 1944 | HAP | Male | DALYs | 0.607703 | 0.594833651 to 0.620851047 |
| 1940 to 1949 | HAP | Male | DALYs | 0.475287 | 0.464479613 to 0.486345147 |
| 1945 to 1954 | HAP | Male | DALYs | 0.370516 | 0.361402677 to 0.379858907 |
| 1950 to 1959 | HAP | Male | DALYs | 0.29023 | 0.282102616 to 0.298592472 |
| 1955 to 1964 | HAP | Male | DALYs | 0.211365 | 0.203821775 to 0.21918668 |
| 1960 to 1969 | HAP | Male | DALYs | 0.171511 | 0.162687347 to 0.180813602 |
| 1890 to 1899 | HAP | Female | DALYs | 2.990568 | 1.749370897 to 5.112406989 |
| 1895 to 1904 | HAP | Female | DALYs | 2.475441 | 2.016114482 to 3.039413892 |
| 1900 to 1909 | HAP | Female | DALYs | 2.177674 | 1.98111757 to 2.393731265 |
| 1905 to 1914 | HAP | Female | DALYs | 1.791423 | 1.695153535 to 1.893158865 |
| 1910 to 1919 | HAP | Female | DALYs | 1.659937 | 1.600770852 to 1.7212905 |
| 1915 to 1924 | HAP | Female | DALYs | 1.425936 | 1.388016402 to 1.464891623 |
| 1920 to 1929 | HAP | Female | DALYs | 1.18024 | 1.154496976 to 1.20655733 |
| 1925 to 1934 | HAP | Female | DALYs | 1 | 1 to 1 |
| 1930 to 1939 | HAP | Female | DALYs | 0.83528 | 0.819286064 to 0.851586938 |
| 1935 to 1944 | HAP | Female | DALYs | 0.644708 | 0.631943563 to 0.657730347 |
| 1940 to 1949 | HAP | Female | DALYs | 0.522816 | 0.511715047 to 0.534158523 |
| 1945 to 1954 | HAP | Female | DALYs | 0.417915 | 0.408330888 to 0.427724702 |
| 1950 to 1959 | HAP | Female | DALYs | 0.329529 | 0.320930798 to 0.338358157 |
| 1955 to 1964 | HAP | Female | DALYs | 0.243374 | 0.235311753 to 0.251712907 |
| 1960 to 1969 | HAP | Female | DALYs | 0.213691 | 0.203961143 to 0.223885767 |
| 1890 to 1899 | PMP | Both | DALYs | 0.903944 | 0.593527605 to 1.376707759 |
| 1895 to 1904 | PMP | Both | DALYs | 0.899378 | 0.770767301 to 1.049448598 |
| 1900 to 1909 | PMP | Both | DALYs | 0.901231 | 0.83954776 to 0.967446448 |
| 1905 to 1914 | PMP | Both | DALYs | 0.921773 | 0.885671909 to 0.959346162 |
| 1910 to 1919 | PMP | Both | DALYs | 0.983263 | 0.958073367 to 1.009114765 |

| | | | | | |
|---|---|---|---|---|---|
| 1915 to 1924 | PMP | Both | DALYs | 1.004259 | 0.985403395 to 1.023476403 |
| 1920 to 1929 | PMP | Both | DALYs | 1.01605 | 1.000776945 to 1.031556558 |
| 1925 to 1934 | PMP | Both | DALYs | 1 | 1 to 1 |
| 1930 to 1939 | PMP | Both | DALYs | 0.937134 | 0.925041454 to 0.94938495 |
| 1935 to 1944 | PMP | Both | DALYs | 0.83727 | 0.826325622 to 0.848360307 |
| 1940 to 1949 | PMP | Both | DALYs | 0.770852 | 0.760327555 to 0.781521749 |
| 1945 to 1954 | PMP | Both | DALYs | 0.718863 | 0.708533754 to 0.729342681 |
| 1950 to 1959 | PMP | Both | DALYs | 0.648556 | 0.638093764 to 0.659190595 |
| 1955 to 1964 | PMP | Both | DALYs | 0.530685 | 0.519935851 to 0.541656183 |
| 1960 to 1969 | PMP | Both | DALYs | 0.485899 | 0.472420755 to 0.499761194 |
| 1890 to 1899 | PMP | Male | DALYs | 1.123765 | 0.635202625 to 1.988102009 |
| 1895 to 1904 | PMP | Male | DALYs | 1.009655 | 0.836304095 to 1.218937477 |
| 1900 to 1909 | PMP | Male | DALYs | 0.967646 | 0.890912924 to 1.050988828 |
| 1905 to 1914 | PMP | Male | DALYs | 0.987771 | 0.944176158 to 1.033378435 |
| 1910 to 1919 | PMP | Male | DALYs | 1.029281 | 1.00028174 to 1.059121893 |
| 1915 to 1924 | PMP | Male | DALYs | 1.033825 | 1.01288647 to 1.055197026 |
| 1920 to 1929 | PMP | Male | DALYs | 1.036277 | 1.019732108 to 1.053089973 |
| 1925 to 1934 | PMP | Male | DALYs | 1 | 1 to 1 |
| 1930 to 1939 | PMP | Male | DALYs | 0.927008 | 0.914422614 to 0.939767124 |
| 1935 to 1944 | PMP | Male | DALYs | 0.822232 | 0.810944629 to 0.833676402 |
| 1940 to 1949 | PMP | Male | DALYs | 0.742666 | 0.732022252 to 0.753465325 |
| 1945 to 1954 | PMP | Male | DALYs | 0.69062 | 0.680215757 to 0.701183586 |
| 1950 to 1959 | PMP | Male | DALYs | 0.620462 | 0.609969136 to 0.631135861 |
| 1955 to 1964 | PMP | Male | DALYs | 0.504067 | 0.493332016 to 0.515035971 |
| 1960 to 1969 | PMP | Male | DALYs | 0.454831 | 0.441431743 to 0.468636766 |
| 1890 to 1899 | PMP | Female | DALYs | 0.833913 | 0.623343008 to 1.115614983 |
| 1895 to 1904 | PMP | Female | DALYs | 0.863391 | 0.767229442 to 0.971605503 |
| 1900 to 1909 | PMP | Female | DALYs | 0.896321 | 0.846081489 to 0.949544104 |
| 1905 to 1914 | PMP | Female | DALYs | 0.911771 | 0.881152924 to 0.943452124 |

| | | | | | |
|---|---|---|---|---|---|
| 1910 to 1919 | PMP | Female | DALYs | 0.986297 | 0.963626251 to 1.009501156 |
| 1915 to 1924 | PMP | Female | DALYs | 1.012642 | 0.994882788 to 1.030717269 |
| 1920 to 1929 | PMP | Female | DALYs | 1.013345 | 0.998609055 to 1.028299403 |
| 1925 to 1934 | PMP | Female | DALYs | 1 | 1 to 1 |
| 1930 to 1939 | PMP | Female | DALYs | 0.958884 | 0.9466225 to 0.97130465 |
| 1935 to 1944 | PMP | Female | DALYs | 0.873367 | 0.861989972 to 0.884893497 |
| 1940 to 1949 | PMP | Female | DALYs | 0.839574 | 0.828135701 to 0.851170638 |
| 1945 to 1954 | PMP | Female | DALYs | 0.801607 | 0.790071804 to 0.813311075 |
| 1950 to 1959 | PMP | Female | DALYs | 0.74035 | 0.728381879 to 0.752514512 |
| 1955 to 1964 | PMP | Female | DALYs | 0.62338 | 0.610832225 to 0.636184915 |
| 1960 to 1969 | PMP | Female | DALYs | 0.596417 | 0.580374258 to 0.612903036 |

Abbreviation: PMP, particulate matter pollution; APMP, ambient particulate matter pollution; HAP, household air pollution from solid fuels; DALY, Disability-adjusted life years.

Supplement table 6: The trends in the lung cancer burden of death attributable to PMP, APMP and HAP by sex in different SDI regions from 1990 to 2021.

| Location | Risk | Sex | Death Number in 2021, | Change of death numbers 1990-2021, % | Rate of death in 2021, per 100000 | Percent change of death 1990-2021, % | Rate of death in 2021, per 100000 | Percent change of death 1990-2021, % | Net drift of mortality, % per year |
|---|---|---|---|---|---|---|---|---|---|
| High-middle SDI | PMP | Male | 83858 (51494 to 121301) | 21.19 (-10.68 to 62.3) | 12.9 (7.9 to 18.6) | -1.71 (-27.56 to 31.63) | 9.5 (5.8 to 13.6) | -40.88 (-56.38 to -21.16) | -1.91 (-2.05 to -1.77) |
| High-middle SDI | PMP | Female | 40738 (25257 to 58268) | 79.59 (38.28 to 131.1) | 6.3 (3.9 to 8.9) | 47.28 (13.4 to 89.53) | 3.7 (2.3 to 5.3) | -9.06 (-29.86 to 16.91) | -0.82 (-0.91 to -0.72) |
| High-middle SDI | PMP | Both | 124596(79136 to 176647) | 35.61 (8.05 to 72.69) | 9.6 (6.1 to 13.5) | 10.6 (-11.88 to 40.84) | 6.2 (3.9 to 8.8) | -31.92 (-45.81 to -13.32) | -1.52 (-1.65 to -1.39) |
| High SDI | HAP | Male | 123 (0 to 1165) | -94.98 (-99.98 to -82.74) | 0 (0 to 0.2) | -96.01 (-99.98 to -86.29) | 0 (0 to 0.1) | -97.51 (-99.99 to -91.43) | -13.45 (-14.72 to -12.17) |
| High SDI | HAP | Female | 923 (0 to 833) | -92.37 (-99.97 to -69.4) | 0 (0 to 0.2) | -93.79 (-99.98 to -75.1) | 0 (0 to 0.1) | -95.87 (-99.99 to -83.29) | -12.2 (-13.51 to -10.87) |
| High SDI | HAP | Both | 216 (0.3 to 2012) | -94.11 (-99.98 to -78.05) | 0 (0 to 0.2) | -95.27 (-99.98 to -82.35) | 0 (0 to 0.1) | -96.94 (-99.99 to -88.54) | -12.75 (-13.74 to -11.75) |
| Low-middle SDI | PMP | Male | 27998 (18225 to 38194) | 89.36 (45.02 to 133.81) | 2.9 (1.9 to 4) | 15.93 (-11.21 to 43.15) | 4.1 (2.7 to 5.6) | -15.64 (-35.11 to 3.93) | -0.45 (-0.56 to -0.34) |
| Low-middle SDI | PMP | Female | 12602 (7997 to 17415) | 162.07 (112.88 to 220.87) | 1.3 (0.8 to 1.8) | 56.4 (27.05 to 91.49) | 1.7 (1.1 to 2.3) | 4.96 (-14.57 to 28.82) | 0.13 (-0.02 to 0.29) |
| Low-middle SDI | PMP | Both | 40600 (26072 to 54813) | 107.2 (65.28 to 147.14) | 2.1 (1.4 to 2.9) | 25.26 (-0.08 to 49.41) | 2.8 (1.8 to 3.8) | -12.7 (-30.17 to 4.18) | -0.36 (-0.45 to -0.27) |
| High-middle SDI | HAP | Male | 4887 (244 to 25335) | -82.88 (-98.76 to -36.49) | 0.7 (0 to 3.9) | -86.11 (-98.99 to -48.49) | 0.5 (0 to 2.8) | -91.76 (-99.41 to -69.19) | -8.59 (-8.74 to -8.44) |

| | | | | | | | | | | |
|---|---|---|---|---|---|---|---|---|---|---|
| High-middle SDI | HAP | Female | 3879 (224 to 19847) | -71.48 (-97.77 to 9.76) | 0.6 (0 to 3) | -76.61 (-98.17 to -9.98) | 0.4 (0 to 1.8) | -85.57 (-98.87 to -44.26) | -7.23 (-7.39 to -7.08) |
| High-middle SDI | HAP | Both | 8765 (476 to 44605) | -79.2 (-98.41 to -26.25) | 0.7 (0 to 3.4) | -83.04 (-98.7 to -39.85) | 0.4 (0 to 2.2) | -89.62 (-99.21 to -63.02) | -8.01 (-8.15 to -7.87) |
| Low SDI | HAP | Male | 6358 (4017 to 9028) | 70.22 (37.81 to 119.31) | 1.1 (0.7 to 1.6) | -23.18 (-37.8 to -1.02) | 2.7 (1.7 to 3.8) | -18.89 (-33.85 to 3.31) | -0.78 (-1.03 to -0.54) |
| Low SDI | HAP | Female | 2935 (1849 to 4084) | 159.93 (86.92 to 238.03) | 0.5 (0.3 to 0.7) | 15.92 (-16.64 to 50.75) | 1.2 (0.7 to 1.6) | 13.49 (-17.27 to 47.6) | 0.25 (-0.12 to 0.63) |
| Low SDI | HAP | Both | 9294 (5854 to 13002) | 91.04 (58.96 to 136.55) | 0.8 (0.5 to 1.2) | -14.29 (-28.68 to 6.12) | 1.9 (1.2 to 2.6) | -12.96 (-27.44 to 7.63) | -0.5 (-0.72 to -0.28) |
| Middle SDI | HAP | Male | 21589 (4270 to 62859) | -46.12 (-87.14 to 34.57) | 1.8 (0.3 to 5.1) | -61.68 (-90.85 to -4.29) | 1.8 (0.3 to 5.1) | -78.7 (-95 to -46.93) | -4.97 (-5.09 to -4.86) |
| Middle SDI | HAP | Female | 12749 (2775 to 32652) | -34.38 (-83.35 to 48.93) | 1 (0.2 to 2.7) | -54.33 (-88.41 to 3.65) | 0.9 (0.2 to 2.4) | -75.47 (-93.8 to -44.52) | -5.02 (-5.12 to -4.92) |
| Middle SDI | HAP | Both | 34338 (7201 to 96084) | -42.29 (-85.11 to 40.67) | 1.4 (0.3 to 3.9) | -59.39 (-89.52 to -1.02) | 1.3 (0.3 to 3.6) | -77.76 (-94.3 to -45.95) | -5 (-5.09 to -4.91) |
| Low-middle SDI | HAP | Male | 15546 (8450 to 25001) | 34.47 (-8.29 to 83) | 1.6 (0.9 to 2.6) | -17.67 (-43.85 to 12.04) | 2.3 (1.2 to 3.7) | -40.09 (-59.09 to -18.33) | -1.67 (-1.8 to -1.54) |
| Low-middle SDI | HAP | Female | 8267 (4734 to 12758) | 105.45 (43.53 to 171.59) | 0.9 (0.5 to 1.3) | 22.61 (-14.34 to 62.08) | 1.1 (0.6 to 1.7) | -17.53 (-42.15 to 9.13) | -0.73 (-0.9 to -0.56) |
| Low-middle SDI | HAP | Both | 23813 (13505 to 37394) | 52.8 (8.17 to 99.75) | 1.2 (0.7 to 1.9) | -7.63 (-34.61 to 20.76) | 1.7 (0.9 to 2.6) | -35.51 (-54.06 to -15.75) | -1.44 (-1.54 to -1.34) |
| High SDI | PMP | Male | 30799 (17556 to 44505) | -23.23 (-43.86 to 7.22) | 5.6 (3.2 to 8.2) | -39.03 (-55.42 to -14.85) | 3.2 (1.8 to 4.6) | -62.96 (-72.9 to -48.1) | -3.39 (-3.5 to -3.29) |
| High SDI | PMP | Female | 17730 (10272 to 26194) | 15.27 (-15.79 to 60.14) | 3.2 (1.9 to 4.8) | -6.22 (-31.49 to 30.29) | 1.5 (0.9 to 2.2) | -39.03 (-55.03 to -15.19) | -1.88 (-2.01 to -1.74) |

| | | | | | | | | | |
|---|---|---|---|---|---|---|---|---|---|
| High SDI | PMP | Both | 48529 (27700 to 70716) | -12.56 (-35.77 to 21.08) | 4.4 (2.5 to 6.5) | -29.71 (-48.37 to -2.66) | 2.2 (1.3 to 3.2) | -55.31 (-67 to -37.92) | -2.76 (-2.89 to -2.64) |
| Low SDI | PMP | Male | 8102 (5214 to 11237) | 83.8 (48.43 to 134.49) | 1.4 (0.9 to 2) | -17.05 (-33.01 to 5.83) | 3.4 (2.2 to 4.7) | -12.64 (-29.32 to 10.04) | -0.43 (-0.66 to -0.2) |
| Low SDI | PMP | Female | 3446 (2195 to 4686) | 173.68 (101.69 to 252.02) | 0.6 (0.4 to 0.8) | 22.05 (-10.05 to 56.99) | 1.4 (0.9 to 1.8) | 19.46 (-11.57 to 54.57) | 0.47 (0.11 to 0.83) |
| Low SDI | PMP | Both | 11548 (7446 to 15728) | 103.77 (70.3 to 149.47) | 1 (0.7 to 1.4) | -8.58 (-23.6 to 11.92) | 2.4 (1.5 to 3.2) | -7.32 (-22.43 to 13.03) | -0.24 (-0.43 to -0.05) |
| Middle SDI | PMP | Male | 101453 (61131 to 144030) | 81.87 (28.52 to 141.38) | 8.2 (5 to 11.7) | 29.35 (-8.6 to 71.67) | 8.3 (5 to 11.8) | -28 (-48.91 to -5.08) | -1.18 (-1.29 to -1.07) |
| Middle SDI | PMP | Female | 47181 (30188 to 66126) | 92.15 (48.71 to 142.16) | 3.9 (2.5 to 5.4) | 33.73 (3.5 to 68.53) | 3.4 (2.2 to 4.8) | -28.31 (-44.29 to -10.18) | -1.68 (-1.75 to -1.6) |
| Middle SDI | PMP | Both | 148634 (91549 to 208339) | 85.01 (43.28 to 133.21) | 6.1 (3.7 to 8.5) | 30.18 (0.82 to 64.1) | 5.7 (3.5 to 8) | -28.8 (-44.58 to -10.65) | -1.36 (-1.45 to -1.26) |
| High SDI | APMP | Male | 30673 (17503 to 44434) | -18.58 (-41.53 to 15.2) | 5.6 (3.2 to 8.1) | -35.33 (-53.56 to -8.51) | 3.1 (1.8 to 4.5) | -60.76 (-71.82 to -44.37) | -3.15 (-3.27 to -3.04) |
| High SDI | APMP | Female | 17634 (10232 to 26132) | 24.53 (-9.47 to 79.53) | 3.2 (1.9 to 4.8) | 1.32 (-26.34 to 46.06) | 1.5 (0.9 to 2.2) | -34 (-52.01 to -4.36) | -1.56 (-1.7 to -1.41) |
| High SDI | APMP | Both | 48307 (27621 to 69673) | -6.8 (-32.68 to 31.7) | 4.4 (2.5 to 6.4) | -25.08 (-45.88 to 5.88) | 2.2 (1.3 to 3.2) | -52.33 (-65.48 to -32.65) | -2.51 (-2.64 to -2.37) |
| High-middle SDI | APMP | Male | 78943 (48863 to 113110) | 94.24 (34.74 to 179.2) | 12.1 (7.5 to 17.3) | 57.54 (9.28 to 126.44) | 8.9 (5.5 to 12.7) | -4.72 (-33.32 to 36.58) | 0.19 (0 to 0.37) |
| High-middle SDI | APMP | Female | 36839 (21109 to 54148) | 305.72 (186.93 to 473.85) | 5.7 (3.2 to 8.3) | 232.74 (135.31 to 370.62) | 3.3 (1.9 to 4.9) | 105.68 (45.52 to 191.6) | 2.8 (2.68 to 2.93) |
| High-middle SDI | APMP | Both | 115781 (70122 to 161752) | 132.86 (70.4 to 232.58) | 8.9 (5.4 to 12.4) | 89.92 (38.97 to 171.25) | 5.8 (3.5 to 8.1) | 17.33 (-13.91 to 67.73) | 0.92 (0.74 to 1.09) |
| Low SDI | APMP | Male | 1743 (1052 to 268) | 159.24 (83.42 to 282.5) | 0.3 (0.2 to 0.5) | 17 (-17.22 to 72.63) | 0.7 (0.4 to 1.1) | 21.54 (-13.67 to 80.68) | 1.21 (0.44 to 1.98) |

| | | | | | | | | | |
|---|---|---|---|---|---|---|---|---|---|
| Low SDI | APMP | Female | 510 (297 to 779) | 293.5 (154.16 to 496.08) | 0.1 (0.1 to 0.1) | 75.48 (13.34 to 165.83) | 0.2 (0.1 to 0.3) | 71.26 (11.71 to 162.3) | 1.88 (0.84 to 2.94) |
| Low SDI | APMP | Both | 2253 (1340 to 3534) | 180.94 (101.11 to 301.72) | 0.2 (0.1 to 0.3) | 26.04 (-9.77 to 80.23) | 0.5 (0.3 to 0.7) | 26.48 (-9.26 to 81.19) | 1.34 (0.62 to 2.07) |
| Low-middle SDI | APMP | Male | 12445 (7252 to 18847) | 286.14 (166.54 to 439.52) | 1.3 (0.8 to 2) | 136.41 (63.18 to 230.31) | 1.8 (1.1 to 2.8) | 71.38 (18.87 to 138.9) | 2.18 (1.97 to 2.39) |
| Low-middle SDI | APMP | Female | 4333 (2419 to 6783) | 452.54 (282 to 651.75) | 0.5 (0.3 to 0.7) | 229.75 (127.97 to 348.63) | 0.6 (0.3 to 0.9) | 119.01 (51.4 to 199.74) | 2.84 (2.51 to 3.16) |
| Low-middle SDI | APMP | Both | 16778 (9819 to 25273) | 318.7 (194.79 to 468.78) | 0.9 (0.5 to 1.3) | 153.13 (78.21 to 243.86) | 1.2 (0.7 to 1.8) | 75.4 (23.67 to 137.52) | 2.23 (2.06 to 2.41) |
| Middle SDI | APMP | Male | 79819 (45208 to 114745) | 408.18 (213.25 to 650.41) | 6.5 (3.7 to 9.3) | 261.42 (122.79 to 433.7) | 6.6 (3.7 to 9.4) | 97.55 (23.14 to 188.91) | 2.52 (2.41 to 2.64) |
| Middle SDI | APMP | Female | 34411 (18520 to 50837) | 571.83 (332.74 to 909.82) | 2.8 (1.5 to 4.2) | 367.57 (201.17 to 602.8) | 2.5 (1.3 to 3.7) | 147.02 (60.37 to 267.85) | 2.86 (2.73 to 2.99) |
| Middle SDI | APMP | Both | 114230 (65022 to 163143) | 448.42 (249.14 to 688.79) | 4.7 (2.7 to 6.7) | 285.9 (145.67 to 455.03) | 4.4 (2.5 to 6.2) | 108.37 (33.63 to 196.94) | 2.61 (2.5 to 2.71) |

The all-age mortality is equivalent to the crude DALY rate.

[a] The parentheses accompanying all Global Burden of Disease health estimates represent 95% uncertainty intervals, while the parentheses for net drift indicate 95% confidence intervals.

[b] The net drifts are estimates derived from the age–period–cohort model and signify the overall annual percentage change in DALY, encompassing the effects from calendar time and successive birth cohorts.

Abbreviation: PMP, particulate matter pollution; APMP, ambient particulate matter pollution; HAP, household air pollution from solid fuels; DALY, Disability-adjusted life years; SDI, socio-demographic indexes.

Supplement table 7: The trends in the lung cancer burden of DALYs attributable to PMP, APMP and HAP by sex in different SDI regions from 1990 to 2021

| Location | Risk | Sex | DALY Number in 2021 | Change of DALY numbers 1990-2021, % | Rate of DALY in 2021, per 100000 | Percent change of DALY 1990-2021, % | Rate of DALY in 2021, per 100000 | Percent change of DALY 1990-2021, % | Net drift of DALY, % per year |
|---|---|---|---|---|---|---|---|---|---|
| High SDI | HAP | Male | 2761.5 (4.7 to 26217.8) | -95.8 (-99.98 to -85.5) | 0.5 (0 to 4.8) | -96.66 (-99.98 to -88.48) | 0.3 (0 to 2.8) | -97.8 (-99.99 to -92.42) | -13.09 (-13.26 to -12.93) |
| High SDI | HAP | Female | 1983.5 (3 to 17783) | -93.59 (-99.98 to -73.74) | 0.4 (0 to 3.2) | -94.78 (-99.98 to -78.64) | 0.2 (0 to 1.7) | -96.41 (-99.99 to -85.25) | -11.89 (-12.07 to -11.72) |
| High SDI | HAP | Both | 4745.1 (7.7 to 44238.7) | -95.09 (-99.98 to -81.49) | 0.4 (0 to 4) | -96.05 (-99.98 to -85.12) | 0.2 (0 to 2.3) | -97.34 (-99.99 to -89.95) | -12.56 (-12.71 to -12.42) |
| Low-middle SDI | HAP | Male | 426835.9 (233539.2 to 688510.4) | 30.19 (-11.03 to 76.44) | 44.2 (24.2 to 71.3) | -20.29 (-45.53 to 8.02) | 57.7 (31.5 to 92.9) | -40.9 (-59.59 to -19.68) | -1.68 (-1.78 to -1.57) |
| Low-middle SDI | HAP | Female | 226059.6 (130458.3 to 347390.9) | 92.92 (34.79 to 154.89) | 23.7 (13.7 to 36.4) | 15.13 (-19.56 to 52.12) | 28.6 (16.4 to 43.8) | -19.61 (-43.67 to 6.24) | -0.73 (-0.82 to -0.65) |
| Low-middle SDI | HAP | Both | 652895.5 (372150 to 1019359.7) | 46.71 (3.97 to 92.06) | 34 (19.4 to 53.1) | -11.31 (-37.14 to 16.11) | 42.6 (24.3 to 66.8) | -36.49 (-55.02 to -16.76) | -1.44 (-1.51 to -1.37) |
| High-middle SDI | HAP | Male | 117379.4 (5983.9 to 604222.4) | -85.25 (-98.89 to -46.08) | 18 (0.9 to 92.6) | -88.04 (-99.1 to -56.27) | 12.7 (0.6 to 65.4) | -92.57 (-99.45 to -72.7) | -8.59 (-8.77 to -8.41) |
| High-middle SDI | HAP | Female | 88722.8 (5150.4 to 449911.2) | -75.87 (-98.1 to -6.09) | 13.6 (0.8 to 69.1) | -80.21 (-98.44 to -22.98) | 8.3 (0.5 to 42.2) | -87.37 (-99.01 to -50.57) | -7.22 (-7.35 to -7.1) |
| High-middle SDI | HAP | Both | 206102.2 (11336.7 to 1050686.1) | -82.28 (-98.61 to -38.07) | 15.8 (0.9 to 80.6) | -85.55 (-98.87 to -49.49) | 10.3 (0.6 to 52.7) | -90.82 (-99.28 to -67.86) | -8.01 (-8.16 to -7.87) |
| Low SDI | HAP | Male | 178495.4 (111416 to 255290.1) | 68.78 (36.8 to 121.1) | 31.9 (19.9 to 45.7) | -23.83 (-38.26 to -0.21) | 66.3 (41.7 to 94.1) | -21.68 (-36.53 to 1.45) | -0.77 (-0.91 to -0.63) |

| | | | | | | | | | |
|---|---|---|---|---|---|---|---|---|---|
| Low SDI | HAP | Female | 82841.2 (51864.8 to 116036.5) | 152.79 (80.04 to 226.74) | 14.8 (9.3 to 20.8) | 12.73 (-19.71 to 45.71) | 29.3 (18.4 to 40.9) | 9.1 (-21.78 to 41.52) | 0.26 (0.15 to 0.38) |
| Low SDI | HAP | Both | 261336.6 (163720.9 to 369459.1) | 88.66 (56.77 to 133.71) | 23.4 (14.7 to 33.1) | -15.36 (-29.67 to 4.85) | 47.4 (29.7 to 66.6) | -15.82 (-29.99 to 4.41) | -0.52 (-0.65 to -0.39) |
| Middle SDI | HAP | Male | 533829.2 (108280.8 to 1546021.3) | -52.81 (-88.36 to 17.09) | 43.3 (8.8 to 125.4) | -66.44 (-91.72 to -16.73) | 40.2 (8.1 to 116.4) | -80.55 (-95.26 to -51.67) | -4.97 (-5.12 to -4.82) |
| Middle SDI | HAP | Female | 305967.7 (68354.3 to 771976.9) | -43.38 (-85.21 to 28.89) | 25.2 (5.6 to 63.5) | -60.59 (-89.71 to -10.3) | 21.2 (4.7 to 53.5) | -77.7 (-94.19 to -49.39) | -5.02 (-5.1 to -4.93) |
| Middle SDI | HAP | Both | 839796.9 (180687.1 to 2339653.3) | -49.76 (-86.63 to 21.95) | 34.3 (7.4 to 95.6) | -64.65 (-90.59 to -14.19) | 30.2 (6.5 to 84.2) | -79.77 (-94.65 to -50.87) | -5 (-5.11 to -4.88) |
| High-middle SDI | PMP | Male | 1983069.2 (1222090.3 to 2884322.9) | 2.85 (-24.47 to 38.42) | 303.9 (187.3 to 442) | -16.58 (-38.74 to 12.27) | 215.3 (132.7 to 312.3) | -47.76 (-61.71 to -29.92) | -1.91 (-2.1 to -1.71) |
| High-middle SDI | PMP | Female | 920721.6 (568420.8 to 1319698.5) | 54.06 (17.37 to 99.9) | 141.3 (87.3 to 202.6) | 26.35 (-3.74 to 63.94) | 86.6 (53.3 to 124.3) | -18.94 (-38.42 to 5.38) | -0.81 (-0.92 to -0.71) |
| High-middle SDI | PMP | Both | 2903790.9 (1851051.1 to 4099487) | 14.97 (-8.76 to 47.88) | 222.7 (141.9 to 314.4) | -6.23 (-25.59 to 20.61) | 145.6 (92.8 to 205.8) | -40.22 (-52.54 to -23.14) | -1.51 (-1.67 to -1.35) |
| High SDI | PMP | Male | 638400 (367667.8 to 912187.2) | -35.77 (-52.4 to -10.76) | 117 (67.4 to 167.2) | -48.99 (-62.19 to -29.13) | 68.2 (39.3 to 97.3) | -66.92 (-75.45 to -53.98) | -3.39 (-3.47 to -3.3) |
| High SDI | PMP | Female | 351368.2 (204359.8 to 516811.5) | -3.37 (-28.49 to 34.46) | 64.1 (37.3 to 94.2) | -21.38 (-41.82 to 9.4) | 33.3 (19.4 to 48.8) | -45.7 (-59.98 to -24.39) | -1.85 (-1.95 to -1.75) |
| High SDI | PMP | Both | 989768.2 (561814.8 to 1421096.7) | -27.09 (-45.84 to 0.44) | 90.5 (51.4 to 129.9) | -41.39 (-56.46 to -19.25) | 49.6 (28.3 to 70.8) | -60.56 (-70.66 to -45.58) | -2.76 (-2.85 to -2.66) |

| | | | | | | | | | |
|---|---|---|---|---|---|---|---|---|---|
| Low-middle SDI | PMP | Male | 767961 (502775.3 to 1049572.4) | 83.17 (39.81 to 126.95) | 79.5 (52.1 to 108.7) | 12.14 (-14.4 to 38.94) | 103.9 (67.8 to 141.8) | -16.81 (-36.34 to 2.86) | -0.45 (-0.54 to -0.36) |
| Low-middle SDI | PMP | Female | 345019 (218118.1 to 479363.6) | 146.51 (98.47 to 203.23) | 36.1 (22.8 to 50.2) | 47.11 (18.44 to 80.96) | 43.6 (27.6 to 60.4) | 2.65 (-16.91 to 26) | 0.12 (0.04 to 0.21) |
| Low-middle SDI | PMP | Both | 1112980 (716547 to 1503839.4) | 99.02 (58.52 to 137.33) | 57.9 (37.3 to 78.3) | 20.32 (-4.17 to 43.48) | 72.7 (46.7 to 98.2) | -13.87 (-31.4 to 2.87) | -0.36 (-0.43 to -0.29) |
| High SDI | APMP | Male | 635559.4 (366264 to 909876.3) | -31.52 (-50.22 to -3.7) | 116.5 (67.1 to 166.7) | -45.61 (-60.46 to -23.52) | 67.9 (39.2 to 97) | -64.75 (-74.37 to -50.32) | -3.14 (-3.23 to -3.05) |
| High SDI | APMP | Female | 349331.6 (203477.1 to 514681.1) | 5.02 (-23.96 to 51.84) | 63.7 (37.1 to 93.9) | -14.56 (-38.13 to 23.54) | 33.1 (19.3 to 48.6) | -40.85 (-57.46 to -14.91) | -1.53 (-1.64 to -1.42) |
| High SDI | APMP | Both | 984891 (560115.7 to 1417157.6) | -21.88 (-43.13 to 10.11) | 90 (51.2 to 129.5) | -37.2 (-54.28 to -11.48) | 49.4 (28.2 to 70.7) | -57.7 (-69.18 to -40.4) | -2.49 (-2.59 to -2.39) |
| Middle SDI | PMP | Male | 2472521.5 (1500172.5 to 3512045.9) | 57.46 (11.23 to 109.96) | 200.5 (121.6 to 284.8) | 11.98 (-20.89 to 49.33) | 187.1 (113.2 to 265.6) | -34.91 (-54 to -13.7) | -1.18 (-1.34 to -1.02) |
| Middle SDI | PMP | Female | 1123044.2 (719903.2 to 1593409.4) | 64.87 (27.88 to 109.84) | 92.4 (59.2 to 131.1) | 14.74 (-11 to 46.04) | 78 (49.9 to 110.3) | -35.08 (-49.59 to -17.64) | -1.68 (-1.77 to -1.59) |
| Middle SDI | PMP | Both | 3595565.7 (2244128.4 to 5032675.9) | 59.7 (22.97 to 103.53) | 146.8 (91.7 to 205.5) | 12.37 (-13.48 to 43.21) | 129.7 (80.7 to 181.5) | -35.65 (-50.33 to -18.44) | -1.35 (-1.48 to -1.23) |
| Low SDI | PMP | Male | 227279.7 (144869.1 to 316880.6) | 82.38 (46.48 to 134.73) | 40.7 (25.9 to 56.7) | -17.69 (-33.89 to 5.94) | 84.4 (54.3 to 117.3) | -15.46 (-31.74 to 7.96) | -0.44 (-0.58 to -0.31) |
| Low SDI | PMP | Female | 97296.3 (61379.8 to 133377.2) | 166.23 (94.36 to 241.96) | 17.4 (11 to 23.9) | 18.73 (-13.32 to 52.5) | 34.4 (21.8 to 46.9) | 14.85 (-15.57 to 47.67) | 0.49 (0.38 to 0.61) |

| | | | | | | | | | |
|---|---|---|---|---|---|---|---|---|---|
| Low SDI | PMP | Both | 324576 (207272.4 to 444105.1) | 101.39 (68.28 to 147.54) | 29 (18.5 to 39.7) | -9.65 (-24.5 to 11.06) | 58.8 (37.7 to 80.2) | -10.21 (-24.96 to 10.07) | -0.24 (-0.36 to -0.11) |
| Low-middle SDI | APMP | Male | 340950.1 (199016.7 to 518484.6) | 273.21 (157.28 to 422.2) | 35.3 (20.6 to 53.7) | 128.49 (57.52 to 219.71) | 46.2 (26.9 to 70) | 69.49 (17.2 to 137.44) | 2.17 (2.09 to 2.25) |
| Low-middle SDI | APMP | Female | 118893.3 (66460.2 to 185726.2) | 422.37 (264.25 to 607.53) | 12.4 (7 to 19.4) | 211.74 (117.38 to 322.24) | 15 (8.4 to 23.5) | 117.01 (51.21 to 195.02) | 2.86 (2.76 to 2.95) |
| Low-middle SDI | APMP | Both | 459843.4 (269679.8 to 694425.4) | 302.96 (183.91 to 448.19) | 23.9 (14 to 36.1) | 143.61 (71.64 to 231.41) | 30 (17.6 to 45.3) | 74.14 (22.52 to 136.82) | 2.23 (2.16 to 2.3) |
| Low SDI | APMP | Male | 48747.3 (29392 to 75046.7) | 158.59 (81.81 to 280.24) | 8.7 (5.3 to 13.4) | 16.7 (-17.95 to 71.61) | 18.1 (11 to 27.9) | 19.15 (-15.89 to 75.88) | 1.04 (0.88 to 1.2) |
| Low SDI | APMP | Female | 14444.4 (8394.4 to 22335.9) | 283.08 (144.44 to 482.58) | 2.6 (1.5 to 4) | 70.84 (9.01 to 159.81) | 5.1 (3 to 7.8) | 64.8 (5.98 to 149.96) | 2.11 (1.81 to 2.42) |
| Low SDI | APMP | Both | 63191.7 (37758.6 to 98994.4) | 179.34 (99.04 to 300.01) | 5.7 (3.4 to 8.9) | 25.32 (-10.7 to 79.46) | 11.5 (6.8 to 18) | 23.88 (-11.55 to 77.5) | 1.2 (1.06 to 1.34) |
| High-middle SDI | APMP | Male | 1864999.1 (1151199.2 to 2681048.3) | 64.77 (13.94 to 139.07) | 285.8 (176.4 to 410.9) | 33.64 (-7.59 to 93.89) | 202.5 (125.1 to 290.5) | -16 (-41.79 to 21.19) | 0.2 (-0.04 to 0.44) |
| High-middle SDI | APMP | Female | 831539.9 (477225.3 to 1221197.7) | 261.68 (153.36 to 421.04) | 127.6 (73.3 to 187.5) | 196.62 (107.79 to 327.31) | 78.2 (44.8 to 114.9) | 91.23 (34.01 to 174.7) | 2.83 (2.68 to 2.98) |
| High-middle SDI | APMP | Both | 2696539 (1645210 to 3789059.2) | 98.02 (44.17 to 186.15) | 206.8 (126.2 to 290.6) | 61.5 (17.58 to 133.37) | 135.2 (82.5 to 189.7) | 3.23 (-24.78 to 49.38) | 0.93 (0.71 to 1.16) |
| Middle SDI | APMP | Male | 1937609.1 (1101997.1 to 2784417.7) | 341.45 (170.88 to 555.55) | 157.1 (89.4 to 225.8) | 213.97 (92.66 to 366.23) | 146.9 (83.5 to 211.2) | 81.13 (12.06 to 167.49) | 2.53 (2.36 to 2.69) |

| | | | | | | | | | |
|---|---|---|---|---|---|---|---|---|---|
| Middle SDI | APMP | Female | 816568.7 (435850.4 to 1204969.4) | 480.11 (274.33 to 777.05) | 67.2 (35.9 to 99.1) | 303.74 (160.52 to 510.4) | 56.7 (30.3 to 83.7) | 127.08 (46.83 to 241.83) | 2.86 (2.75 to 2.97) |
| Middle SDI | APMP | Both | 2754177.8 (1567192.8 to 3932350.1) | 375.12 (201.47 to 585.27) | 112.5 (64 to 160.6) | 234.32 (112.13 to 382.19) | 99.4 (56.6 to 142) | 90.41 (21.33 to 173.97) | 2.61 (2.46 to 2.75) |

The all-age mortality is equivalent to the crude DALY rate.

[a] The parentheses accompanying all Global Burden of Disease health estimates represent 95% uncertainty intervals, while the parentheses for net drift indicate 95% confidence intervals.

[b] The net drifts are estimates derived from the age–period–cohort model and signify the overall annual percentage change in DALY, encompassing the effects from calendar time and successive birth cohorts.

Abbreviation: PMP, particulate matter pollution. AMPM, ambient particulate matter pollution. HAP, household air pollution from solid fuels. DALY, Disability-adjusted life years., SDI, socio-demographic indexes.

Supplement table 8: Global trends of lung cancer attributable to PMP, APMP and HAP in age-standardized death and DALYs rates (per 100,000 population) from 2019 to 2030 by predicted by BAPC models:

| Risk | Sex | measure | Year | Predicted Value | Bound of Predicted Value |
|---|---|---|---|---|---|
| PMP | Male | Deaths | 1990 | 18.6 | 18.5 to 18.6 |
| PMP | Male | Deaths | 1991 | 18.4 | 18.3 to 18.5 |
| PMP | Male | Deaths | 1992 | 18.2 | 18.1 to 18.3 |
| PMP | Male | Deaths | 1993 | 18.1 | 18.1 to 18.2 |
| PMP | Male | Deaths | 1994 | 18 | 17.9 to 18.1 |
| PMP | Male | Deaths | 1995 | 17.9 | 17.9 to 18 |
| PMP | Male | Deaths | 1996 | 17.7 | 17.6 to 17.8 |
| PMP | Male | Deaths | 1997 | 17.5 | 17.4 to 17.5 |
| PMP | Male | Deaths | 1998 | 17.3 | 17.2 to 17.4 |
| PMP | Male | Deaths | 1999 | 17.2 | 17.2 to 17.3 |
| PMP | Male | Deaths | 2000 | 17.4 | 17.3 to 17.4 |
| PMP | Male | Deaths | 2001 | 17.3 | 17.2 to 17.3 |
| PMP | Male | Deaths | 2002 | 17.1 | 17 to 17.2 |
| PMP | Male | Deaths | 2003 | 17 | 17 to 17.1 |
| PMP | Male | Deaths | 2004 | 16.9 | 16.9 to 17 |
| PMP | Male | Deaths | 2005 | 16.7 | 16.6 to 16.7 |
| PMP | Male | Deaths | 2006 | 16 | 15.9 to 16.1 |
| PMP | Male | Deaths | 2007 | 15.6 | 15.5 to 15.6 |
| PMP | Male | Deaths | 2008 | 15.4 | 15.3 to 15.4 |
| PMP | Male | Deaths | 2009 | 15.2 | 15.1 to 15.2 |
| PMP | Male | Deaths | 2010 | 15 | 14.9 to 15.1 |
| PMP | Male | Deaths | 2011 | 14.8 | 14.8 to 14.9 |
| PMP | Male | Deaths | 2012 | 14.6 | 14.6 to 14.7 |
| PMP | Male | Deaths | 2013 | 14.3 | 14.3 to 14.4 |
| PMP | Male | Deaths | 2014 | 14 | 13.9 to 14 |

| | | | | | |
|---|---|---|---|---|---|
| PMP | Male | Deaths | 2015 | 13.7 | 13.7 to 13.8 |
| PMP | Male | Deaths | 2016 | 13.3 | 13.3 to 13.4 |
| PMP | Male | Deaths | 2017 | 12.7 | 12.7 to 12.8 |
| PMP | Male | Deaths | 2018 | 12.1 | 12 to 12.1 |
| PMP | Male | Deaths | 2019 | 11.6 | 11.5 to 11.6 |
| PMP | Male | Deaths | 2020 | 11.1 | 11.1 to 11.2 |
| PMP | Male | Deaths | 2021 | 11.5 | 11.5 to 11.6 |
| PMP | Male | Deaths | 2022 | 11.5 | 11 to 11.9 |
| PMP | Male | Deaths | 2023 | 11.6 | 10.7 to 12.4 |
| PMP | Male | Deaths | 2024 | 11.6 | 10.4 to 12.9 |
| PMP | Male | Deaths | 2025 | 11.7 | 9.9 to 13.5 |
| PMP | Male | Deaths | 2026 | 11.8 | 9.4 to 14.2 |
| PMP | Male | Deaths | 2027 | 11.9 | 8.9 to 14.9 |
| PMP | Male | Deaths | 2028 | 12 | 8.2 to 15.7 |
| PMP | Male | Deaths | 2029 | 12 | 7.6 to 16.5 |
| PMP | Male | Deaths | 2030 | 12.1 | 6.8 to 17.4 |
| PMP | Female | Deaths | 1990 | 5.9 | 5.8 to 5.9 |
| PMP | Female | Deaths | 1991 | 5.9 | 5.8 to 5.9 |
| PMP | Female | Deaths | 1992 | 5.9 | 5.9 to 6 |
| PMP | Female | Deaths | 1993 | 6 | 5.9 to 6 |
| PMP | Female | Deaths | 1994 | 6 | 6 to 6 |
| PMP | Female | Deaths | 1995 | 6 | 6 to 6.1 |
| PMP | Female | Deaths | 1996 | 6 | 6 to 6.1 |
| PMP | Female | Deaths | 1997 | 6 | 6 to 6.1 |
| PMP | Female | Deaths | 1998 | 6 | 6 to 6.1 |
| PMP | Female | Deaths | 1999 | 6.1 | 6 to 6.1 |
| PMP | Female | Deaths | 2000 | 6.1 | 6.1 to 6.1 |
| PMP | Female | Deaths | 2001 | 6.1 | 6 to 6.1 |
| PMP | Female | Deaths | 2002 | 6 | 6 to 6.1 |

| | | | | | |
|---|---|---|---|---|---|
| PMP | Female | Deaths | 2003 | 6 | 6 to 6.1 |
| PMP | Female | Deaths | 2004 | 6 | 6 to 6 |
| PMP | Female | Deaths | 2005 | 5.9 | 5.9 to 6 |
| PMP | Female | Deaths | 2006 | 5.8 | 5.7 to 5.8 |
| PMP | Female | Deaths | 2007 | 5.6 | 5.6 to 5.7 |
| PMP | Female | Deaths | 2008 | 5.5 | 5.5 to 5.6 |
| PMP | Female | Deaths | 2009 | 5.4 | 5.4 to 5.5 |
| PMP | Female | Deaths | 2010 | 5.4 | 5.3 to 5.4 |
| PMP | Female | Deaths | 2011 | 5.3 | 5.3 to 5.3 |
| PMP | Female | Deaths | 2012 | 5.2 | 5.2 to 5.2 |
| PMP | Female | Deaths | 2013 | 5.1 | 5.1 to 5.1 |
| PMP | Female | Deaths | 2014 | 5.1 | 5 to 5.1 |
| PMP | Female | Deaths | 2015 | 5 | 5 to 5 |
| PMP | Female | Deaths | 2016 | 5 | 4.9 to 5 |
| PMP | Female | Deaths | 2017 | 4.9 | 4.8 to 4.9 |
| PMP | Female | Deaths | 2018 | 4.7 | 4.7 to 4.8 |
| PMP | Female | Deaths | 2019 | 4.6 | 4.6 to 4.7 |
| PMP | Female | Deaths | 2020 | 4.6 | 4.5 to 4.6 |
| PMP | Female | Deaths | 2021 | 4.7 | 4.7 to 4.8 |
| PMP | Female | Deaths | 2022 | 4.8 | 4.7 to 5 |
| PMP | Female | Deaths | 2023 | 4.9 | 4.6 to 5.2 |
| PMP | Female | Deaths | 2024 | 5.1 | 4.6 to 5.5 |
| PMP | Female | Deaths | 2025 | 5.2 | 4.5 to 5.8 |
| PMP | Female | Deaths | 2026 | 5.3 | 4.4 to 6.2 |
| PMP | Female | Deaths | 2027 | 5.4 | 4.2 to 6.6 |
| PMP | Female | Deaths | 2028 | 5.5 | 4.1 to 7 |
| PMP | Female | Deaths | 2029 | 5.6 | 3.9 to 7.4 |
| PMP | Female | Deaths | 2030 | 5.7 | 3.6 to 7.9 |
| PMP | Both | Deaths | 1990 | 11.6 | 11.3 to 11.9 |

| | | | | | |
|---|---|---|---|---|---|
| PMP | Both | Deaths | 1991 | 11.5 | 11.2 to 11.8 |
| PMP | Both | Deaths | 1992 | 11.5 | 11.2 to 11.8 |
| PMP | Both | Deaths | 1993 | 11.5 | 11.2 to 11.7 |
| PMP | Both | Deaths | 1994 | 11.4 | 11.2 to 11.7 |
| PMP | Both | Deaths | 1995 | 11.4 | 11.2 to 11.7 |
| PMP | Both | Deaths | 1996 | 11.3 | 11 to 11.6 |
| PMP | Both | Deaths | 1997 | 11.2 | 10.9 to 11.5 |
| PMP | Both | Deaths | 1998 | 11.1 | 10.9 to 11.4 |
| PMP | Both | Deaths | 1999 | 11.1 | 10.9 to 11.4 |
| PMP | Both | Deaths | 2000 | 11.2 | 11 to 11.5 |
| PMP | Both | Deaths | 2001 | 11.2 | 10.9 to 11.4 |
| PMP | Both | Deaths | 2002 | 11.1 | 10.8 to 11.3 |
| PMP | Both | Deaths | 2003 | 11 | 10.8 to 11.3 |
| PMP | Both | Deaths | 2004 | 11 | 10.7 to 11.2 |
| PMP | Both | Deaths | 2005 | 10.8 | 10.6 to 11 |
| PMP | Both | Deaths | 2006 | 10.4 | 10.2 to 10.7 |
| PMP | Both | Deaths | 2007 | 10.2 | 10 to 10.4 |
| PMP | Both | Deaths | 2008 | 10 | 9.8 to 10.2 |
| PMP | Both | Deaths | 2009 | 9.9 | 9.7 to 10.1 |
| PMP | Both | Deaths | 2010 | 9.8 | 9.5 to 10 |
| PMP | Both | Deaths | 2011 | 9.7 | 9.5 to 9.9 |
| PMP | Both | Deaths | 2012 | 9.5 | 9.3 to 9.7 |
| PMP | Both | Deaths | 2013 | 9.3 | 9.1 to 9.5 |
| PMP | Both | Deaths | 2014 | 9.1 | 8.9 to 9.3 |
| PMP | Both | Deaths | 2015 | 9 | 8.8 to 9.2 |
| PMP | Both | Deaths | 2016 | 8.8 | 8.6 to 9 |
| PMP | Both | Deaths | 2017 | 8.4 | 8.3 to 8.6 |
| PMP | Both | Deaths | 2018 | 8.1 | 7.9 to 8.3 |
| PMP | Both | Deaths | 2019 | 7.8 | 7.7 to 8 |

| | | | | | |
|---|---|---|---|---|---|
| PMP | Both | Deaths | 2020 | 7.6 | 7.4 to 7.7 |
| PMP | Both | Deaths | 2021 | 7.8 | 7.7 to 8 |
| PMP | Both | Deaths | 2022 | 7.9 | 7.4 to 8.3 |
| PMP | Both | Deaths | 2023 | 8 | 7.3 to 8.6 |
| PMP | Both | Deaths | 2024 | 8.1 | 7.2 to 9 |
| PMP | Both | Deaths | 2025 | 8.2 | 6.9 to 9.5 |
| PMP | Both | Deaths | 2026 | 8.3 | 6.7 to 10 |
| PMP | Both | Deaths | 2027 | 8.5 | 6.3 to 10.6 |
| PMP | Both | Deaths | 2028 | 8.6 | 5.9 to 11.2 |
| PMP | Both | Deaths | 2029 | 8.7 | 5.5 to 11.9 |
| PMP | Both | Deaths | 2030 | 8.9 | 5 to 12.7 |
| APMP | Male | Deaths | 1990 | 10 | 10 to 10.1 |
| APMP | Male | Deaths | 1991 | 9.9 | 9.9 to 10 |
| APMP | Male | Deaths | 1992 | 9.9 | 9.8 to 9.9 |
| APMP | Male | Deaths | 1993 | 9.9 | 9.8 to 10 |
| APMP | Male | Deaths | 1994 | 9.8 | 9.8 to 9.9 |
| APMP | Male | Deaths | 1995 | 9.8 | 9.7 to 9.8 |
| APMP | Male | Deaths | 1996 | 9.6 | 9.6 to 9.7 |
| APMP | Male | Deaths | 1997 | 9.5 | 9.5 to 9.6 |
| APMP | Male | Deaths | 1998 | 9.5 | 9.4 to 9.6 |
| APMP | Male | Deaths | 1999 | 9.5 | 9.5 to 9.6 |
| APMP | Male | Deaths | 2000 | 9.6 | 9.6 to 9.7 |
| APMP | Male | Deaths | 2001 | 9.7 | 9.6 to 9.7 |
| APMP | Male | Deaths | 2002 | 9.7 | 9.7 to 9.8 |
| APMP | Male | Deaths | 2003 | 9.8 | 9.8 to 9.9 |
| APMP | Male | Deaths | 2004 | 9.9 | 9.9 to 10 |
| APMP | Male | Deaths | 2005 | 9.9 | 9.9 to 10 |
| APMP | Male | Deaths | 2006 | 9.8 | 9.7 to 9.8 |
| APMP | Male | Deaths | 2007 | 9.7 | 9.7 to 9.8 |

| | | | | |
|---|---|---|---|---|
| APMP Male | Deaths | 2008 | 9.8 | 9.8 to 9.9 |
| APMP Male | Deaths | 2009 | 10 | 9.9 to 10 |
| APMP Male | Deaths | 2010 | 10.1 | 10.1 to 10.2 |
| APMP Male | Deaths | 2011 | 10.3 | 10.2 to 10.3 |
| APMP Male | Deaths | 2012 | 10.5 | 10.5 to 10.6 |
| APMP Male | Deaths | 2013 | 10.7 | 10.7 to 10.8 |
| APMP Male | Deaths | 2014 | 10.8 | 10.7 to 10.8 |
| APMP Male | Deaths | 2015 | 10.7 | 10.7 to 10.8 |
| APMP Male | Deaths | 2016 | 10.6 | 10.5 to 10.6 |
| APMP Male | Deaths | 2017 | 10.1 | 10.1 to 10.2 |
| APMP Male | Deaths | 2018 | 9.7 | 9.7 to 9.8 |
| APMP Male | Deaths | 2019 | 9.4 | 9.4 to 9.5 |
| APMP Male | Deaths | 2020 | 9.1 | 9 to 9.1 |
| APMP Male | Deaths | 2021 | 9.4 | 9.3 to 9.4 |
| APMP Male | Deaths | 2022 | 9.2 | 8.8 to 9.5 |
| APMP Male | Deaths | 2023 | 9.2 | 8.5 to 9.8 |
| APMP Male | Deaths | 2024 | 9.2 | 8.2 to 10.2 |
| APMP Male | Deaths | 2025 | 9.2 | 7.8 to 10.6 |
| APMP Male | Deaths | 2026 | 9.2 | 7.4 to 11.1 |
| APMP Male | Deaths | 2027 | 9.3 | 6.9 to 11.6 |
| APMP Male | Deaths | 2028 | 9.3 | 6.4 to 12.1 |
| APMP Male | Deaths | 2029 | 9.3 | 5.9 to 12.7 |
| APMP Male | Deaths | 2030 | 9.3 | 5.3 to 13.3 |
| APMPFemale | Deaths | 1990 | 2.5 | 2.5 to 2.5 |
| APMPFemale | Deaths | 1991 | 2.5 | 2.5 to 2.6 |
| APMPFemale | Deaths | 1992 | 2.6 | 2.6 to 2.6 |
| APMPFemale | Deaths | 1993 | 2.6 | 2.6 to 2.6 |
| APMPFemale | Deaths | 1994 | 2.6 | 2.6 to 2.7 |
| APMPFemale | Deaths | 1995 | 2.7 | 2.6 to 2.7 |

| | | | | |
|---|---|---|---|---|
| APMPFemale | Deaths | 1996 | 2.7 | 2.7 to 2.7 |
| APMPFemale | Deaths | 1997 | 2.7 | 2.7 to 2.7 |
| APMPFemale | Deaths | 1998 | 2.8 | 2.7 to 2.8 |
| APMPFemale | Deaths | 1999 | 2.8 | 2.8 to 2.8 |
| APMPFemale | Deaths | 2000 | 2.9 | 2.8 to 2.9 |
| APMPFemale | Deaths | 2001 | 2.9 | 2.9 to 2.9 |
| APMPFemale | Deaths | 2002 | 3 | 2.9 to 3 |
| APMPFemale | Deaths | 2003 | 3 | 3 to 3 |
| APMPFemale | Deaths | 2004 | 3.1 | 3.1 to 3.1 |
| APMPFemale | Deaths | 2005 | 3.1 | 3.1 to 3.1 |
| APMPFemale | Deaths | 2006 | 3.1 | 3.1 to 3.2 |
| APMPFemale | Deaths | 2007 | 3.2 | 3.1 to 3.2 |
| APMPFemale | Deaths | 2008 | 3.2 | 3.2 to 3.2 |
| APMPFemale | Deaths | 2009 | 3.2 | 3.2 to 3.3 |
| APMPFemale | Deaths | 2010 | 3.3 | 3.3 to 3.3 |
| APMPFemale | Deaths | 2011 | 3.4 | 3.4 to 3.4 |
| APMPFemale | Deaths | 2012 | 3.5 | 3.4 to 3.5 |
| APMPFemale | Deaths | 2013 | 3.6 | 3.5 to 3.6 |
| APMPFemale | Deaths | 2014 | 3.6 | 3.6 to 3.7 |
| APMPFemale | Deaths | 2015 | 3.7 | 3.7 to 3.7 |
| APMPFemale | Deaths | 2016 | 3.7 | 3.7 to 3.7 |
| APMPFemale | Deaths | 2017 | 3.6 | 3.6 to 3.7 |
| APMPFemale | Deaths | 2018 | 3.6 | 3.6 to 3.6 |
| APMPFemale | Deaths | 2019 | 3.6 | 3.6 to 3.6 |
| APMPFemale | Deaths | 2020 | 3.5 | 3.5 to 3.6 |
| APMPFemale | Deaths | 2021 | 3.7 | 3.6 to 3.7 |
| APMPFemale | Deaths | 2022 | 3.7 | 3.5 to 3.8 |
| APMPFemale | Deaths | 2023 | 3.7 | 3.5 to 3.9 |
| APMPFemale | Deaths | 2024 | 3.7 | 3.5 to 4 |

| | | | | |
|---|---|---|---|---|
| APMPFemale | Deaths | 2025 | 3.8 | 3.4 to 4.2 |
| APMPFemale | Deaths | 2026 | 3.8 | 3.3 to 4.4 |
| APMPFemale | Deaths | 2027 | 3.9 | 3.2 to 4.6 |
| APMPFemale | Deaths | 2028 | 3.9 | 3.1 to 4.8 |
| APMPFemale | Deaths | 2029 | 4 | 3 to 5 |
| APMPFemale | Deaths | 2030 | 4 | 2.8 to 5.2 |
| APMP Both | Deaths | 1990 | 5.9 | 5.7 to 6.1 |
| APMP Both | Deaths | 1991 | 5.9 | 5.7 to 6.1 |
| APMP Both | Deaths | 1992 | 5.9 | 5.7 to 6.1 |
| APMP Both | Deaths | 1993 | 5.9 | 5.7 to 6.1 |
| APMP Both | Deaths | 1994 | 5.9 | 5.7 to 6.1 |
| APMP Both | Deaths | 1995 | 5.9 | 5.7 to 6.1 |
| APMP Both | Deaths | 1996 | 5.8 | 5.6 to 6 |
| APMP Both | Deaths | 1997 | 5.8 | 5.6 to 6 |
| APMP Both | Deaths | 1998 | 5.8 | 5.6 to 6 |
| APMP Both | Deaths | 1999 | 5.8 | 5.7 to 6 |
| APMP Both | Deaths | 2000 | 5.9 | 5.7 to 6.1 |
| APMP Both | Deaths | 2001 | 6 | 5.8 to 6.1 |
| APMP Both | Deaths | 2002 | 6 | 5.8 to 6.2 |
| APMP Both | Deaths | 2003 | 6.1 | 5.9 to 6.3 |
| APMP Both | Deaths | 2004 | 6.2 | 6 to 6.3 |
| APMP Both | Deaths | 2005 | 6.2 | 6 to 6.4 |
| APMP Both | Deaths | 2006 | 6.1 | 6 to 6.3 |
| APMP Both | Deaths | 2007 | 6.1 | 6 to 6.3 |
| APMP Both | Deaths | 2008 | 6.2 | 6 to 6.4 |
| APMP Both | Deaths | 2009 | 6.3 | 6.1 to 6.5 |
| APMP Both | Deaths | 2010 | 6.4 | 6.2 to 6.6 |
| APMP Both | Deaths | 2011 | 6.5 | 6.4 to 6.7 |
| APMP Both | Deaths | 2012 | 6.7 | 6.5 to 6.8 |

| | | | | | |
|---|---|---|---|---|---|
| APMP | Both | Deaths | 2013 | 6.8 | 6.7 to 7 |
| APMP | Both | Deaths | 2014 | 6.9 | 6.7 to 7.1 |
| APMP | Both | Deaths | 2015 | 6.9 | 6.7 to 7.1 |
| APMP | Both | Deaths | 2016 | 6.8 | 6.7 to 7 |
| APMP | Both | Deaths | 2017 | 6.6 | 6.4 to 6.8 |
| APMP | Both | Deaths | 2018 | 6.4 | 6.2 to 6.5 |
| APMP | Both | Deaths | 2019 | 6.2 | 6.1 to 6.4 |
| APMP | Both | Deaths | 2020 | 6.1 | 5.9 to 6.2 |
| APMP | Both | Deaths | 2021 | 6.3 | 6.1 to 6.4 |
| APMP | Both | Deaths | 2022 | 6.2 | 5.8 to 6.6 |
| APMP | Both | Deaths | 2023 | 6.2 | 5.7 to 6.7 |
| APMP | Both | Deaths | 2024 | 6.3 | 5.6 to 7 |
| APMP | Both | Deaths | 2025 | 6.3 | 5.4 to 7.2 |
| APMP | Both | Deaths | 2026 | 6.3 | 5.1 to 7.6 |
| APMP | Both | Deaths | 2027 | 6.4 | 4.9 to 7.9 |
| APMP | Both | Deaths | 2028 | 6.5 | 4.6 to 8.3 |
| APMP | Both | Deaths | 2029 | 6.5 | 4.3 to 8.8 |
| APMP | Both | Deaths | 2030 | 6.6 | 3.9 to 9.3 |
| HAP | Male | Deaths | 1990 | 8.5 | 8.5 to 8.6 |
| HAP | Male | Deaths | 1991 | 8.4 | 8.4 to 8.5 |
| HAP | Male | Deaths | 1992 | 8.4 | 8.3 to 8.4 |
| HAP | Male | Deaths | 1993 | 8.3 | 8.2 to 8.3 |
| HAP | Male | Deaths | 1994 | 8.2 | 8.1 to 8.2 |
| HAP | Male | Deaths | 1995 | 8.1 | 8.1 to 8.2 |
| HAP | Male | Deaths | 1996 | 8 | 8 to 8.1 |
| HAP | Male | Deaths | 1997 | 7.9 | 7.9 to 8 |
| HAP | Male | Deaths | 1998 | 7.8 | 7.8 to 7.9 |
| HAP | Male | Deaths | 1999 | 7.7 | 7.7 to 7.7 |
| HAP | Male | Deaths | 2000 | 7.7 | 7.7 to 7.7 |

| | | | | | |
|---|---|---|---|---|---|
| HAP | Male | Deaths | 2001 | 7.6 | 7.5 to 7.6 |
| HAP | Male | Deaths | 2002 | 7.4 | 7.3 to 7.4 |
| HAP | Male | Deaths | 2003 | 7.2 | 7.2 to 7.3 |
| HAP | Male | Deaths | 2004 | 7 | 7 to 7.1 |
| HAP | Male | Deaths | 2005 | 6.7 | 6.7 to 6.7 |
| HAP | Male | Deaths | 2006 | 6.2 | 6.2 to 6.2 |
| HAP | Male | Deaths | 2007 | 5.8 | 5.8 to 5.9 |
| HAP | Male | Deaths | 2008 | 5.5 | 5.5 to 5.5 |
| HAP | Male | Deaths | 2009 | 5.2 | 5.2 to 5.2 |
| HAP | Male | Deaths | 2010 | 4.9 | 4.9 to 4.9 |
| HAP | Male | Deaths | 2011 | 4.5 | 4.5 to 4.6 |
| HAP | Male | Deaths | 2012 | 4.1 | 4.1 to 4.1 |
| HAP | Male | Deaths | 2013 | 3.7 | 3.6 to 3.7 |
| HAP | Male | Deaths | 2014 | 3.3 | 3.3 to 3.3 |
| HAP | Male | Deaths | 2015 | 3 | 2.9 to 3 |
| HAP | Male | Deaths | 2016 | 2.7 | 2.7 to 2.8 |
| HAP | Male | Deaths | 2017 | 2.5 | 2.5 to 2.5 |
| HAP | Male | Deaths | 2018 | 2.3 | 2.3 to 2.3 |
| HAP | Male | Deaths | 2019 | 2.2 | 2.1 to 2.2 |
| HAP | Male | Deaths | 2020 | 2 | 2 to 2.1 |
| HAP | Male | Deaths | 2021 | 2.2 | 2.1 to 2.2 |
| HAP | Male | Deaths | 2022 | 2.2 | 2.1 to 2.3 |
| HAP | Male | Deaths | 2023 | 2.2 | 2 to 2.5 |
| HAP | Male | Deaths | 2024 | 2.3 | 1.9 to 2.6 |
| HAP | Male | Deaths | 2025 | 2.3 | 1.8 to 2.8 |
| HAP | Male | Deaths | 2026 | 2.3 | 1.6 to 3.1 |
| HAP | Male | Deaths | 2027 | 2.4 | 1.5 to 3.3 |
| HAP | Male | Deaths | 2028 | 2.4 | 1.3 to 3.6 |
| HAP | Male | Deaths | 2029 | 2.5 | 1.1 to 3.9 |

| | | | | |
|---|---|---|---|---|
| HAP Male | Deaths | 2030 | 2.5 | 0.9 to 4.2 |
| HAP Female | Deaths | 1990 | 3.3 | 3.3 to 3.4 |
| HAP Female | Deaths | 1991 | 3.3 | 3.3 to 3.4 |
| HAP Female | Deaths | 1992 | 3.3 | 3.3 to 3.4 |
| HAP Female | Deaths | 1993 | 3.3 | 3.3 to 3.4 |
| HAP Female | Deaths | 1994 | 3.4 | 3.3 to 3.4 |
| HAP Female | Deaths | 1995 | 3.3 | 3.3 to 3.4 |
| HAP Female | Deaths | 1996 | 3.3 | 3.3 to 3.4 |
| HAP Female | Deaths | 1997 | 3.3 | 3.3 to 3.3 |
| HAP Female | Deaths | 1998 | 3.3 | 3.2 to 3.3 |
| HAP Female | Deaths | 1999 | 3.3 | 3.2 to 3.3 |
| HAP Female | Deaths | 2000 | 3.2 | 3.2 to 3.3 |
| HAP Female | Deaths | 2001 | 3.2 | 3.1 to 3.2 |
| HAP Female | Deaths | 2002 | 3.1 | 3.1 to 3.1 |
| HAP Female | Deaths | 2003 | 3 | 3 to 3 |
| HAP Female | Deaths | 2004 | 2.9 | 2.9 to 3 |
| HAP Female | Deaths | 2005 | 2.8 | 2.8 to 2.8 |
| HAP Female | Deaths | 2006 | 2.6 | 2.6 to 2.7 |
| HAP Female | Deaths | 2007 | 2.5 | 2.5 to 2.5 |
| HAP Female | Deaths | 2008 | 2.3 | 2.3 to 2.4 |
| HAP Female | Deaths | 2009 | 2.2 | 2.2 to 2.2 |
| HAP Female | Deaths | 2010 | 2.1 | 2 to 2.1 |
| HAP Female | Deaths | 2011 | 1.9 | 1.9 to 1.9 |
| HAP Female | Deaths | 2012 | 1.8 | 1.7 to 1.8 |
| HAP Female | Deaths | 2013 | 1.6 | 1.6 to 1.6 |
| HAP Female | Deaths | 2014 | 1.4 | 1.4 to 1.5 |
| HAP Female | Deaths | 2015 | 1.3 | 1.3 to 1.3 |
| HAP Female | Deaths | 2016 | 1.3 | 1.2 to 1.3 |
| HAP Female | Deaths | 2017 | 1.2 | 1.2 to 1.2 |

| | | | | | |
|---|---|---|---|---|---|
| HAP | Female | Deaths | 2018 | 1.1 | 1.1 to 1.1 |
| HAP | Female | Deaths | 2019 | 1.1 | 1.1 to 1.1 |
| HAP | Female | Deaths | 2020 | 1 | 1 to 1 |
| HAP | Female | Deaths | 2021 | 1.1 | 1.1 to 1.1 |
| HAP | Female | Deaths | 2022 | 1.1 | 1.1 to 1.2 |
| HAP | Female | Deaths | 2023 | 1.2 | 1.1 to 1.3 |
| HAP | Female | Deaths | 2024 | 1.2 | 1 to 1.4 |
| HAP | Female | Deaths | 2025 | 1.3 | 1 to 1.6 |
| HAP | Female | Deaths | 2026 | 1.3 | 0.9 to 1.8 |
| HAP | Female | Deaths | 2027 | 1.4 | 0.9 to 2 |
| HAP | Female | Deaths | 2028 | 1.5 | 0.8 to 2.2 |
| HAP | Female | Deaths | 2029 | 1.5 | 0.6 to 2.4 |
| HAP | Female | Deaths | 2030 | 1.6 | 0.5 to 2.7 |
| HAP | Both | Deaths | 1990 | 5.7 | 5.5 to 5.9 |
| HAP | Both | Deaths | 1991 | 5.7 | 5.5 to 5.9 |
| HAP | Both | Deaths | 1992 | 5.6 | 5.4 to 5.8 |
| HAP | Both | Deaths | 1993 | 5.6 | 5.4 to 5.8 |
| HAP | Both | Deaths | 1994 | 5.5 | 5.4 to 5.7 |
| HAP | Both | Deaths | 1995 | 5.5 | 5.4 to 5.7 |
| HAP | Both | Deaths | 1996 | 5.5 | 5.3 to 5.7 |
| HAP | Both | Deaths | 1997 | 5.4 | 5.2 to 5.6 |
| HAP | Both | Deaths | 1998 | 5.3 | 5.2 to 5.5 |
| HAP | Both | Deaths | 1999 | 5.3 | 5.1 to 5.5 |
| HAP | Both | Deaths | 2000 | 5.3 | 5.1 to 5.4 |
| HAP | Both | Deaths | 2001 | 5.2 | 5 to 5.4 |
| HAP | Both | Deaths | 2002 | 5.1 | 4.9 to 5.2 |
| HAP | Both | Deaths | 2003 | 4.9 | 4.8 to 5.1 |
| HAP | Both | Deaths | 2004 | 4.8 | 4.6 to 4.9 |
| HAP | Both | Deaths | 2005 | 4.6 | 4.4 to 4.7 |

| | | | | | |
|---|---|---|---|---|---|
| HAP | Both | Deaths | 2006 | 4.3 | 4.1 to 4.4 |
| HAP | Both | Deaths | 2007 | 4 | 3.9 to 4.2 |
| HAP | Both | Deaths | 2008 | 3.8 | 3.7 to 3.9 |
| HAP | Both | Deaths | 2009 | 3.6 | 3.4 to 3.7 |
| HAP | Both | Deaths | 2010 | 3.4 | 3.2 to 3.5 |
| HAP | Both | Deaths | 2011 | 3.1 | 3 to 3.2 |
| HAP | Both | Deaths | 2012 | 2.8 | 2.7 to 2.9 |
| HAP | Both | Deaths | 2013 | 2.5 | 2.4 to 2.6 |
| HAP | Both | Deaths | 2014 | 2.3 | 2.2 to 2.4 |
| HAP | Both | Deaths | 2015 | 2.1 | 2 to 2.2 |
| HAP | Both | Deaths | 2016 | 1.9 | 1.9 to 2 |
| HAP | Both | Deaths | 2017 | 1.8 | 1.7 to 1.9 |
| HAP | Both | Deaths | 2018 | 1.7 | 1.6 to 1.8 |
| HAP | Both | Deaths | 2019 | 1.6 | 1.5 to 1.6 |
| HAP | Both | Deaths | 2020 | 1.5 | 1.4 to 1.6 |
| HAP | Both | Deaths | 2021 | 1.6 | 1.5 to 1.7 |
| HAP | Both | Deaths | 2022 | 1.6 | 1.5 to 1.8 |
| HAP | Both | Deaths | 2023 | 1.7 | 1.5 to 1.9 |
| HAP | Both | Deaths | 2024 | 1.7 | 1.4 to 2 |
| HAP | Both | Deaths | 2025 | 1.8 | 1.3 to 2.2 |
| HAP | Both | Deaths | 2026 | 1.8 | 1.2 to 2.4 |
| HAP | Both | Deaths | 2027 | 1.9 | 1.1 to 2.7 |
| HAP | Both | Deaths | 2028 | 2 | 1 to 3 |
| HAP | Both | Deaths | 2029 | 2.1 | 0.8 to 3.3 |
| HAP | Both | Deaths | 2030 | 2.2 | 0.6 to 3.7 |
| PMP | Male | DALYs (Disability-Adjusted Life Years) | 1990 | 467.2 | 466.8 to 467.6 |
| PMP | Male | DALYs (Disability-Adjusted Life Years) | 1991 | 461.8 | 461.4 to 462.2 |
| PMP | Male | DALYs (Disability-Adjusted Life Years) | 1992 | 457.1 | 456.7 to 457.5 |
| PMP | Male | DALYs (Disability-Adjusted Life Years) | 1993 | 454.2 | 453.8 to 454.6 |

| | | | | | |
|---|---|---|---|---|---|
| PMP | Male | DALYs (Disability-Adjusted Life Years) | 1994 | 448.8 | 448.4 to 449.2 |
| PMP | Male | DALYs (Disability-Adjusted Life Years) | 1995 | 446.8 | 446.4 to 447.2 |
| PMP | Male | DALYs (Disability-Adjusted Life Years) | 1996 | 438.7 | 438.3 to 439.1 |
| PMP | Male | DALYs (Disability-Adjusted Life Years) | 1997 | 431.1 | 430.7 to 431.5 |
| PMP | Male | DALYs (Disability-Adjusted Life Years) | 1998 | 425.6 | 425.2 to 426 |
| PMP | Male | DALYs (Disability-Adjusted Life Years) | 1999 | 422.1 | 421.7 to 422.4 |
| PMP | Male | DALYs (Disability-Adjusted Life Years) | 2000 | 423.4 | 423 to 423.8 |
| PMP | Male | DALYs (Disability-Adjusted Life Years) | 2001 | 419.3 | 418.9 to 419.6 |
| PMP | Male | DALYs (Disability-Adjusted Life Years) | 2002 | 414.2 | 413.8 to 414.5 |
| PMP | Male | DALYs (Disability-Adjusted Life Years) | 2003 | 410.3 | 409.9 to 410.6 |
| PMP | Male | DALYs (Disability-Adjusted Life Years) | 2004 | 406.4 | 406.1 to 406.7 |
| PMP | Male | DALYs (Disability-Adjusted Life Years) | 2005 | 398.8 | 398.5 to 399.1 |
| PMP | Male | DALYs (Disability-Adjusted Life Years) | 2006 | 383.3 | 383 to 383.6 |
| PMP | Male | DALYs (Disability-Adjusted Life Years) | 2007 | 372.4 | 372.1 to 372.7 |
| PMP | Male | DALYs (Disability-Adjusted Life Years) | 2008 | 365.9 | 365.6 to 366.2 |
| PMP | Male | DALYs (Disability-Adjusted Life Years) | 2009 | 359.2 | 358.9 to 359.5 |
| PMP | Male | DALYs (Disability-Adjusted Life Years) | 2010 | 353.8 | 353.5 to 354 |
| PMP | Male | DALYs (Disability-Adjusted Life Years) | 2011 | 349.1 | 348.8 to 349.4 |
| PMP | Male | DALYs (Disability-Adjusted Life Years) | 2012 | 343.9 | 343.6 to 344.1 |
| PMP | Male | DALYs (Disability-Adjusted Life Years) | 2013 | 336 | 335.7 to 336.3 |
| PMP | Male | DALYs (Disability-Adjusted Life Years) | 2014 | 326.6 | 326.4 to 326.9 |
| PMP | Male | DALYs (Disability-Adjusted Life Years) | 2015 | 319.4 | 319.1 to 319.6 |
| PMP | Male | DALYs (Disability-Adjusted Life Years) | 2016 | 310.3 | 310.1 to 310.6 |
| PMP | Male | DALYs (Disability-Adjusted Life Years) | 2017 | 295.1 | 294.9 to 295.4 |
| PMP | Male | DALYs (Disability-Adjusted Life Years) | 2018 | 280.5 | 280.3 to 280.8 |
| PMP | Male | DALYs (Disability-Adjusted Life Years) | 2019 | 268.4 | 268.2 to 268.6 |
| PMP | Male | DALYs (Disability-Adjusted Life Years) | 2020 | 256.6 | 256.4 to 256.8 |
| PMP | Male | DALYs (Disability-Adjusted Life Years) | 2021 | 266.1 | 265.9 to 266.3 |
| PMP | Male | DALYs (Disability-Adjusted Life Years) | 2022 | 262.6 | 251.6 to 273.5 |

| | | | | | |
|---|---|---|---|---|---|
| PMP | Male | DALYs (Disability-Adjusted Life Years) | 2023 | 262.7 | 243.9 to 281.5 |
| PMP | Male | DALYs (Disability-Adjusted Life Years) | 2024 | 262.7 | 234.1 to 291.4 |
| PMP | Male | DALYs (Disability-Adjusted Life Years) | 2025 | 262.7 | 223 to 302.5 |
| PMP | Male | DALYs (Disability-Adjusted Life Years) | 2026 | 262.9 | 210.9 to 315 |
| PMP | Male | DALYs (Disability-Adjusted Life Years) | 2027 | 263 | 197.6 to 328.4 |
| PMP | Male | DALYs (Disability-Adjusted Life Years) | 2028 | 263 | 183.3 to 342.7 |
| PMP | Male | DALYs (Disability-Adjusted Life Years) | 2029 | 262.8 | 168 to 357.5 |
| PMP | Male | DALYs (Disability-Adjusted Life Years) | 2030 | 262.6 | 152 to 373.2 |
| PMP | Female | DALYs (Disability-Adjusted Life Years) | 1990 | 151.7 | 151.5 to 151.9 |
| PMP | Female | DALYs (Disability-Adjusted Life Years) | 1991 | 152.1 | 151.9 to 152.3 |
| PMP | Female | DALYs (Disability-Adjusted Life Years) | 1992 | 152.3 | 152.1 to 152.5 |
| PMP | Female | DALYs (Disability-Adjusted Life Years) | 1993 | 153.1 | 152.8 to 153.3 |
| PMP | Female | DALYs (Disability-Adjusted Life Years) | 1994 | 153.6 | 153.4 to 153.8 |
| PMP | Female | DALYs (Disability-Adjusted Life Years) | 1995 | 153.9 | 153.7 to 154.2 |
| PMP | Female | DALYs (Disability-Adjusted Life Years) | 1996 | 153.4 | 153.2 to 153.6 |
| PMP | Female | DALYs (Disability-Adjusted Life Years) | 1997 | 152.7 | 152.5 to 152.9 |
| PMP | Female | DALYs (Disability-Adjusted Life Years) | 1998 | 152.4 | 152.2 to 152.6 |
| PMP | Female | DALYs (Disability-Adjusted Life Years) | 1999 | 152.6 | 152.4 to 152.8 |
| PMP | Female | DALYs (Disability-Adjusted Life Years) | 2000 | 153.3 | 153.1 to 153.5 |
| PMP | Female | DALYs (Disability-Adjusted Life Years) | 2001 | 152.2 | 152 to 152.4 |
| PMP | Female | DALYs (Disability-Adjusted Life Years) | 2002 | 150.9 | 150.7 to 151.1 |
| PMP | Female | DALYs (Disability-Adjusted Life Years) | 2003 | 149.7 | 149.6 to 149.9 |
| PMP | Female | DALYs (Disability-Adjusted Life Years) | 2004 | 148.9 | 148.7 to 149.1 |
| PMP | Female | DALYs (Disability-Adjusted Life Years) | 2005 | 146.8 | 146.6 to 147 |
| PMP | Female | DALYs (Disability-Adjusted Life Years) | 2006 | 142.8 | 142.6 to 143 |
| PMP | Female | DALYs (Disability-Adjusted Life Years) | 2007 | 139.3 | 139.1 to 139.4 |
| PMP | Female | DALYs (Disability-Adjusted Life Years) | 2008 | 135.9 | 135.7 to 136.1 |
| PMP | Female | DALYs (Disability-Adjusted Life Years) | 2009 | 132.9 | 132.7 to 133 |
| PMP | Female | DALYs (Disability-Adjusted Life Years) | 2010 | 130.5 | 130.4 to 130.7 |

| | | | | | |
|---|---|---|---|---|---|
| PMP | Female | DALYs (Disability-Adjusted Life Years) | 2011 | 128.9 | 128.7 to 129.1 |
| PMP | Female | DALYs (Disability-Adjusted Life Years) | 2012 | 126.2 | 126 to 126.3 |
| PMP | Female | DALYs (Disability-Adjusted Life Years) | 2013 | 123.6 | 123.5 to 123.8 |
| PMP | Female | DALYs (Disability-Adjusted Life Years) | 2014 | 121.9 | 121.7 to 122 |
| PMP | Female | DALYs (Disability-Adjusted Life Years) | 2015 | 120.3 | 120.2 to 120.4 |
| PMP | Female | DALYs (Disability-Adjusted Life Years) | 2016 | 118.8 | 118.7 to 119 |
| PMP | Female | DALYs (Disability-Adjusted Life Years) | 2017 | 116 | 115.8 to 116.1 |
| PMP | Female | DALYs (Disability-Adjusted Life Years) | 2018 | 112.8 | 112.7 to 113 |
| PMP | Female | DALYs (Disability-Adjusted Life Years) | 2019 | 110 | 109.9 to 110.2 |
| PMP | Female | DALYs (Disability-Adjusted Life Years) | 2020 | 107.1 | 106.9 to 107.2 |
| PMP | Female | DALYs (Disability-Adjusted Life Years) | 2021 | 112.3 | 112.1 to 112.4 |
| PMP | Female | DALYs (Disability-Adjusted Life Years) | 2022 | 113.4 | 109.7 to 117.1 |
| PMP | Female | DALYs (Disability-Adjusted Life Years) | 2023 | 115.7 | 108.7 to 122.6 |
| PMP | Female | DALYs (Disability-Adjusted Life Years) | 2024 | 117.8 | 106.9 to 128.8 |
| PMP | Female | DALYs (Disability-Adjusted Life Years) | 2025 | 120 | 104.4 to 135.6 |
| PMP | Female | DALYs (Disability-Adjusted Life Years) | 2026 | 122.3 | 101.3 to 143.2 |
| PMP | Female | DALYs (Disability-Adjusted Life Years) | 2027 | 124.6 | 97.6 to 151.5 |
| PMP | Female | DALYs (Disability-Adjusted Life Years) | 2028 | 126.8 | 93.2 to 160.3 |
| PMP | Female | DALYs (Disability-Adjusted Life Years) | 2029 | 128.9 | 88.2 to 169.6 |
| PMP | Female | DALYs (Disability-Adjusted Life Years) | 2030 | 131 | 82.6 to 179.4 |
| PMP | Both | DALYs (Disability-Adjusted Life Years) | 1990 | 299.8 | 298.3 to 301.3 |
| PMP | Both | DALYs (Disability-Adjusted Life Years) | 1991 | 297.7 | 296.2 to 299.1 |
| PMP | Both | DALYs (Disability-Adjusted Life Years) | 1992 | 295.7 | 294.3 to 297.2 |
| PMP | Both | DALYs (Disability-Adjusted Life Years) | 1993 | 294.9 | 293.4 to 296.3 |
| PMP | Both | DALYs (Disability-Adjusted Life Years) | 1994 | 292.7 | 291.3 to 294.2 |
| PMP | Both | DALYs (Disability-Adjusted Life Years) | 1995 | 292 | 290.6 to 293.4 |
| PMP | Both | DALYs (Disability-Adjusted Life Years) | 1996 | 287.9 | 286.6 to 289.3 |
| PMP | Both | DALYs (Disability-Adjusted Life Years) | 1997 | 283.9 | 282.6 to 285.3 |
| PMP | Both | DALYs (Disability-Adjusted Life Years) | 1998 | 281.2 | 279.8 to 282.5 |

| | | | | |
|---|---|---|---|---|
| PMP | Both | DALYs (Disability-Adjusted Life Years)1999 | 279.6 | 278.3 to 280.9 |
| PMP | Both | DALYs (Disability-Adjusted Life Years)2000 | 280.6 | 279.3 to 281.9 |
| PMP | Both | DALYs (Disability-Adjusted Life Years)2001 | 278.1 | 276.8 to 279.3 |
| PMP | Both | DALYs (Disability-Adjusted Life Years)2002 | 275 | 273.7 to 276.3 |
| PMP | Both | DALYs (Disability-Adjusted Life Years)2003 | 272.5 | 271.2 to 273.7 |
| PMP | Both | DALYs (Disability-Adjusted Life Years)2004 | 270.2 | 269 to 271.4 |
| PMP | Both | DALYs (Disability-Adjusted Life Years)2005 | 265.6 | 264.4 to 266.8 |
| PMP | Both | DALYs (Disability-Adjusted Life Years)2006 | 256.2 | 255.1 to 257.4 |
| PMP | Both | DALYs (Disability-Adjusted Life Years)2007 | 249.3 | 248.2 to 250.4 |
| PMP | Both | DALYs (Disability-Adjusted Life Years)2008 | 244.5 | 243.3 to 245.6 |
| PMP | Both | DALYs (Disability-Adjusted Life Years)2009 | 239.7 | 238.6 to 240.7 |
| PMP | Both | DALYs (Disability-Adjusted Life Years)2010 | 235.9 | 234.8 to 236.9 |
| PMP | Both | DALYs (Disability-Adjusted Life Years)2011 | 232.9 | 231.8 to 233.9 |
| PMP | Both | DALYs (Disability-Adjusted Life Years)2012 | 229 | 228 to 230 |
| PMP | Both | DALYs (Disability-Adjusted Life Years)2013 | 223.9 | 222.9 to 224.9 |
| PMP | Both | DALYs (Disability-Adjusted Life Years)2014 | 218.5 | 217.5 to 219.5 |
| PMP | Both | DALYs (Disability-Adjusted Life Years)2015 | 214.3 | 213.3 to 215.2 |
| PMP | Both | DALYs (Disability-Adjusted Life Years)2016 | 209.3 | 208.3 to 210.2 |
| PMP | Both | DALYs (Disability-Adjusted Life Years)2017 | 200.6 | 199.7 to 201.5 |
| PMP | Both | DALYs (Disability-Adjusted Life Years)2018 | 192.1 | 191.2 to 193 |
| PMP | Both | DALYs (Disability-Adjusted Life Years)2019 | 184.9 | 184.1 to 185.8 |
| PMP | Both | DALYs (Disability-Adjusted Life Years)2020 | 177.7 | 176.9 to 178.5 |
| PMP | Both | DALYs (Disability-Adjusted Life Years)2021 | 184.9 | 184 to 185.7 |
| PMP | Both | DALYs (Disability-Adjusted Life Years)2022 | 184 | 171.6 to 196.3 |
| PMP | Both | DALYs (Disability-Adjusted Life Years)2023 | 185.3 | 168.9 to 201.7 |
| PMP | Both | DALYs (Disability-Adjusted Life Years)2024 | 186.6 | 164.3 to 208.8 |
| PMP | Both | DALYs (Disability-Adjusted Life Years)2025 | 188 | 158.4 to 217.5 |
| PMP | Both | DALYs (Disability-Adjusted Life Years)2026 | 189.7 | 151.5 to 227.8 |
| PMP | Both | DALYs (Disability-Adjusted Life Years)2027 | 191.4 | 143.5 to 239.4 |

| | | | | | |
|---|---|---|---|---|---|
| PMP | Both | DALYs (Disability-Adjusted Life Years) | 2028 | 193.3 | 134.4 to 252.3 |
| PMP | Both | DALYs (Disability-Adjusted Life Years) | 2029 | 195.3 | 124.2 to 266.4 |
| PMP | Both | DALYs (Disability-Adjusted Life Years) | 2030 | 197.5 | 113.1 to 281.9 |
| APMP | Male | DALYs (Disability-Adjusted Life Years) | 1990 | 245.2 | 244.9 to 245.5 |
| APMP | Male | DALYs (Disability-Adjusted Life Years) | 1991 | 242.3 | 242 to 242.6 |
| APMP | Male | DALYs (Disability-Adjusted Life Years) | 1992 | 240.6 | 240.3 to 240.9 |
| APMP | Male | DALYs (Disability-Adjusted Life Years) | 1993 | 240.6 | 240.3 to 240.9 |
| APMP | Male | DALYs (Disability-Adjusted Life Years) | 1994 | 238.2 | 237.9 to 238.5 |
| APMP | Male | DALYs (Disability-Adjusted Life Years) | 1995 | 237 | 236.7 to 237.3 |
| APMP | Male | DALYs (Disability-Adjusted Life Years) | 1996 | 232.1 | 231.8 to 232.3 |
| APMP | Male | DALYs (Disability-Adjusted Life Years) | 1997 | 228.4 | 228.1 to 228.6 |
| APMP | Male | DALYs (Disability-Adjusted Life Years) | 1998 | 226.9 | 226.6 to 227.1 |
| APMP | Male | DALYs (Disability-Adjusted Life Years) | 1999 | 227 | 226.7 to 227.3 |
| APMP | Male | DALYs (Disability-Adjusted Life Years) | 2000 | 228.9 | 228.6 to 229.2 |
| APMP | Male | DALYs (Disability-Adjusted Life Years) | 2001 | 228.5 | 228.2 to 228.7 |
| APMP | Male | DALYs (Disability-Adjusted Life Years) | 2002 | 228.8 | 228.6 to 229.1 |
| APMP | Male | DALYs (Disability-Adjusted Life Years) | 2003 | 230.3 | 230 to 230.6 |
| APMP | Male | DALYs (Disability-Adjusted Life Years) | 2004 | 232.2 | 231.9 to 232.4 |
| APMP | Male | DALYs (Disability-Adjusted Life Years) | 2005 | 232.3 | 232.1 to 232.6 |
| APMP | Male | DALYs (Disability-Adjusted Life Years) | 2006 | 228.3 | 228 to 228.5 |
| APMP | Male | DALYs (Disability-Adjusted Life Years) | 2007 | 227.2 | 227 to 227.5 |
| APMP | Male | DALYs (Disability-Adjusted Life Years) | 2008 | 229.1 | 228.9 to 229.4 |
| APMP | Male | DALYs (Disability-Adjusted Life Years) | 2009 | 231.1 | 230.9 to 231.3 |
| APMP | Male | DALYs (Disability-Adjusted Life Years) | 2010 | 233.2 | 233 to 233.4 |
| APMP | Male | DALYs (Disability-Adjusted Life Years) | 2011 | 237.1 | 236.9 to 237.4 |
| APMP | Male | DALYs (Disability-Adjusted Life Years) | 2012 | 242.8 | 242.5 to 243 |
| APMP | Male | DALYs (Disability-Adjusted Life Years) | 2013 | 246.6 | 246.3 to 246.8 |
| APMP | Male | DALYs (Disability-Adjusted Life Years) | 2014 | 247 | 246.7 to 247.2 |
| APMP | Male | DALYs (Disability-Adjusted Life Years) | 2015 | 245.7 | 245.5 to 245.9 |

| | | | | | |
|---|---|---|---|---|---|
| APMP | Male | DALYs (Disability-Adjusted Life Years) | 2016 | 240.8 | 240.6 to 241.1 |
| APMP | Male | DALYs (Disability-Adjusted Life Years) | 2017 | 230.9 | 230.6 to 231.1 |
| APMP | Male | DALYs (Disability-Adjusted Life Years) | 2018 | 221.4 | 221.2 to 221.6 |
| APMP | Male | DALYs (Disability-Adjusted Life Years) | 2019 | 214 | 213.8 to 214.2 |
| APMP | Male | DALYs (Disability-Adjusted Life Years) | 2020 | 205.4 | 205.2 to 205.5 |
| APMP | Male | DALYs (Disability-Adjusted Life Years) | 2021 | 211.7 | 211.5 to 211.9 |
| APMP | Male | DALYs (Disability-Adjusted Life Years) | 2022 | 204.6 | 195.2 to 214 |
| APMP | Male | DALYs (Disability-Adjusted Life Years) | 2023 | 203.5 | 188.3 to 218.6 |
| APMP | Male | DALYs (Disability-Adjusted Life Years) | 2024 | 202.3 | 179.9 to 224.6 |
| APMP | Male | DALYs (Disability-Adjusted Life Years) | 2025 | 201 | 170.4 to 231.7 |
| APMP | Male | DALYs (Disability-Adjusted Life Years) | 2026 | 199.9 | 160.2 to 239.6 |
| APMP | Male | DALYs (Disability-Adjusted Life Years) | 2027 | 198.8 | 149.3 to 248.4 |
| APMP | Male | DALYs (Disability-Adjusted Life Years) | 2028 | 197.8 | 137.8 to 257.8 |
| APMP | Male | DALYs (Disability-Adjusted Life Years) | 2029 | 196.7 | 125.8 to 267.7 |
| APMP | Male | DALYs (Disability-Adjusted Life Years) | 2030 | 195.7 | 113.2 to 278.1 |
| APMP | Female | DALYs (Disability-Adjusted Life Years) | 1990 | 61.3 | 61.2 to 61.5 |
| APMP | Female | DALYs (Disability-Adjusted Life Years) | 1991 | 61.8 | 61.7 to 61.9 |
| APMP | Female | DALYs (Disability-Adjusted Life Years) | 1992 | 62.3 | 62.2 to 62.5 |
| APMP | Female | DALYs (Disability-Adjusted Life Years) | 1993 | 63.1 | 63 to 63.2 |
| APMP | Female | DALYs (Disability-Adjusted Life Years) | 1994 | 63.7 | 63.5 to 63.8 |
| APMP | Female | DALYs (Disability-Adjusted Life Years) | 1995 | 64.4 | 64.3 to 64.6 |
| APMP | Female | DALYs (Disability-Adjusted Life Years) | 1996 | 64.6 | 64.5 to 64.8 |
| APMP | Female | DALYs (Disability-Adjusted Life Years) | 1997 | 65.1 | 65 to 65.3 |
| APMP | Female | DALYs (Disability-Adjusted Life Years) | 1998 | 65.8 | 65.7 to 66 |
| APMP | Female | DALYs (Disability-Adjusted Life Years) | 1999 | 66.8 | 66.7 to 67 |
| APMP | Female | DALYs (Disability-Adjusted Life Years) | 2000 | 68.3 | 68.2 to 68.4 |
| APMP | Female | DALYs (Disability-Adjusted Life Years) | 2001 | 69.2 | 69 to 69.3 |
| APMP | Female | DALYs (Disability-Adjusted Life Years) | 2002 | 70.3 | 70.2 to 70.4 |
| APMP | Female | DALYs (Disability-Adjusted Life Years) | 2003 | 71.6 | 71.5 to 71.7 |

| | | |
|---|---|---|
| APMPFemaleDALYs (Disability-Adjusted Life Years)2004 | 73.1 | 73 to 73.3 |
| APMPFemaleDALYs (Disability-Adjusted Life Years)2005 | 74.2 | 74 to 74.3 |
| APMPFemaleDALYs (Disability-Adjusted Life Years)2006 | 74.5 | 74.3 to 74.6 |
| APMPFemaleDALYs (Disability-Adjusted Life Years)2007 | 75.1 | 75 to 75.3 |
| APMPFemaleDALYs (Disability-Adjusted Life Years)2008 | 75.9 | 75.7 to 76 |
| APMPFemaleDALYs (Disability-Adjusted Life Years)2009 | 76.8 | 76.7 to 76.9 |
| APMPFemaleDALYs (Disability-Adjusted Life Years)2010 | 77.7 | 77.6 to 77.8 |
| APMPFemaleDALYs (Disability-Adjusted Life Years)2011 | 79.8 | 79.7 to 79.9 |
| APMPFemaleDALYs (Disability-Adjusted Life Years)2012 | 81.7 | 81.6 to 81.9 |
| APMPFemaleDALYs (Disability-Adjusted Life Years)2013 | 83.8 | 83.6 to 83.9 |
| APMPFemaleDALYs (Disability-Adjusted Life Years)2014 | 85.7 | 85.5 to 85.8 |
| APMPFemaleDALYs (Disability-Adjusted Life Years)2015 | 86.1 | 86 to 86.2 |
| APMPFemaleDALYs (Disability-Adjusted Life Years)2016 | 86 | 85.9 to 86.1 |
| APMPFemaleDALYs (Disability-Adjusted Life Years)2017 | 84.7 | 84.6 to 84.8 |
| APMPFemaleDALYs (Disability-Adjusted Life Years)2018 | 83.6 | 83.5 to 83.7 |
| APMPFemaleDALYs (Disability-Adjusted Life Years)2019 | 82.7 | 82.6 to 82.8 |
| APMPFemaleDALYs (Disability-Adjusted Life Years)2020 | 80.9 | 80.8 to 81 |
| APMPFemaleDALYs (Disability-Adjusted Life Years)2021 | 84.1 | 84 to 84.2 |
| APMPFemaleDALYs (Disability-Adjusted Life Years)2022 | 83.2 | 80.3 to 86 |
| APMPFemaleDALYs (Disability-Adjusted Life Years)2023 | 83.7 | 79.2 to 88.2 |
| APMPFemaleDALYs (Disability-Adjusted Life Years)2024 | 84.2 | 77.6 to 90.8 |
| APMPFemaleDALYs (Disability-Adjusted Life Years)2025 | 84.7 | 75.7 to 93.7 |
| APMPFemaleDALYs (Disability-Adjusted Life Years)2026 | 85.2 | 73.4 to 97 |
| APMPFemaleDALYs (Disability-Adjusted Life Years)2027 | 85.7 | 70.8 to 100.5 |
| APMPFemaleDALYs (Disability-Adjusted Life Years)2028 | 86.2 | 68 to 104.3 |
| APMPFemaleDALYs (Disability-Adjusted Life Years)2029 | 86.6 | 65 to 108.2 |
| APMPFemaleDALYs (Disability-Adjusted Life Years)2030 | 87 | 61.6 to 112.3 |
| APMP Both DALYs (Disability-Adjusted Life Years)1990 | 147.4 | 146.3 to 148.4 |
| APMP Both DALYs (Disability-Adjusted Life Years)1991 | 146.4 | 145.3 to 147.4 |

| | | | | | |
|---|---|---|---|---|---|
| APMP | Both | DALYs (Disability-Adjusted Life Years) | 1992 | 146 | 144.9 to 147 |
| APMP | Both | DALYs (Disability-Adjusted Life Years) | 1993 | 146.5 | 145.5 to 147.5 |
| APMP | Both | DALYs (Disability-Adjusted Life Years) | 1994 | 145.7 | 144.7 to 146.7 |
| APMP | Both | DALYs (Disability-Adjusted Life Years) | 1995 | 145.6 | 144.6 to 146.6 |
| APMP | Both | DALYs (Disability-Adjusted Life Years) | 1996 | 143.4 | 142.4 to 144.3 |
| APMP | Both | DALYs (Disability-Adjusted Life Years) | 1997 | 141.9 | 140.9 to 142.8 |
| APMP | Both | DALYs (Disability-Adjusted Life Years) | 1998 | 141.5 | 140.6 to 142.5 |
| APMP | Both | DALYs (Disability-Adjusted Life Years) | 1999 | 142.1 | 141.2 to 143.1 |
| APMP | Both | DALYs (Disability-Adjusted Life Years) | 2000 | 143.8 | 142.9 to 144.7 |
| APMP | Both | DALYs (Disability-Adjusted Life Years) | 2001 | 144 | 143.1 to 144.9 |
| APMP | Both | DALYs (Disability-Adjusted Life Years) | 2002 | 144.8 | 143.9 to 145.7 |
| APMP | Both | DALYs (Disability-Adjusted Life Years) | 2003 | 146.2 | 145.2 to 147.1 |
| APMP | Both | DALYs (Disability-Adjusted Life Years) | 2004 | 147.9 | 147 to 148.8 |
| APMP | Both | DALYs (Disability-Adjusted Life Years) | 2005 | 148.5 | 147.6 to 149.4 |
| APMP | Both | DALYs (Disability-Adjusted Life Years) | 2006 | 146.8 | 145.9 to 147.7 |
| APMP | Both | DALYs (Disability-Adjusted Life Years) | 2007 | 146.7 | 145.8 to 147.6 |
| APMP | Both | DALYs (Disability-Adjusted Life Years) | 2008 | 148 | 147.2 to 148.9 |
| APMP | Both | DALYs (Disability-Adjusted Life Years) | 2009 | 149.4 | 148.6 to 150.3 |
| APMP | Both | DALYs (Disability-Adjusted Life Years) | 2010 | 150.9 | 150.1 to 151.7 |
| APMP | Both | DALYs (Disability-Adjusted Life Years) | 2011 | 153.9 | 153.1 to 154.7 |
| APMP | Both | DALYs (Disability-Adjusted Life Years) | 2012 | 157.6 | 156.8 to 158.5 |
| APMP | Both | DALYs (Disability-Adjusted Life Years) | 2013 | 160.5 | 159.6 to 161.3 |
| APMP | Both | DALYs (Disability-Adjusted Life Years) | 2014 | 161.6 | 160.8 to 162.5 |
| APMP | Both | DALYs (Disability-Adjusted Life Years) | 2015 | 161.3 | 160.5 to 162.1 |
| APMP | Both | DALYs (Disability-Adjusted Life Years) | 2016 | 159 | 158.2 to 159.8 |
| APMP | Both | DALYs (Disability-Adjusted Life Years) | 2017 | 153.6 | 152.8 to 154.4 |
| APMP | Both | DALYs (Disability-Adjusted Life Years) | 2018 | 148.6 | 147.8 to 149.4 |
| APMP | Both | DALYs (Disability-Adjusted Life Years) | 2019 | 144.6 | 143.9 to 145.4 |
| APMP | Both | DALYs (Disability-Adjusted Life Years) | 2020 | 139.6 | 138.9 to 140.3 |

| | | | | |
|---|---|---|---|---|
| APMP | Both | DALYs (Disability-Adjusted Life Years)2021 | 144.2 | 143.5 to 144.9 |
| APMP | Both | DALYs (Disability-Adjusted Life Years)2022 | 140.6 | 129.4 to 151.7 |
| APMP | Both | DALYs (Disability-Adjusted Life Years)2023 | 140.3 | 126.8 to 153.9 |
| APMP | Both | DALYs (Disability-Adjusted Life Years)2024 | 140.2 | 123 to 157.3 |
| APMP | Both | DALYs (Disability-Adjusted Life Years)2025 | 140 | 118.2 to 161.8 |
| APMP | Both | DALYs (Disability-Adjusted Life Years)2026 | 140 | 112.7 to 167.4 |
| APMP | Both | DALYs (Disability-Adjusted Life Years)2027 | 140.1 | 106.5 to 173.8 |
| APMP | Both | DALYs (Disability-Adjusted Life Years)2028 | 140.4 | 99.8 to 181 |
| APMP | Both | DALYs (Disability-Adjusted Life Years)2029 | 140.7 | 92.4 to 189 |
| APMP | Both | DALYs (Disability-Adjusted Life Years)2030 | 141.2 | 84.5 to 197.8 |
| HAP | Male | DALYs (Disability-Adjusted Life Years)1990 | 221.9 | 221.6 to 222.2 |
| HAP | Male | DALYs (Disability-Adjusted Life Years)1991 | 219.5 | 219.2 to 219.7 |
| HAP | Male | DALYs (Disability-Adjusted Life Years)1992 | 216.6 | 216.3 to 216.9 |
| HAP | Male | DALYs (Disability-Adjusted Life Years)1993 | 213.7 | 213.4 to 214 |
| HAP | Male | DALYs (Disability-Adjusted Life Years)1994 | 210.7 | 210.4 to 210.9 |
| HAP | Male | DALYs (Disability-Adjusted Life Years)1995 | 209.7 | 209.4 to 209.9 |
| HAP | Male | DALYs (Disability-Adjusted Life Years)1996 | 206.5 | 206.2 to 206.7 |
| HAP | Male | DALYs (Disability-Adjusted Life Years)1997 | 202.6 | 202.4 to 202.9 |
| HAP | Male | DALYs (Disability-Adjusted Life Years)1998 | 198.8 | 198.5 to 199 |
| HAP | Male | DALYs (Disability-Adjusted Life Years)1999 | 195.2 | 194.9 to 195.4 |
| HAP | Male | DALYs (Disability-Adjusted Life Years)2000 | 194.4 | 194.1 to 194.6 |
| HAP | Male | DALYs (Disability-Adjusted Life Years)2001 | 190.6 | 190.3 to 190.8 |
| HAP | Male | DALYs (Disability-Adjusted Life Years)2002 | 185.2 | 185 to 185.5 |
| HAP | Male | DALYs (Disability-Adjusted Life Years)2003 | 180.1 | 179.9 to 180.3 |
| HAP | Male | DALYs (Disability-Adjusted Life Years)2004 | 174.4 | 174.2 to 174.6 |
| HAP | Male | DALYs (Disability-Adjusted Life Years)2005 | 166.4 | 166.2 to 166.6 |
| HAP | Male | DALYs (Disability-Adjusted Life Years)2006 | 154.6 | 154.4 to 154.8 |
| HAP | Male | DALYs (Disability-Adjusted Life Years)2007 | 145 | 144.8 to 145.2 |
| HAP | Male | DALYs (Disability-Adjusted Life Years)2008 | 136.8 | 136.6 to 137 |

| | | | | | |
|---|---|---|---|---|---|
| HAP | Male | DALYs (Disability-Adjusted Life Years) | 2009 | 128.3 | 128.1 to 128.4 |
| HAP | Male | DALYs (Disability-Adjusted Life Years) | 2010 | 120.5 | 120.3 to 120.6 |
| HAP | Male | DALYs (Disability-Adjusted Life Years) | 2011 | 111.9 | 111.7 to 112.1 |
| HAP | Male | DALYs (Disability-Adjusted Life Years) | 2012 | 101.8 | 101.6 to 101.9 |
| HAP | Male | DALYs (Disability-Adjusted Life Years) | 2013 | 90.8 | 90.7 to 90.9 |
| HAP | Male | DALYs (Disability-Adjusted Life Years) | 2014 | 81 | 80.9 to 81.1 |
| HAP | Male | DALYs (Disability-Adjusted Life Years) | 2015 | 73.6 | 73.5 to 73.7 |
| HAP | Male | DALYs (Disability-Adjusted Life Years) | 2016 | 67.9 | 67.8 to 68 |
| HAP | Male | DALYs (Disability-Adjusted Life Years) | 2017 | 62.5 | 62.4 to 62.6 |
| HAP | Male | DALYs (Disability-Adjusted Life Years) | 2018 | 58 | 57.9 to 58.1 |
| HAP | Male | DALYs (Disability-Adjusted Life Years) | 2019 | 54.3 | 54.2 to 54.4 |
| HAP | Male | DALYs (Disability-Adjusted Life Years) | 2020 | 51.2 | 51.1 to 51.3 |
| HAP | Male | DALYs (Disability-Adjusted Life Years) | 2021 | 54.3 | 54.2 to 54.4 |
| HAP | Male | DALYs (Disability-Adjusted Life Years) | 2022 | 53.7 | 50.5 to 56.9 |
| HAP | Male | DALYs (Disability-Adjusted Life Years) | 2023 | 54.5 | 48.7 to 60.3 |
| HAP | Male | DALYs (Disability-Adjusted Life Years) | 2024 | 55.3 | 46.3 to 64.3 |
| HAP | Male | DALYs (Disability-Adjusted Life Years) | 2025 | 56.2 | 43.3 to 69 |
| HAP | Male | DALYs (Disability-Adjusted Life Years) | 2026 | 57 | 39.8 to 74.3 |
| HAP | Male | DALYs (Disability-Adjusted Life Years) | 2027 | 57.9 | 35.8 to 80 |
| HAP | Male | DALYs (Disability-Adjusted Life Years) | 2028 | 58.8 | 31.4 to 86.1 |
| HAP | Male | DALYs (Disability-Adjusted Life Years) | 2029 | 59.6 | 26.4 to 92.8 |
| HAP | Male | DALYs (Disability-Adjusted Life Years) | 2030 | 60.6 | 21.1 to 100.1 |
| HAP | Female | DALYs (Disability-Adjusted Life Years) | 1990 | 90.4 | 90.2 to 90.6 |
| HAP | Female | DALYs (Disability-Adjusted Life Years) | 1991 | 90.3 | 90.2 to 90.5 |
| HAP | Female | DALYs (Disability-Adjusted Life Years) | 1992 | 90 | 89.8 to 90.2 |
| HAP | Female | DALYs (Disability-Adjusted Life Years) | 1993 | 90 | 89.8 to 90.2 |
| HAP | Female | DALYs (Disability-Adjusted Life Years) | 1994 | 89.9 | 89.8 to 90.1 |
| HAP | Female | DALYs (Disability-Adjusted Life Years) | 1995 | 89.5 | 89.3 to 89.6 |
| HAP | Female | DALYs (Disability-Adjusted Life Years) | 1996 | 88.7 | 88.5 to 88.9 |

| | | | |
|---|---|---|---|
| HAP | FemaleDALYs (Disability-Adjusted Life Years)1997 | 87.5 | 87.4 to 87.7 |
| HAP | FemaleDALYs (Disability-Adjusted Life Years)1998 | 86.6 | 86.4 to 86.7 |
| HAP | FemaleDALYs (Disability-Adjusted Life Years)1999 | 85.8 | 85.6 to 85.9 |
| HAP | FemaleDALYs (Disability-Adjusted Life Years)2000 | 84.9 | 84.8 to 85.1 |
| HAP | FemaleDALYs (Disability-Adjusted Life Years)2001 | 83 | 82.8 to 83.1 |
| HAP | FemaleDALYs (Disability-Adjusted Life Years)2002 | 80.6 | 80.5 to 80.7 |
| HAP | FemaleDALYs (Disability-Adjusted Life Years)2003 | 78.2 | 78 to 78.3 |
| HAP | FemaleDALYs (Disability-Adjusted Life Years)2004 | 75.8 | 75.6 to 75.9 |
| HAP | FemaleDALYs (Disability-Adjusted Life Years)2005 | 72.6 | 72.5 to 72.7 |
| HAP | FemaleDALYs (Disability-Adjusted Life Years)2006 | 68.2 | 68.1 to 68.3 |
| HAP | FemaleDALYs (Disability-Adjusted Life Years)2007 | 64.1 | 64 to 64.2 |
| HAP | FemaleDALYs (Disability-Adjusted Life Years)2008 | 60.1 | 60 to 60.2 |
| HAP | FemaleDALYs (Disability-Adjusted Life Years)2009 | 56.1 | 56 to 56.2 |
| HAP | FemaleDALYs (Disability-Adjusted Life Years)2010 | 52.8 | 52.7 to 52.9 |
| HAP | FemaleDALYs (Disability-Adjusted Life Years)2011 | 49.1 | 49 to 49.2 |
| HAP | FemaleDALYs (Disability-Adjusted Life Years)2012 | 44.7 | 44.7 to 44.8 |
| HAP | FemaleDALYs (Disability-Adjusted Life Years)2013 | 40.5 | 40.4 to 40.5 |
| HAP | FemaleDALYs (Disability-Adjusted Life Years)2014 | 36.8 | 36.7 to 36.8 |
| HAP | FemaleDALYs (Disability-Adjusted Life Years)2015 | 34.1 | 34.1 to 34.2 |
| HAP | FemaleDALYs (Disability-Adjusted Life Years)2016 | 32.2 | 32.1 to 32.2 |
| HAP | FemaleDALYs (Disability-Adjusted Life Years)2017 | 30.4 | 30.4 to 30.5 |
| HAP | FemaleDALYs (Disability-Adjusted Life Years)2018 | 28.8 | 28.7 to 28.8 |
| HAP | FemaleDALYs (Disability-Adjusted Life Years)2019 | 27.3 | 27.3 to 27.4 |
| HAP | FemaleDALYs (Disability-Adjusted Life Years)2020 | 26.1 | 26 to 26.2 |
| HAP | FemaleDALYs (Disability-Adjusted Life Years)2021 | 28.1 | 28 to 28.2 |
| HAP | FemaleDALYs (Disability-Adjusted Life Years)2022 | 28.9 | 27.4 to 30.5 |
| HAP | FemaleDALYs (Disability-Adjusted Life Years)2023 | 30.3 | 27.1 to 33.4 |
| HAP | FemaleDALYs (Disability-Adjusted Life Years)2024 | 31.6 | 26.4 to 36.8 |
| HAP | FemaleDALYs (Disability-Adjusted Life Years)2025 | 33.1 | 25.4 to 40.9 |

| | | | | | |
|---|---|---|---|---|---|
| HAP | Female | DALYs (Disability-Adjusted Life Years) | 2026 | 34.7 | 23.9 to 45.5 |
| HAP | Female | DALYs (Disability-Adjusted Life Years) | 2027 | 36.3 | 21.9 to 50.7 |
| HAP | Female | DALYs (Disability-Adjusted Life Years) | 2028 | 38 | 19.5 to 56.5 |
| HAP | Female | DALYs (Disability-Adjusted Life Years) | 2029 | 39.7 | 16.5 to 63 |
| HAP | Female | DALYs (Disability-Adjusted Life Years) | 2030 | 41.6 | 13 to 70.2 |
| HAP | Both | DALYs (Disability-Adjusted Life Years) | 1990 | 152.4 | 151.3 to 153.5 |
| HAP | Both | DALYs (Disability-Adjusted Life Years) | 1991 | 151.3 | 150.2 to 152.3 |
| HAP | Both | DALYs (Disability-Adjusted Life Years) | 1992 | 149.8 | 148.8 to 150.9 |
| HAP | Both | DALYs (Disability-Adjusted Life Years) | 1993 | 148.5 | 147.5 to 149.5 |
| HAP | Both | DALYs (Disability-Adjusted Life Years) | 1994 | 147 | 146 to 148 |
| HAP | Both | DALYs (Disability-Adjusted Life Years) | 1995 | 146.4 | 145.3 to 147.4 |
| HAP | Both | DALYs (Disability-Adjusted Life Years) | 1996 | 144.4 | 143.5 to 145.4 |
| HAP | Both | DALYs (Disability-Adjusted Life Years) | 1997 | 142 | 141 to 143 |
| HAP | Both | DALYs (Disability-Adjusted Life Years) | 1998 | 139.7 | 138.7 to 140.6 |
| HAP | Both | DALYs (Disability-Adjusted Life Years) | 1999 | 137.6 | 136.6 to 138.5 |
| HAP | Both | DALYs (Disability-Adjusted Life Years) | 2000 | 136.7 | 135.8 to 137.6 |
| HAP | Both | DALYs (Disability-Adjusted Life Years) | 2001 | 133.9 | 133 to 134.8 |
| HAP | Both | DALYs (Disability-Adjusted Life Years) | 2002 | 130.1 | 129.2 to 131 |
| HAP | Both | DALYs (Disability-Adjusted Life Years) | 2003 | 126.4 | 125.5 to 127.2 |
| HAP | Both | DALYs (Disability-Adjusted Life Years) | 2004 | 122.4 | 121.6 to 123.3 |
| HAP | Both | DALYs (Disability-Adjusted Life Years) | 2005 | 117 | 116.2 to 117.8 |
| HAP | Both | DALYs (Disability-Adjusted Life Years) | 2006 | 109.2 | 108.4 to 110 |
| HAP | Both | DALYs (Disability-Adjusted Life Years) | 2007 | 102.5 | 101.8 to 103.2 |
| HAP | Both | DALYs (Disability-Adjusted Life Years) | 2008 | 96.5 | 95.8 to 97.2 |
| HAP | Both | DALYs (Disability-Adjusted Life Years) | 2009 | 90.4 | 89.7 to 91 |
| HAP | Both | DALYs (Disability-Adjusted Life Years) | 2010 | 84.9 | 84.3 to 85.5 |
| HAP | Both | DALYs (Disability-Adjusted Life Years) | 2011 | 78.9 | 78.3 to 79.5 |
| HAP | Both | DALYs (Disability-Adjusted Life Years) | 2012 | 71.9 | 71.3 to 72.4 |
| HAP | Both | DALYs (Disability-Adjusted Life Years) | 2013 | 64.4 | 63.9 to 64.9 |

| | | | | | |
|---|---|---|---|---|---|
| HAP | Both | DALYs (Disability-Adjusted Life Years) | 2014 | 57.8 | 57.3 to 58.3 |
| HAP | Both | DALYs (Disability-Adjusted Life Years) | 2015 | 52.9 | 52.4 to 53.4 |
| HAP | Both | DALYs (Disability-Adjusted Life Years) | 2016 | 49.2 | 48.7 to 49.6 |
| HAP | Both | DALYs (Disability-Adjusted Life Years) | 2017 | 45.7 | 45.3 to 46.1 |
| HAP | Both | DALYs (Disability-Adjusted Life Years) | 2018 | 42.7 | 42.3 to 43.1 |
| HAP | Both | DALYs (Disability-Adjusted Life Years) | 2019 | 40.2 | 39.8 to 40.6 |
| HAP | Both | DALYs (Disability-Adjusted Life Years) | 2020 | 38.1 | 37.7 to 38.5 |
| HAP | Both | DALYs (Disability-Adjusted Life Years) | 2021 | 40.6 | 40.2 to 41 |
| HAP | Both | DALYs (Disability-Adjusted Life Years) | 2022 | 40.8 | 37.1 to 44.5 |
| HAP | Both | DALYs (Disability-Adjusted Life Years) | 2023 | 41.9 | 36.5 to 47.2 |
| HAP | Both | DALYs (Disability-Adjusted Life Years) | 2024 | 43.1 | 35.3 to 50.9 |
| HAP | Both | DALYs (Disability-Adjusted Life Years) | 2025 | 44.4 | 33.5 to 55.4 |
| HAP | Both | DALYs (Disability-Adjusted Life Years) | 2026 | 45.9 | 31.1 to 60.7 |
| HAP | Both | DALYs (Disability-Adjusted Life Years) | 2027 | 47.5 | 28.1 to 66.9 |
| HAP | Both | DALYs (Disability-Adjusted Life Years) | 2028 | 49.3 | 24.5 to 74.2 |
| HAP | Both | DALYs (Disability-Adjusted Life Years) | 2029 | 51.4 | 20.1 to 82.7 |
| HAP | Both | DALYs (Disability-Adjusted Life Years) | 2030 | 53.7 | 14.8 to 92.7 |

Abbreviation: PMP, particulate matter pollution; AMPM, ambient particulate matter pollution; HAP, household air pollution from solid fuels; DALY, Disability-adjusted life years.

# Supplementary Figures

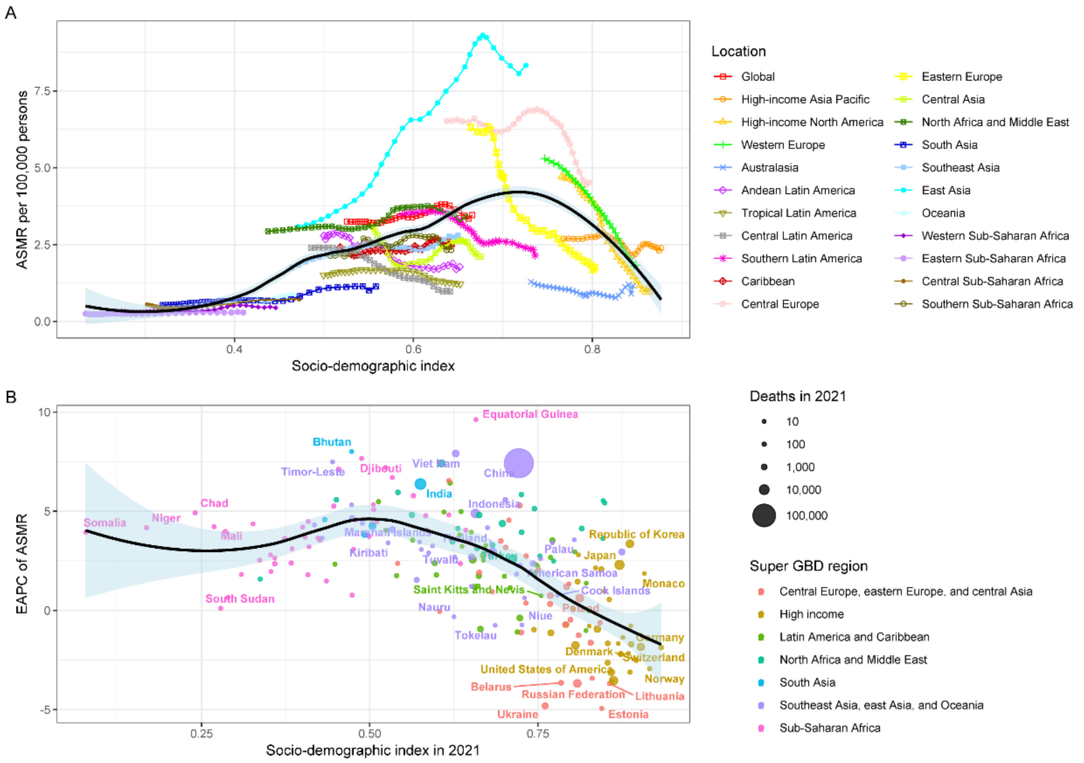


Supplement Figure 1: The correlation between APMP attributable lung cancer in ASMR and SDI (A), between EAPC in ASMR and ASMR in 2021. Abbreviation: APMP, ambient particulate matter pollution. EAPC, estimated annual percentage change; ASMR, age-standardized mortality rate; SDI, sociodemographic index.

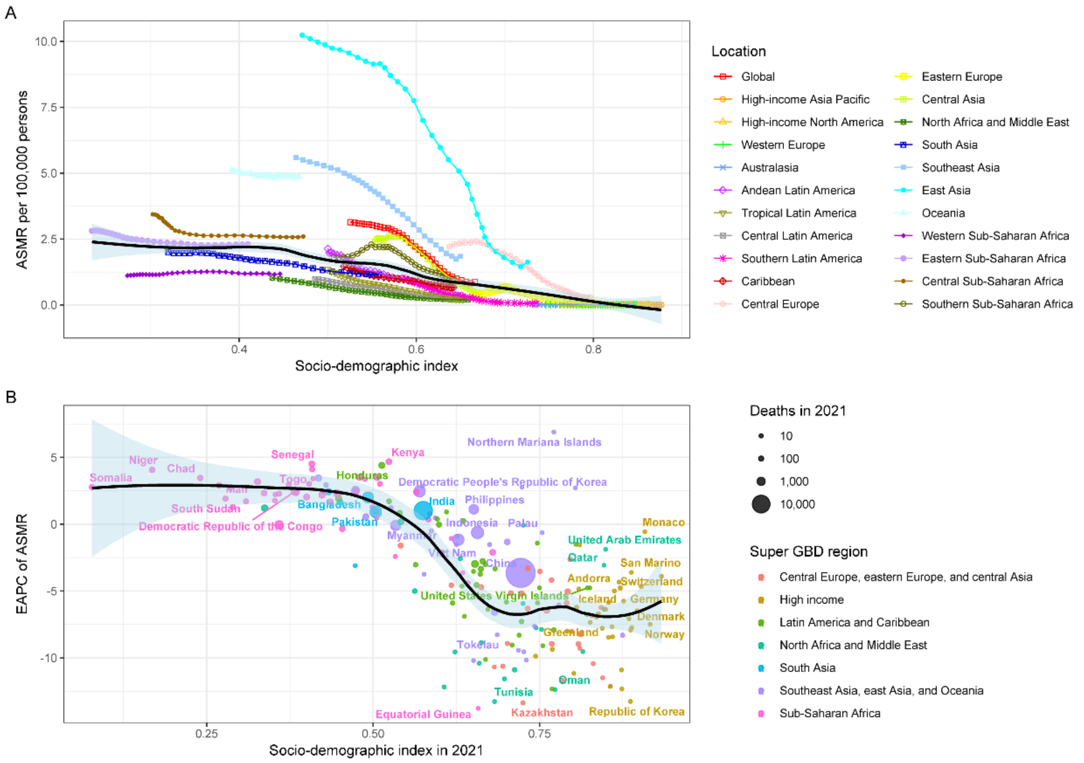


Supplement Figure 2: The correlation between HAP attributable lung cancer in ASMR and SDI (A), between EAPC in ASMR and ASMR in 2021. Abbreviation: HAP, household air pollution from solid fuels. EAPC, estimated annual percentage change; ASMR, age-standardized mortality rate; SDI, sociodemographic index.

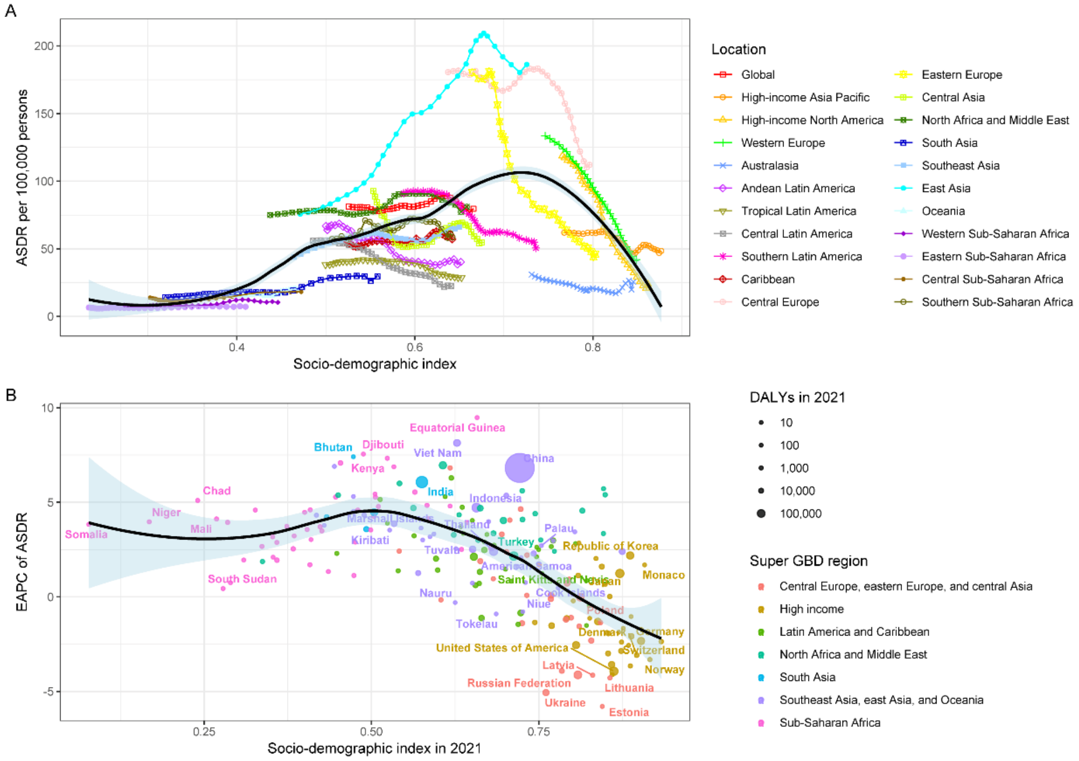


Supplement Figure 3: The correlation between APMP attributable lung cancer in ASDR and SDI (A), between EAPC in ASDR and ASDR in 2021. Abbreviation: APMP, ambient particulate matter pollution. EAPC, estimated annual percentage change; ASDR, Age-standardized DALY rate. SDI, sociodemographic index. DALY, Disability-adjusted life years years.

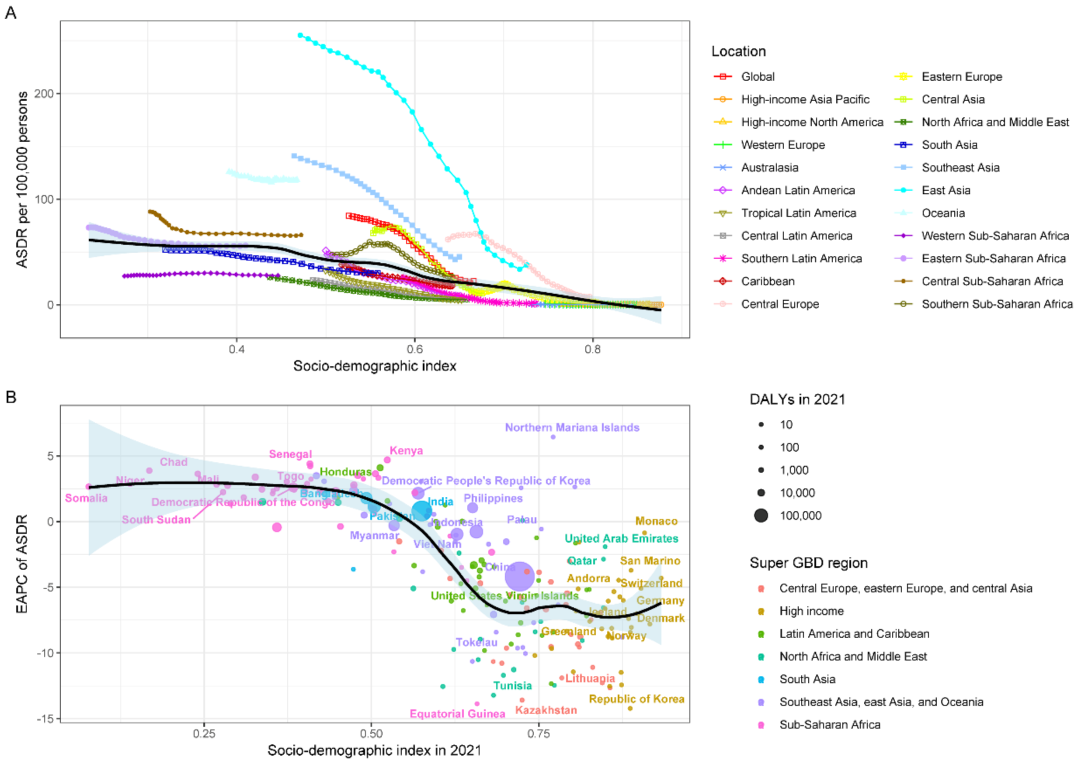


Supplement Figure 4: The correlation between HAP attributable lung cancer in ASDR and SDI (A), between EAPC in ASDR and ASDR in 2021. Abbreviation: HAP, household air pollution from solid fuels. EAPC, estimated annual percentage change; ASDR, Age-standardized DALY rate. SDI, sociodemographic index. DALY, Disability-adjusted life years.

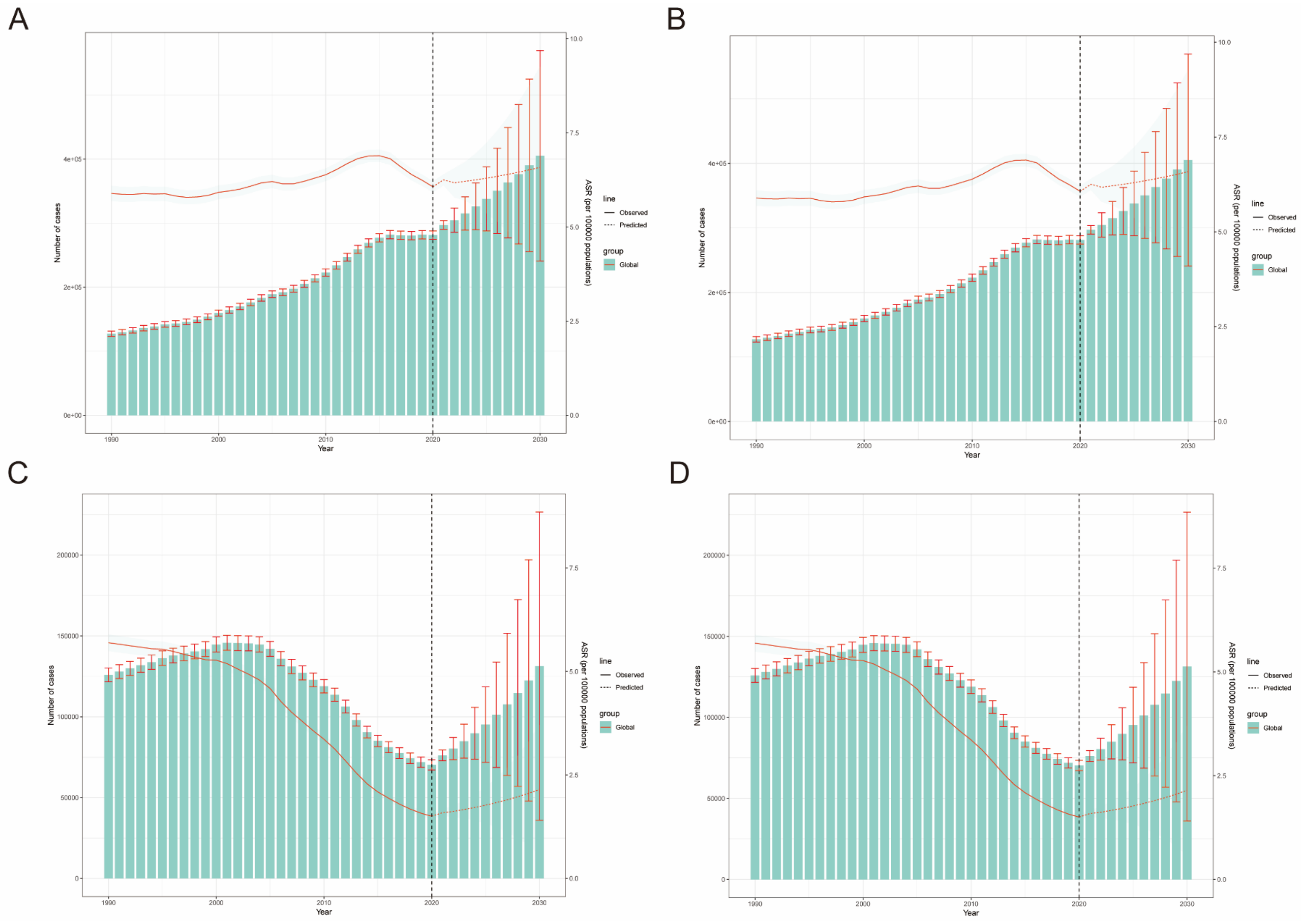
A
B
C
D
Number of cases
ASR (per 100000 populations)
Year
line
Observed
Predicted
group
Global
0e+00
2e+05
4e+05
0
50000
100000
150000
200000
0.0
2.5
5.0
7.5
10.0
1990
2000
2010
2020
2030

Supplement Figure 5: Global trends of lung cancer attributable to ambient particulate matter pollution in age-standardized death (A, C) and DALYs (B, D) rates (per 100,000 population) from 2019 to 2030 by BAPC models: observed (solid lines) and predicted rates (dashed lines).

BAPC: Bayesian age-period-cohort; DALYs: Disability-adjusted life years years; GBD: Global Burden of Disease.
